# Supplementary material for: Computational modeling and synthesis of pyridine variants of benzoyl-phenoxy-acetamide with high glioblastoma cytotoxicity and brain tumor penetration
Source: Sci Rep. 2023 Jul 28;13:12236. doi: 10.1038/s41598-023-39236-w (PMC10382599; doi:10.1038/s41598-023-39236-w)
Supplement: Supplementary file 1 — Supplementary Information. [file 41598_2023_39236_MOESM1_ESM.docx]

***Supplementary Materials***

**Computational modeling and synthesis of Pyridine variants of Benzoyl-Phenoxy-Acetamide with high glioblastoma cytotoxicity and brain tumor penetration**

Charles H. Ingraham IV^a,c^, Joanna Stalinska^c,d^, Sean C. Carson^a^, Susan B. Colley^c1^, Monika Rak^c,d^, Adam Lassak^c^, Krzysztof Reiss^c,e*^, and Branko S. Jursic^a,b*^

*^a^Department of Chemistry, University of New Orleans, New Orleans, LA 70148, United States;*

*^b^Stepharm llc., PO Box 24220, New Orleans, LA 70184;*

*^c^Neurological Cancer Research, ^c1^Stanley S. Scott Cancer Center, Department of Interdisciplinary Oncology, LSU Health Sciences Center, New Orleans, LA 70112; ^d^Department of Cell Biology, Faculty of Biochemistry, Biophysics and Biotechnology, Jagiellonian University, Cracow Poland;*

*^e^WayPath Pharma, New Orleans BioInnovation Center (NOBIC), 1441 Canal Str., New Orleans, LA 70112.*

**Corresponding authors*

*Content table:*

1. *Nuclear Magnetic Resonance data of Pyridine-BPA variants:*

*^1^H-NMR, ^13^C-NMRs (HR48-HR90). (pages 1 – 56)*

1. *MarvinSketch (Product version 22.21) data: comparison between selected chemotherapy drugs for CNS tumors and Pyridine-BPA variants (HR48-HR90). (pages 57 – 149)*
2. *Density Functional Theory (DFT) with ωB97X-D/6-31G* computed molecular properties of Pyridine-BPA variants (HR48-HR90). (pages 150 - 153)*
3. *Detection of our prototype drug candidate, PP1, in mouse tissues following intraperitoneal delivery (page 154).*
4. *Computed properties of BioTransformer 3.0 predicted phase I metabolites of HR67 (Panel A) and HR68 (Panel B) (page 155).*
5. ***Nuclear Magnetic Resonance data of Pyridine-BPA variants:  ^1^H-NMR, ^13^C-NMRs.***


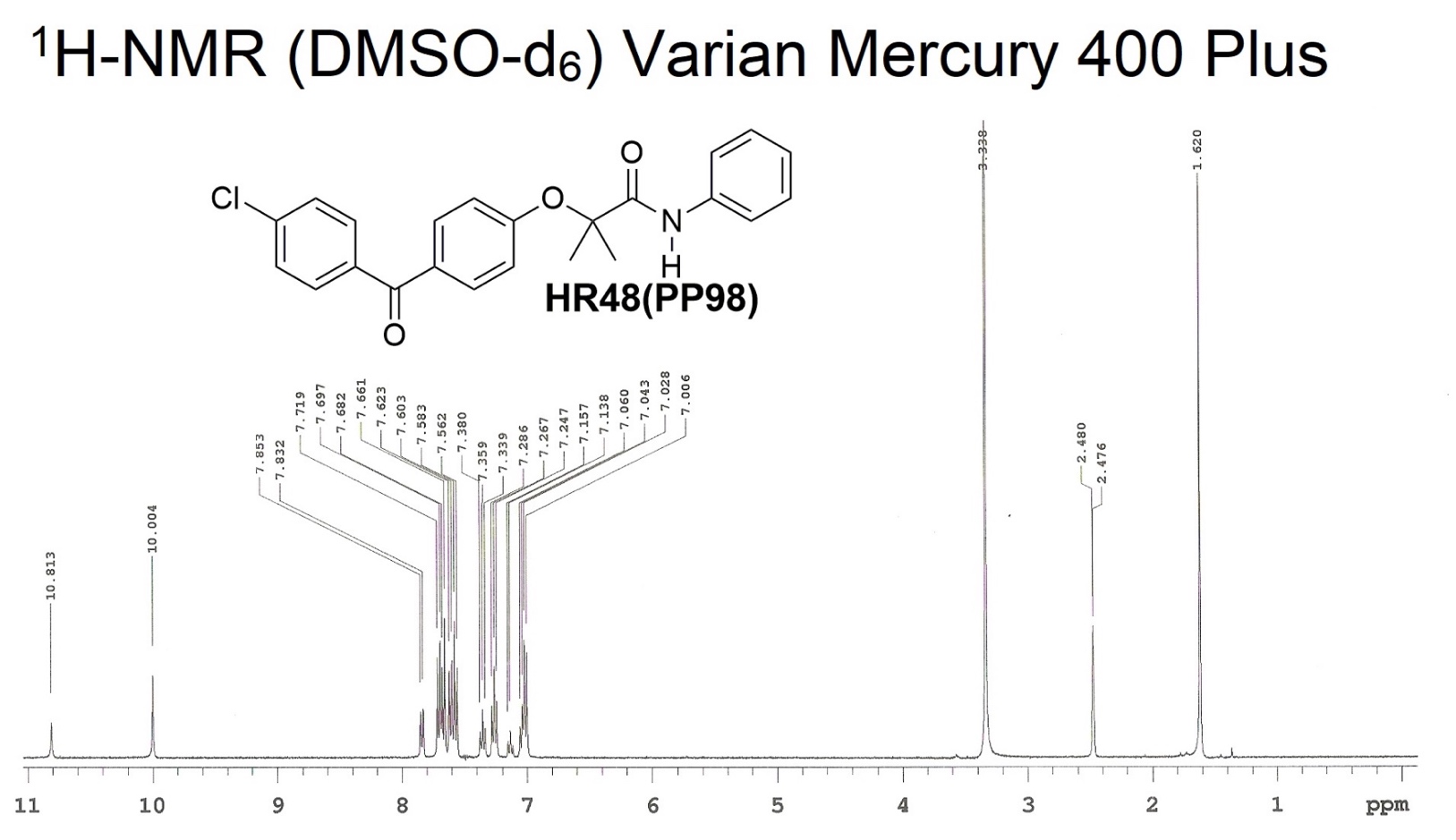


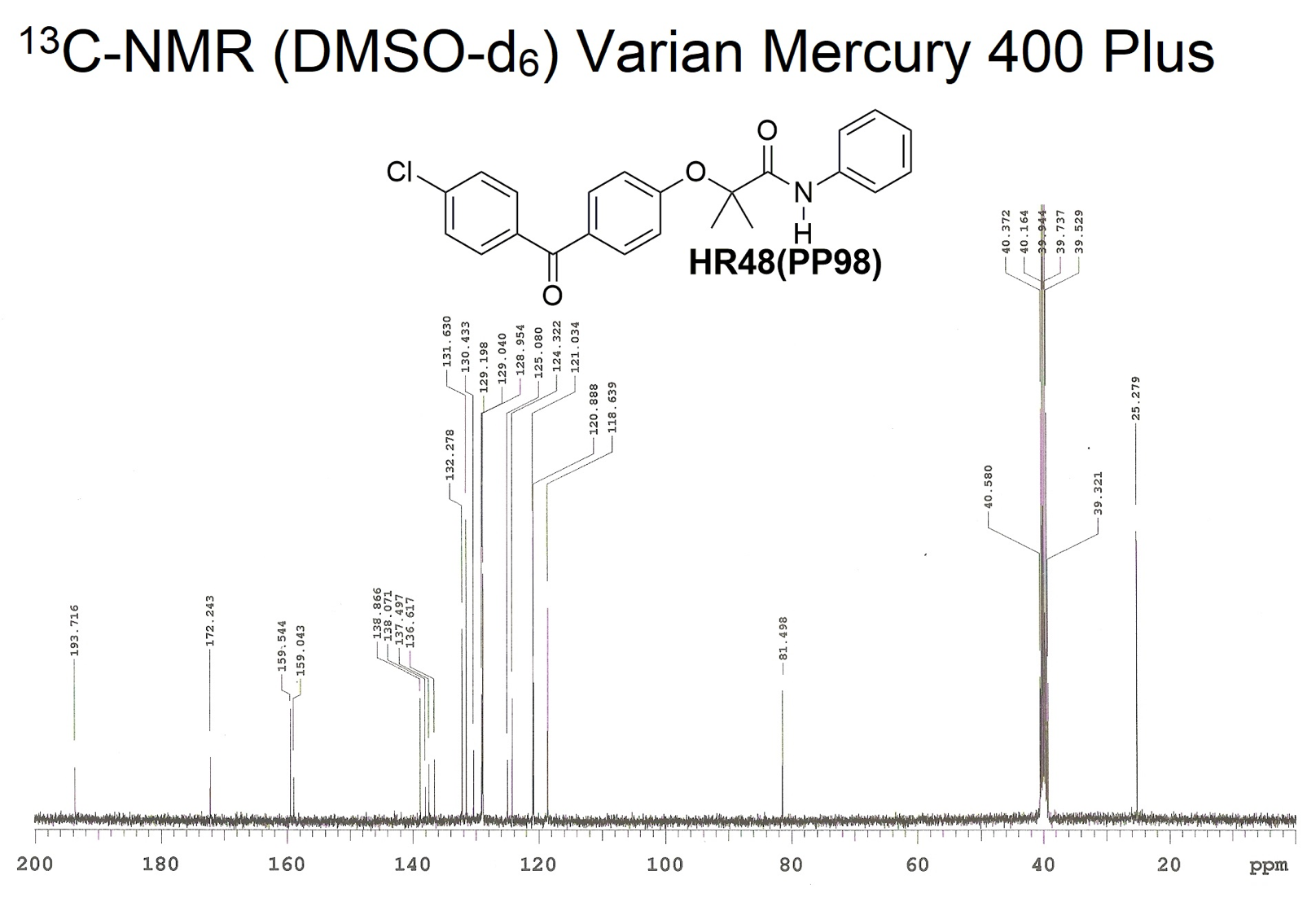


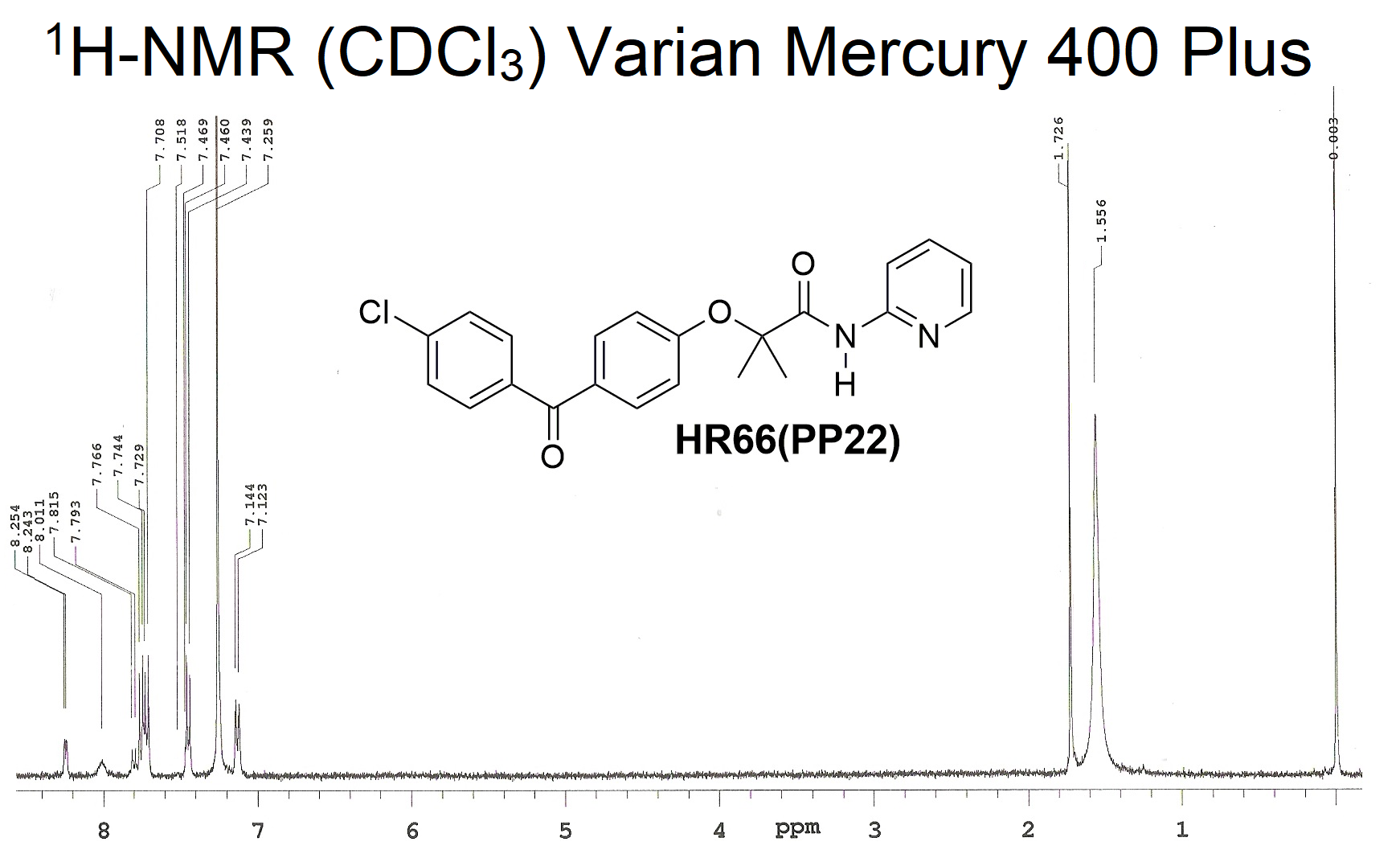


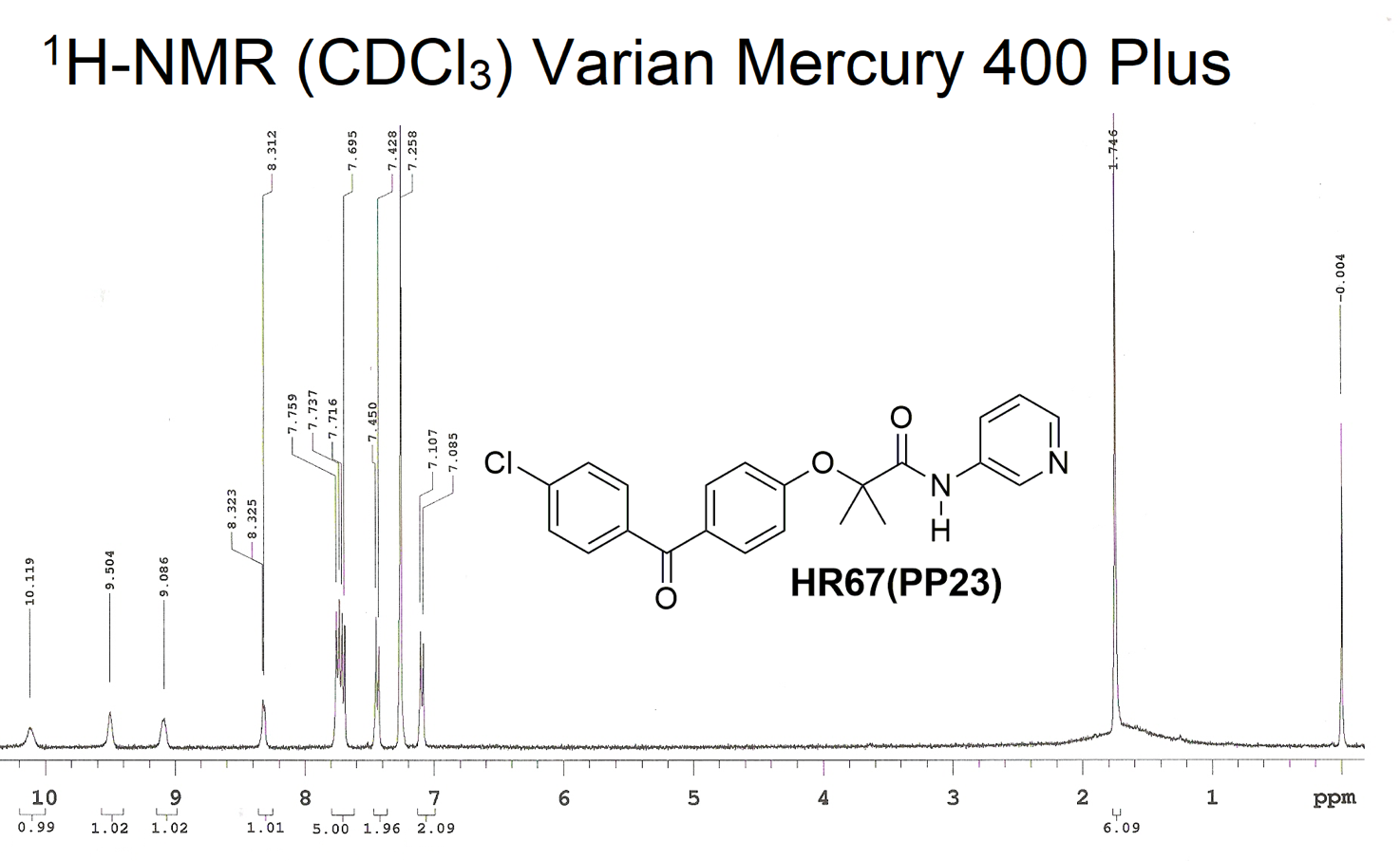


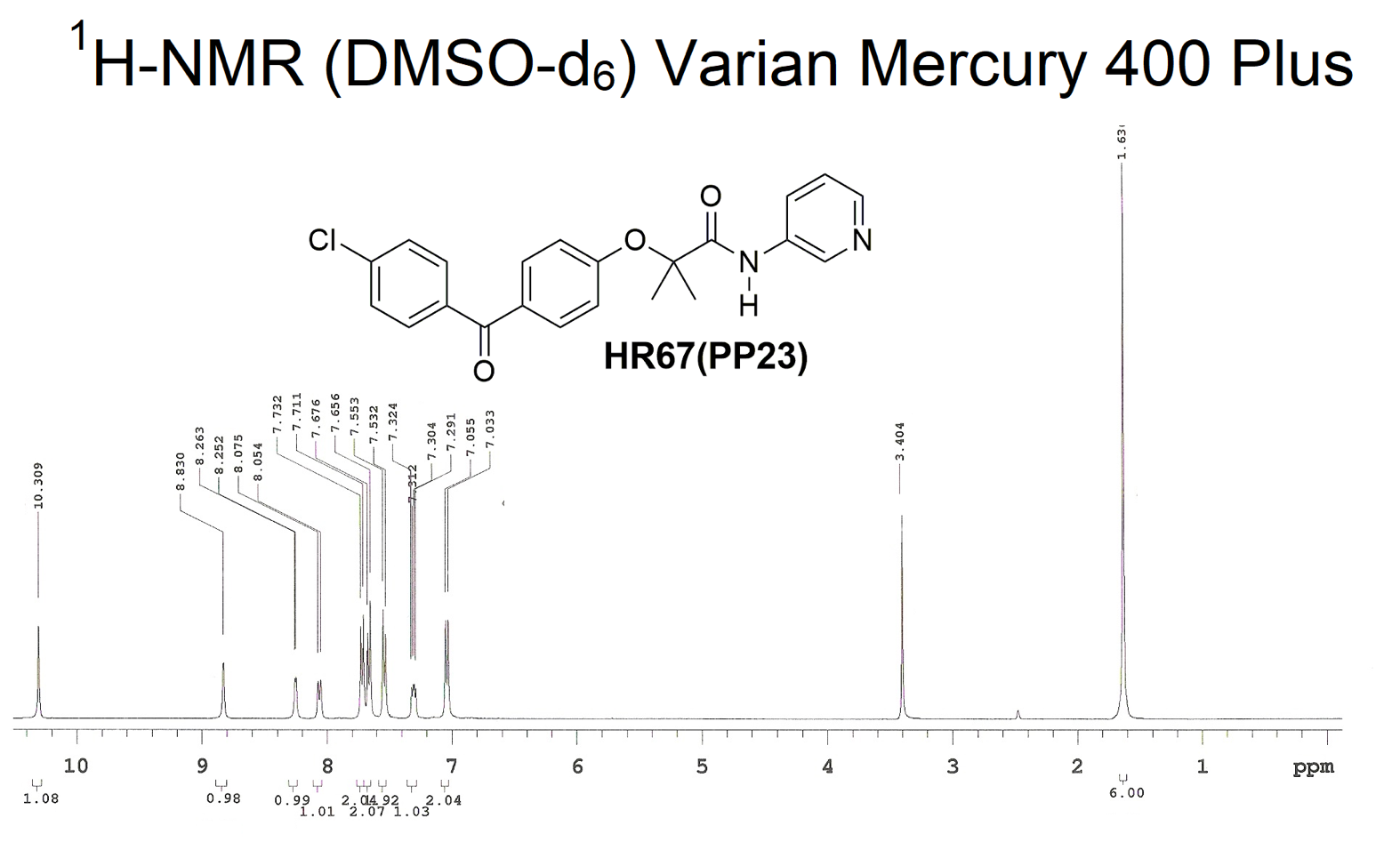


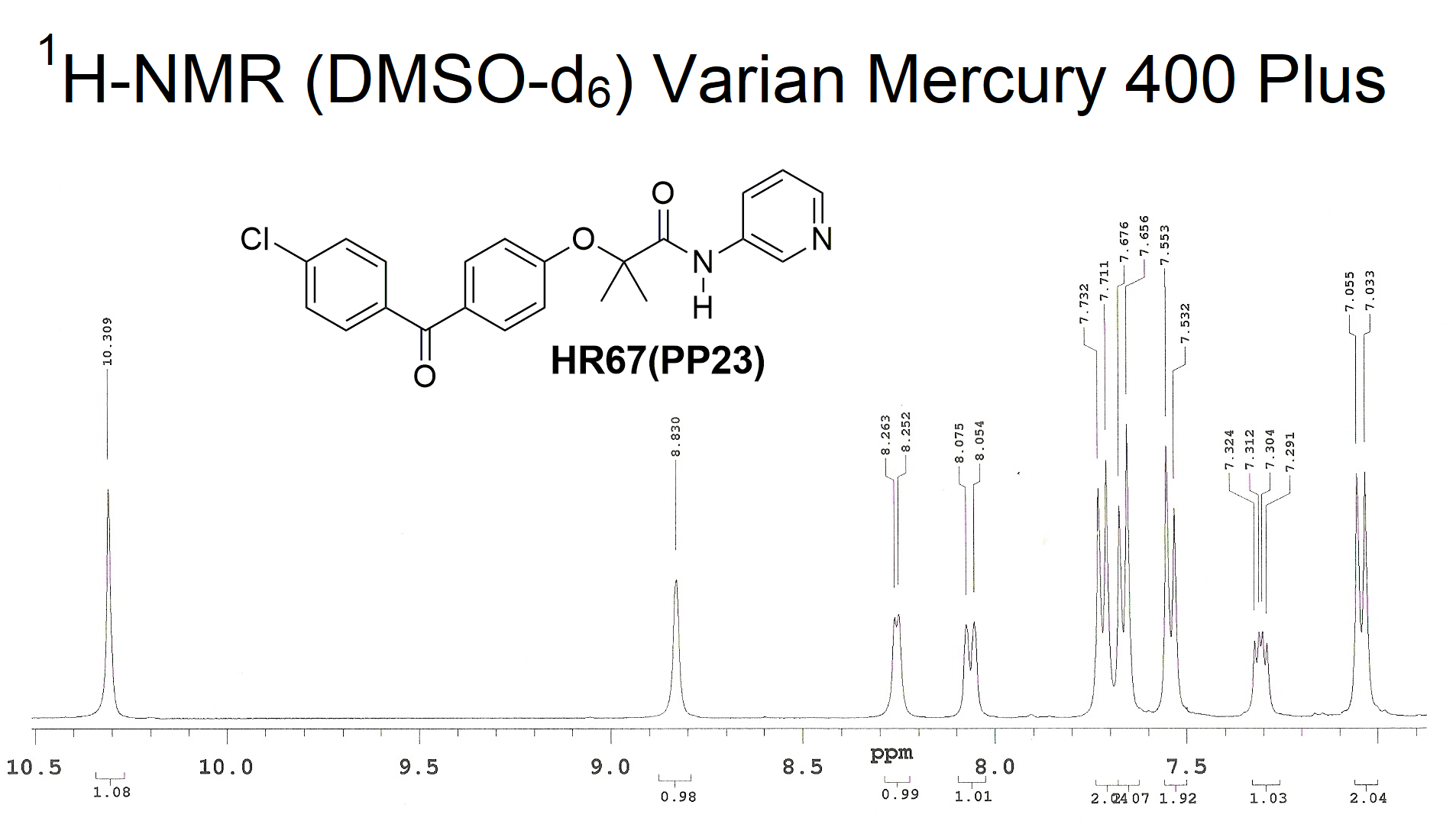


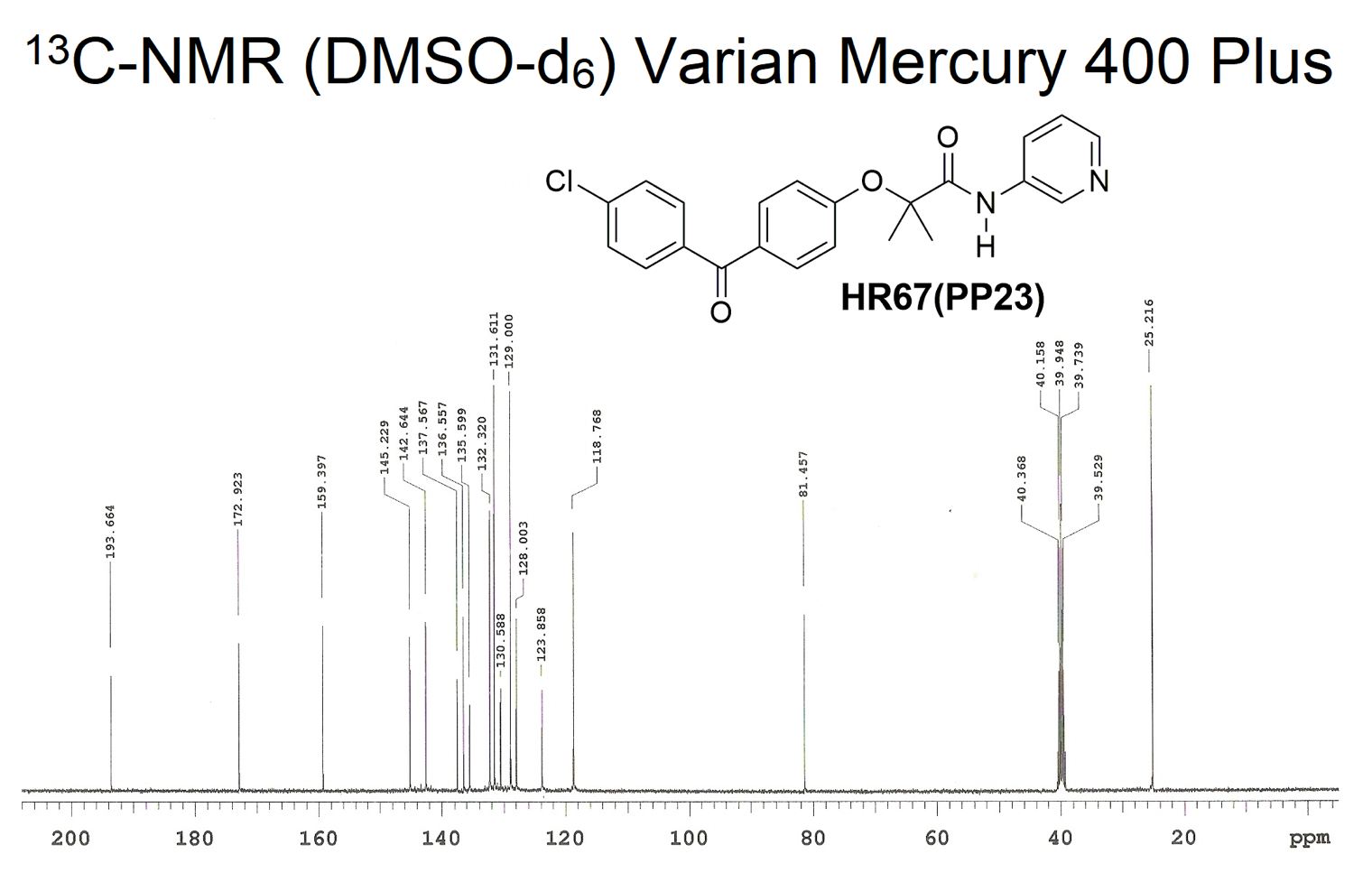


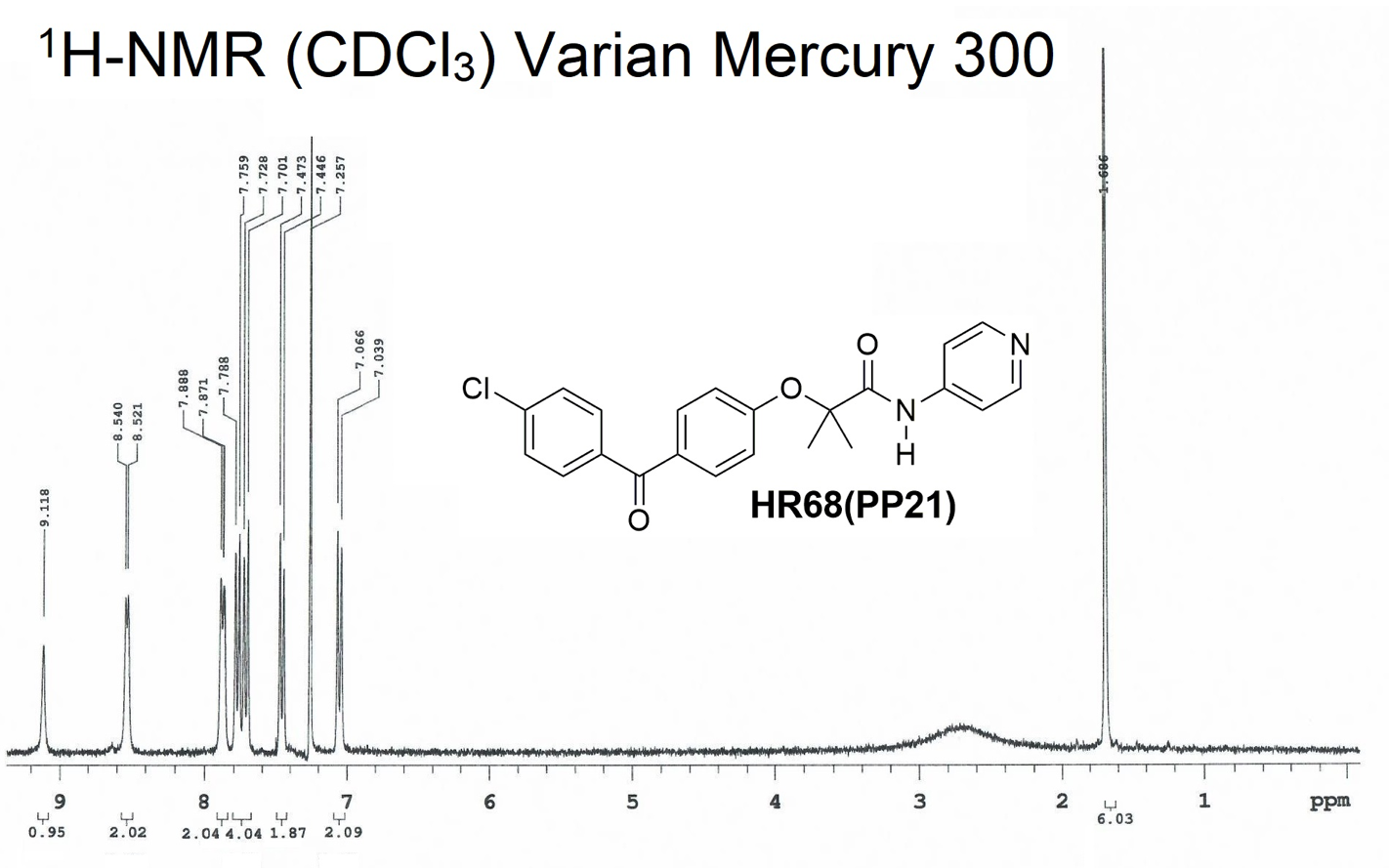


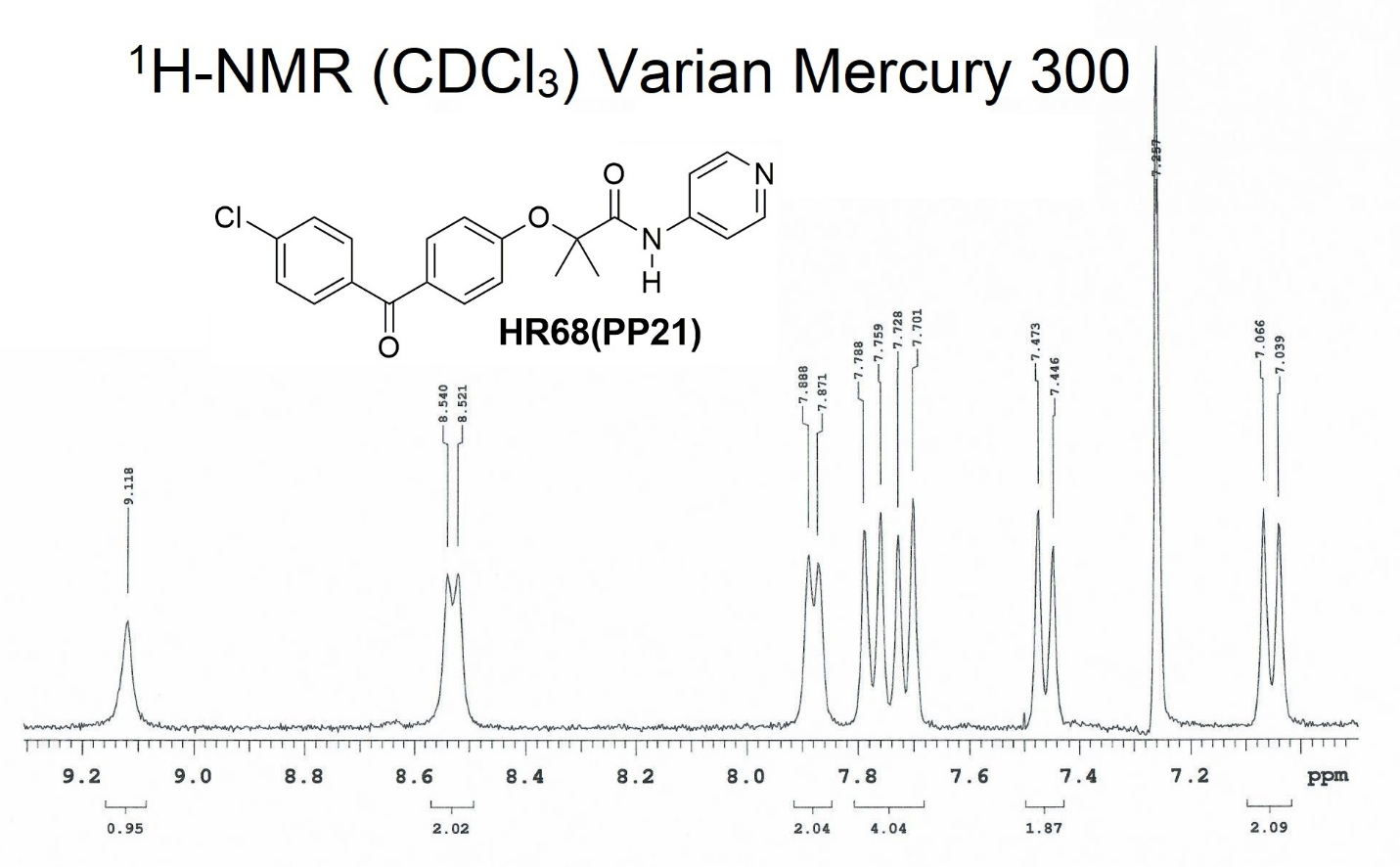


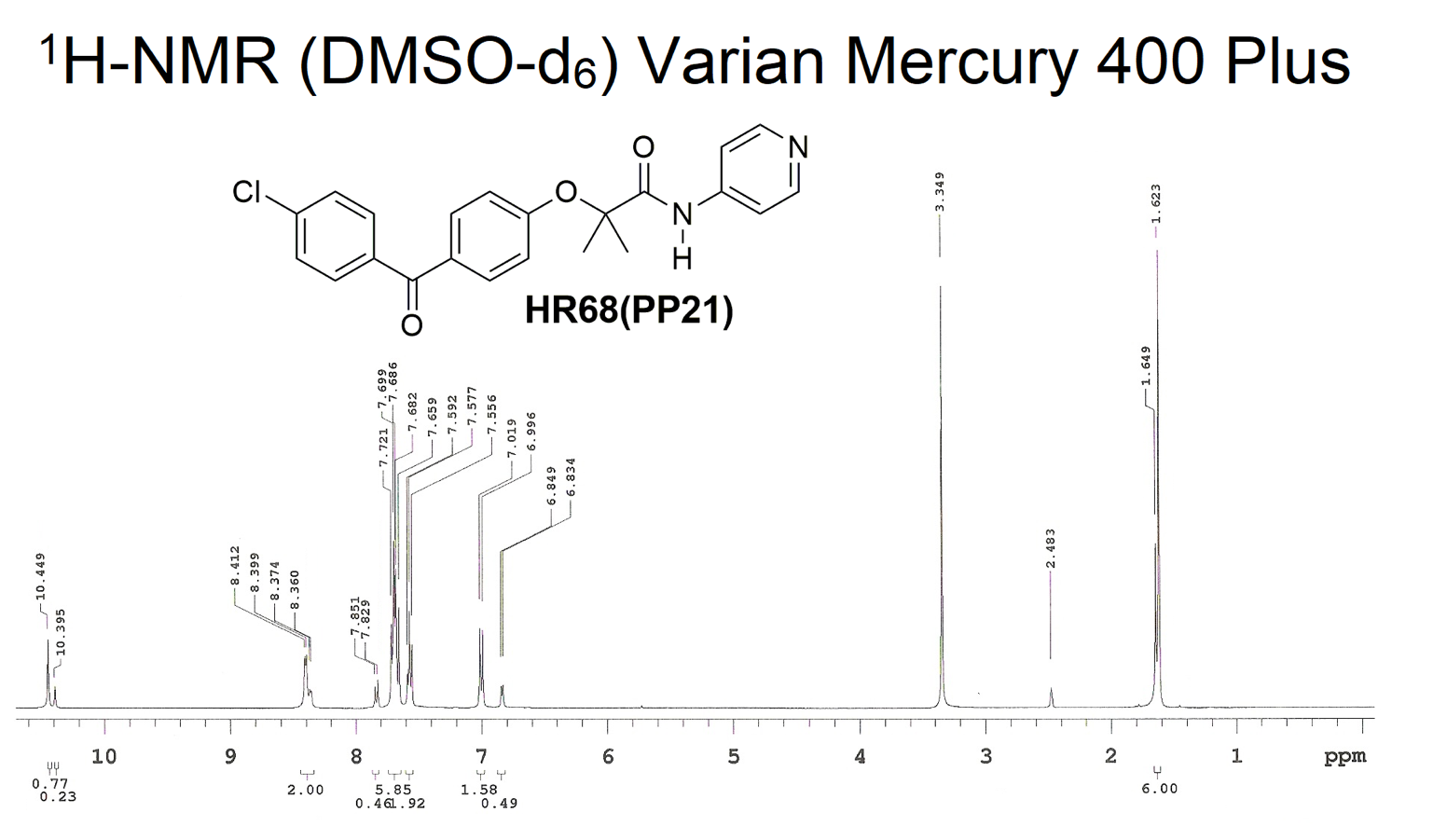


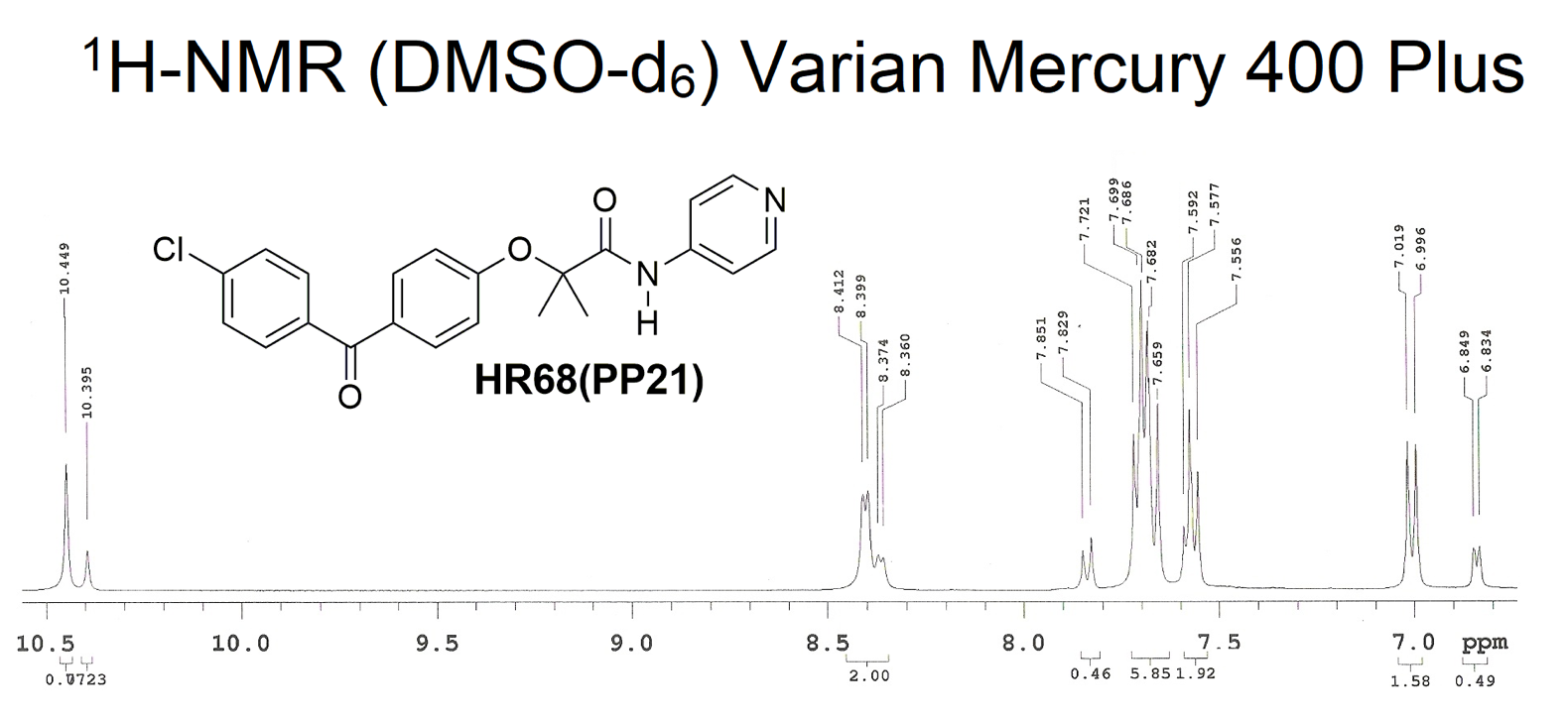


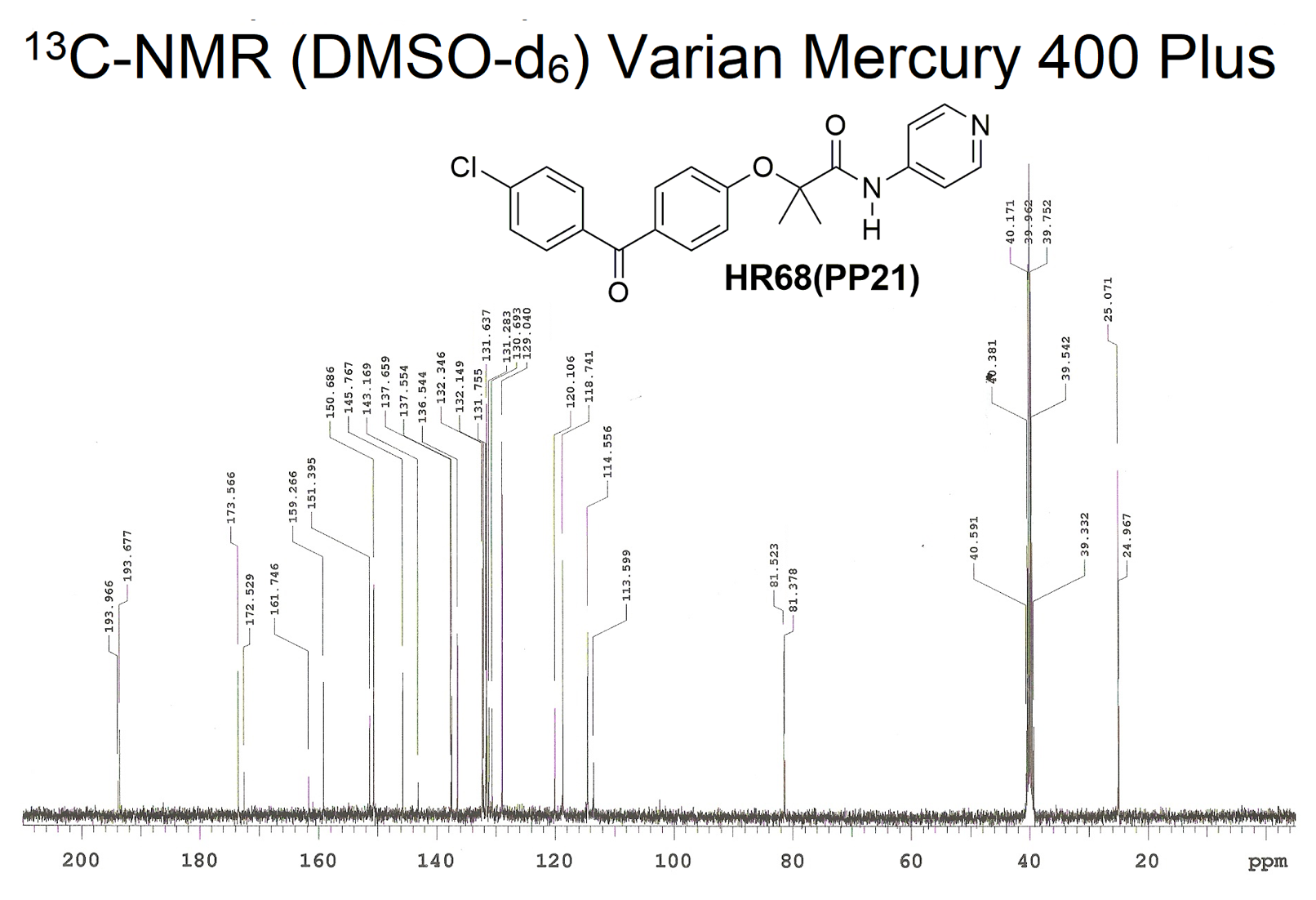


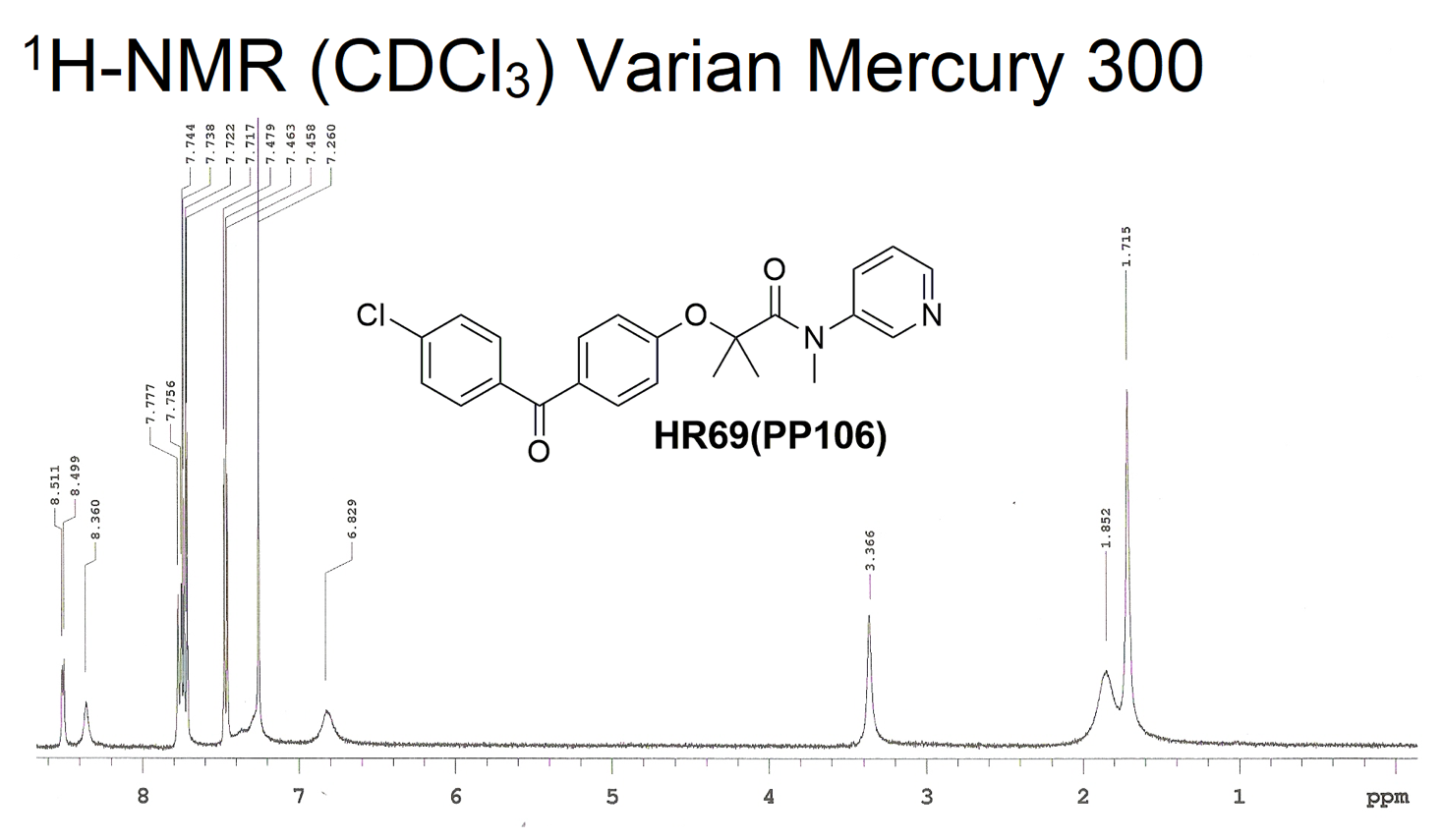


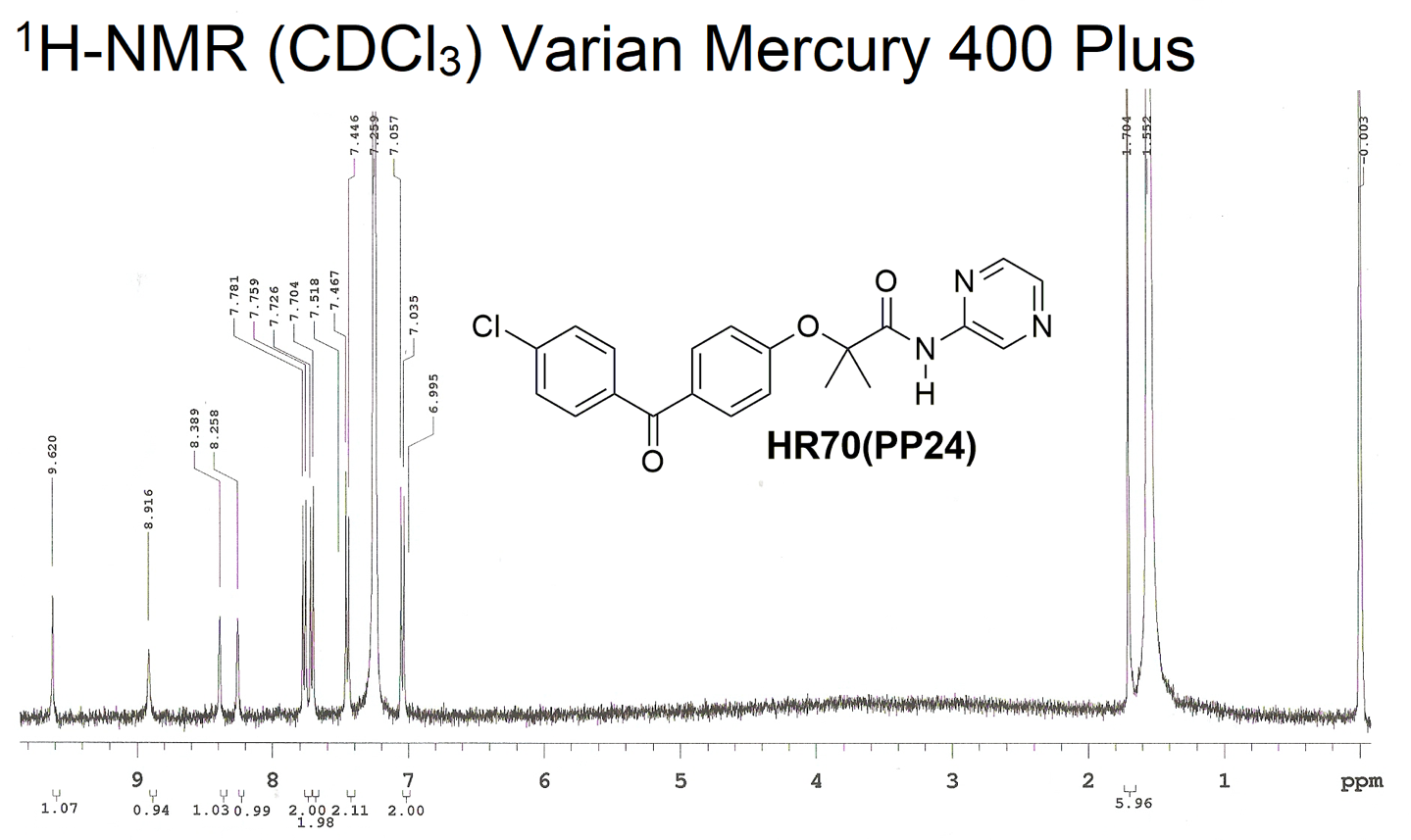


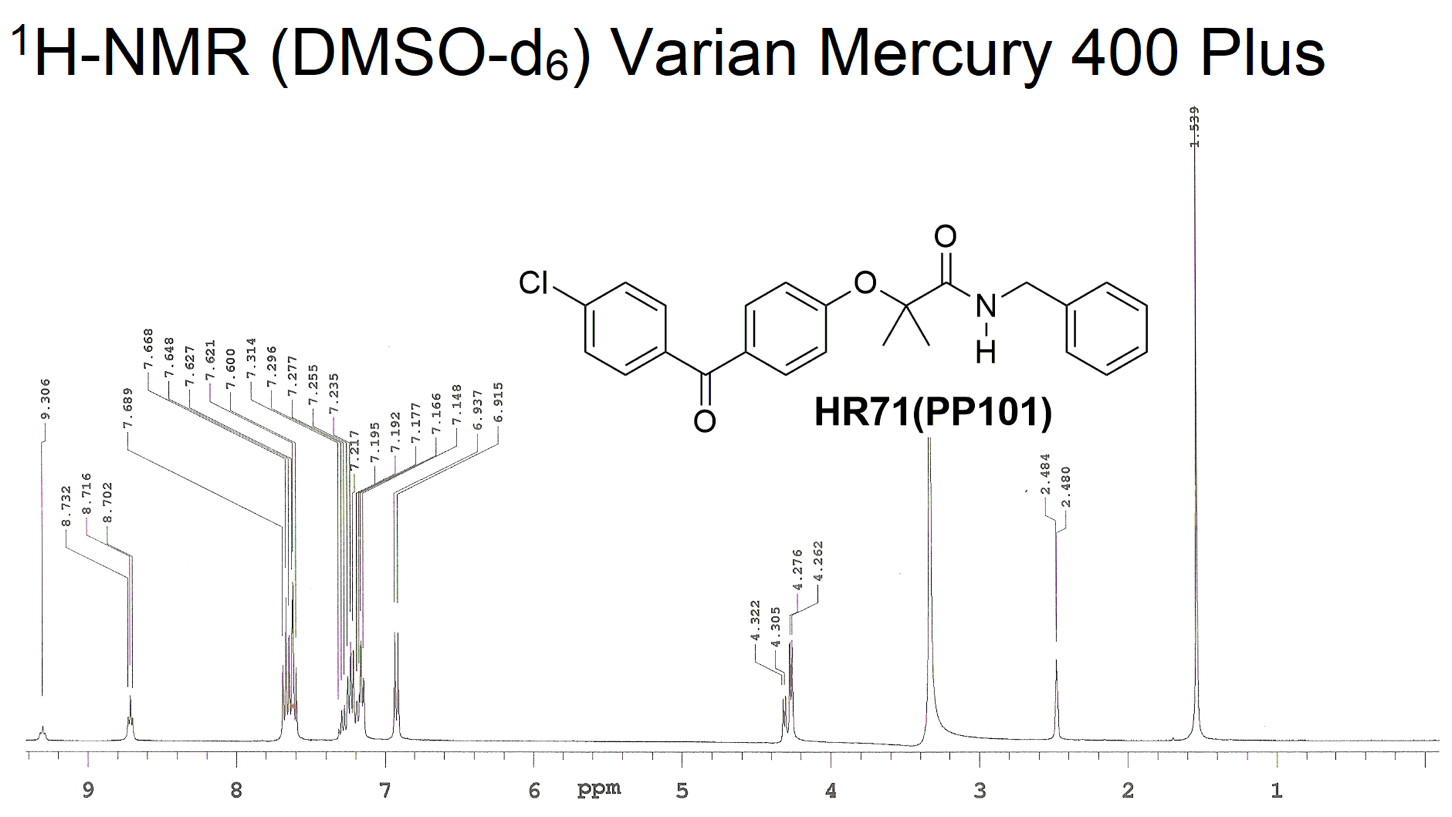


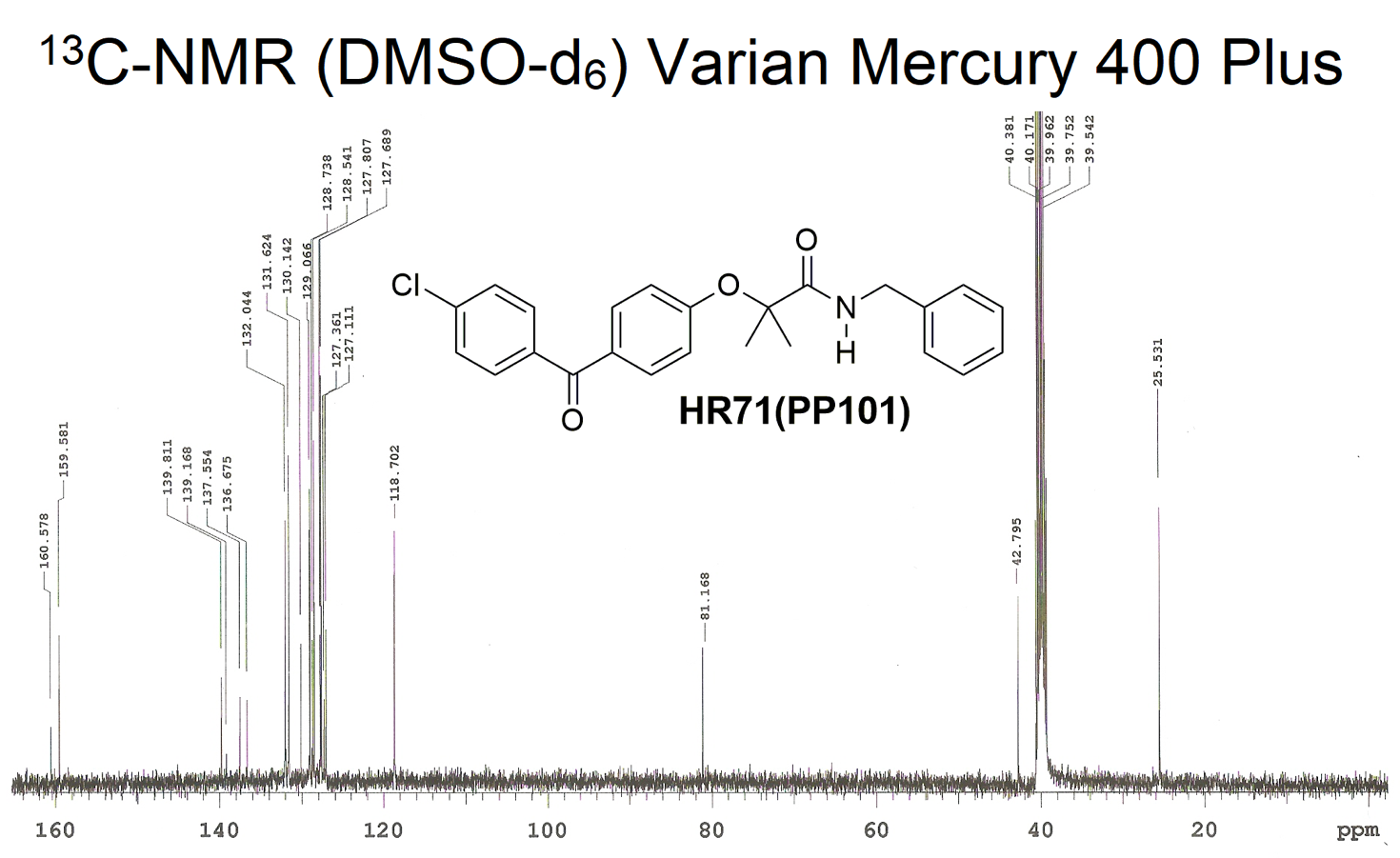


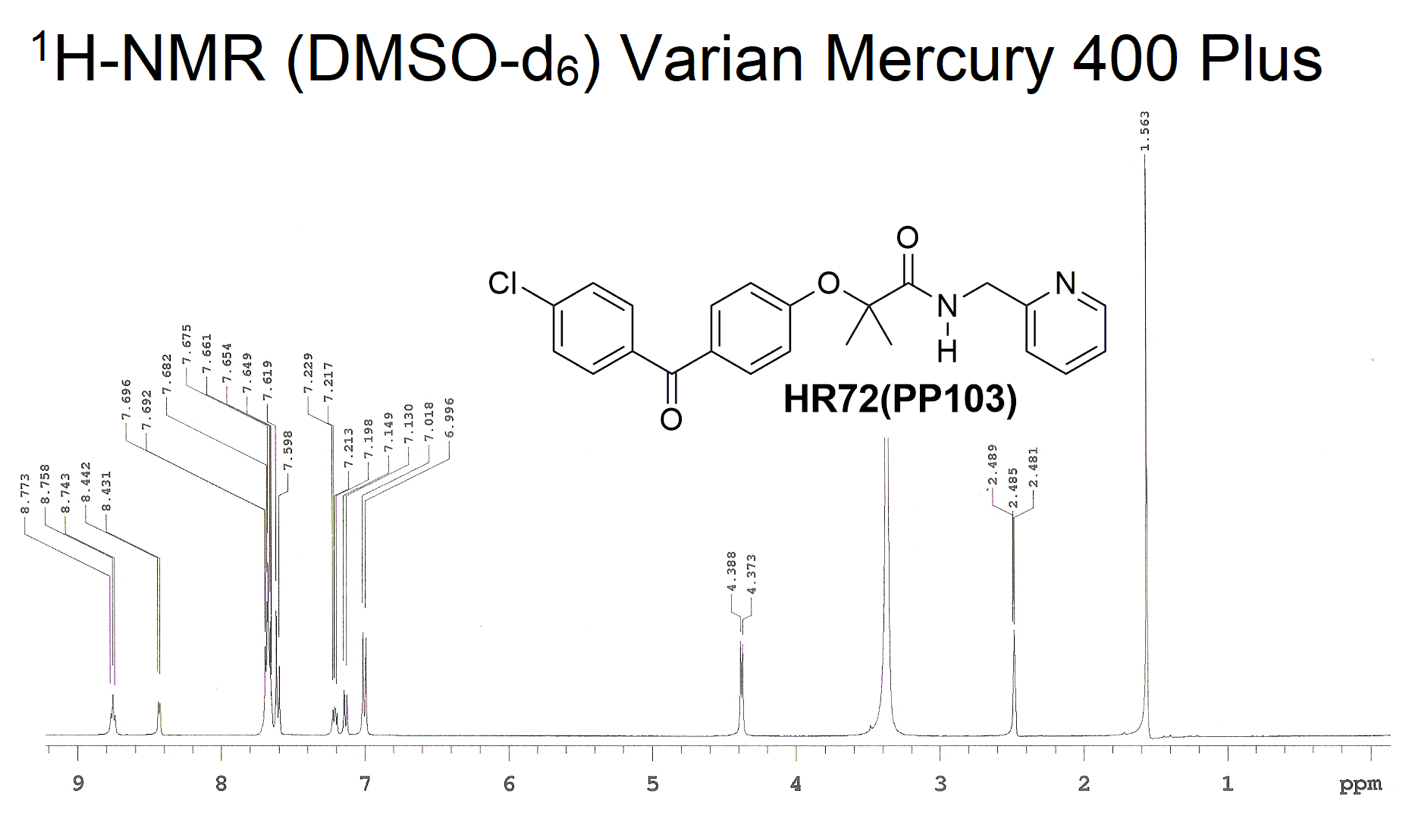


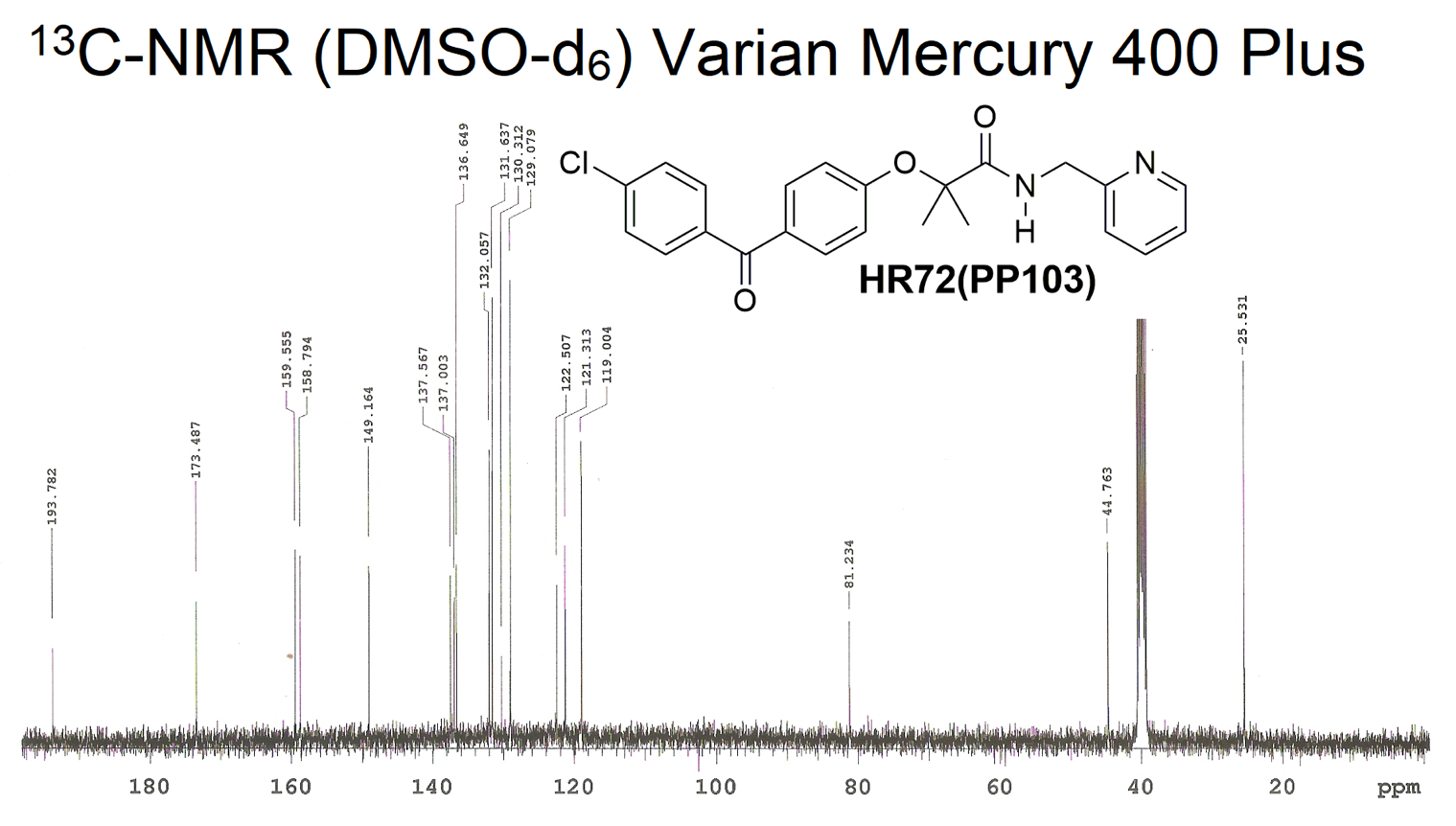


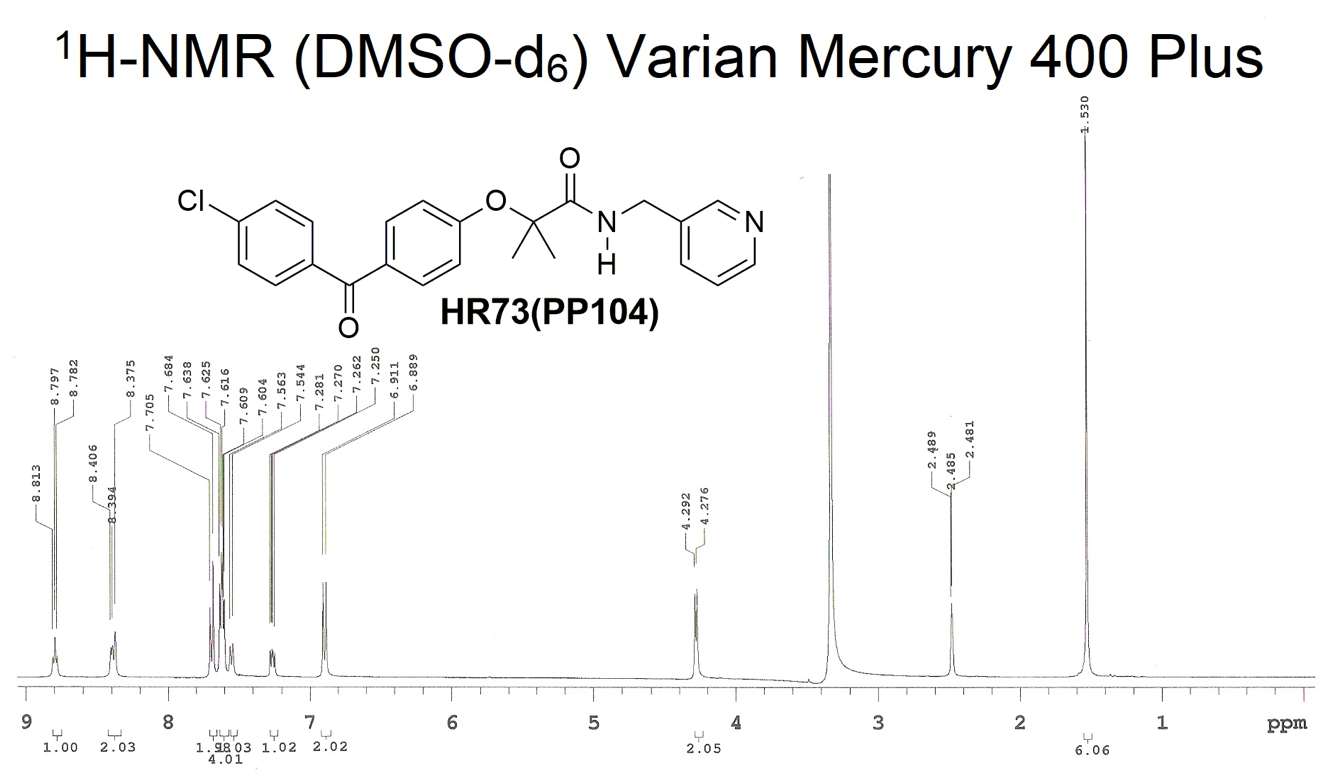


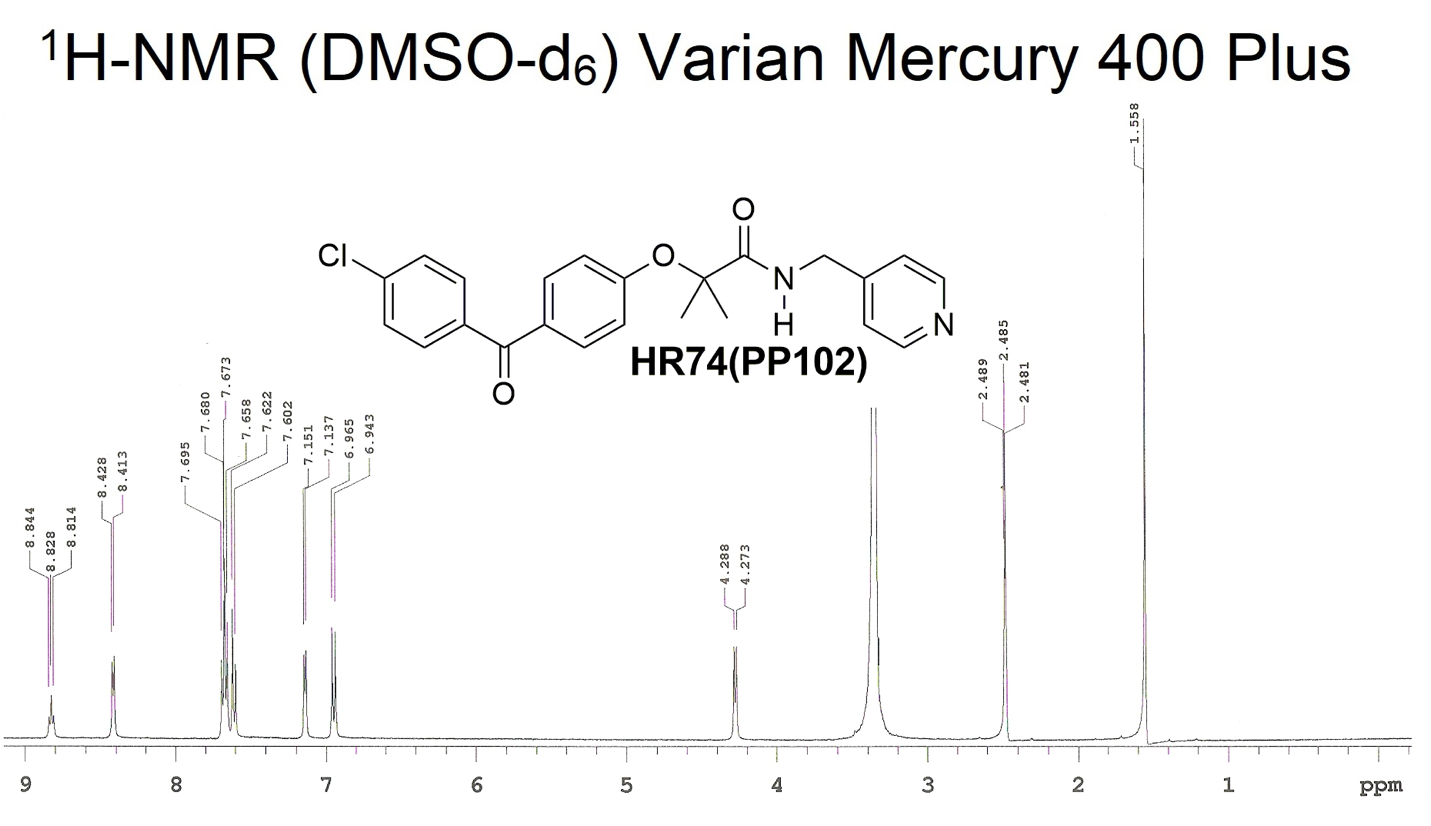


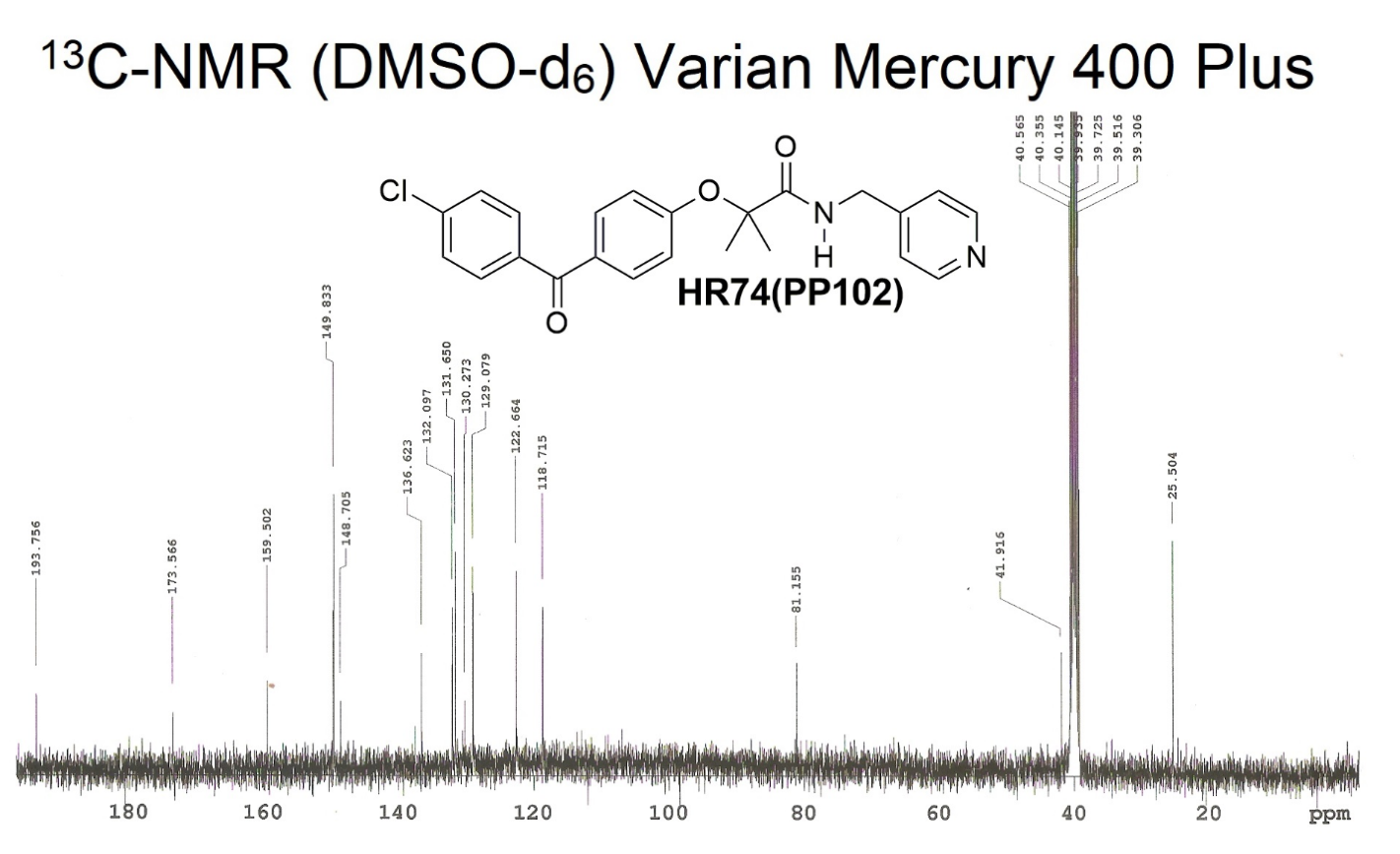


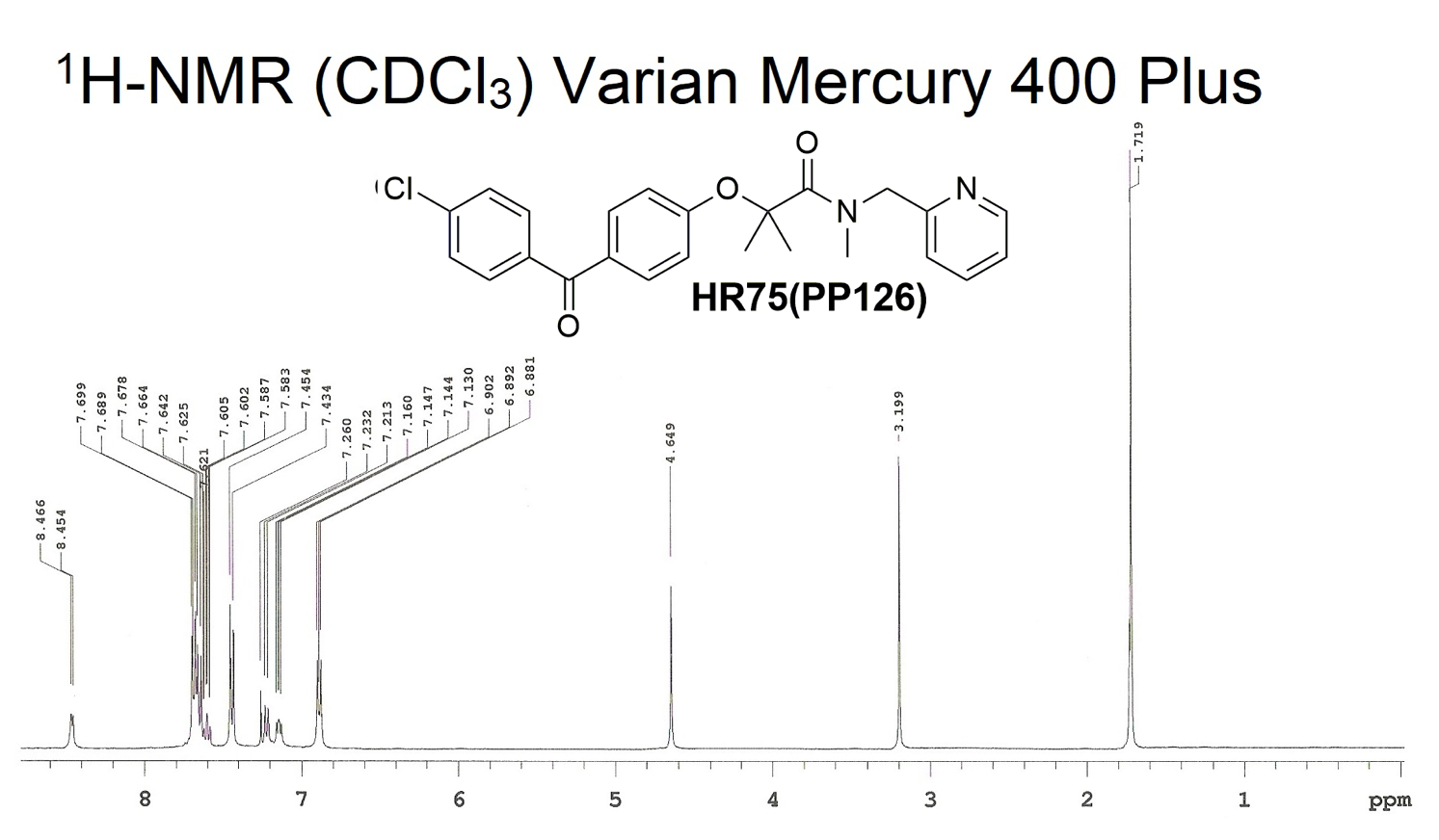


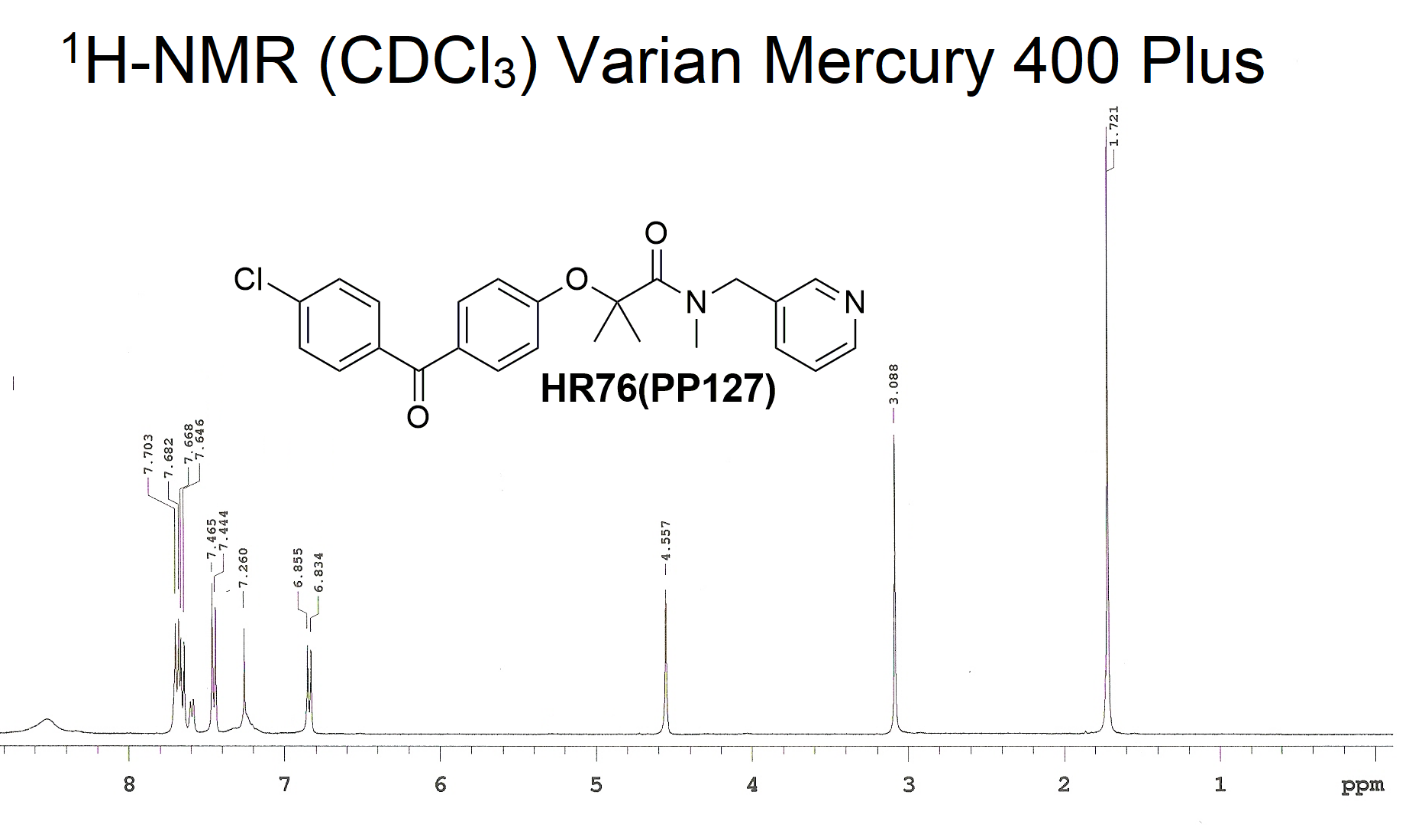


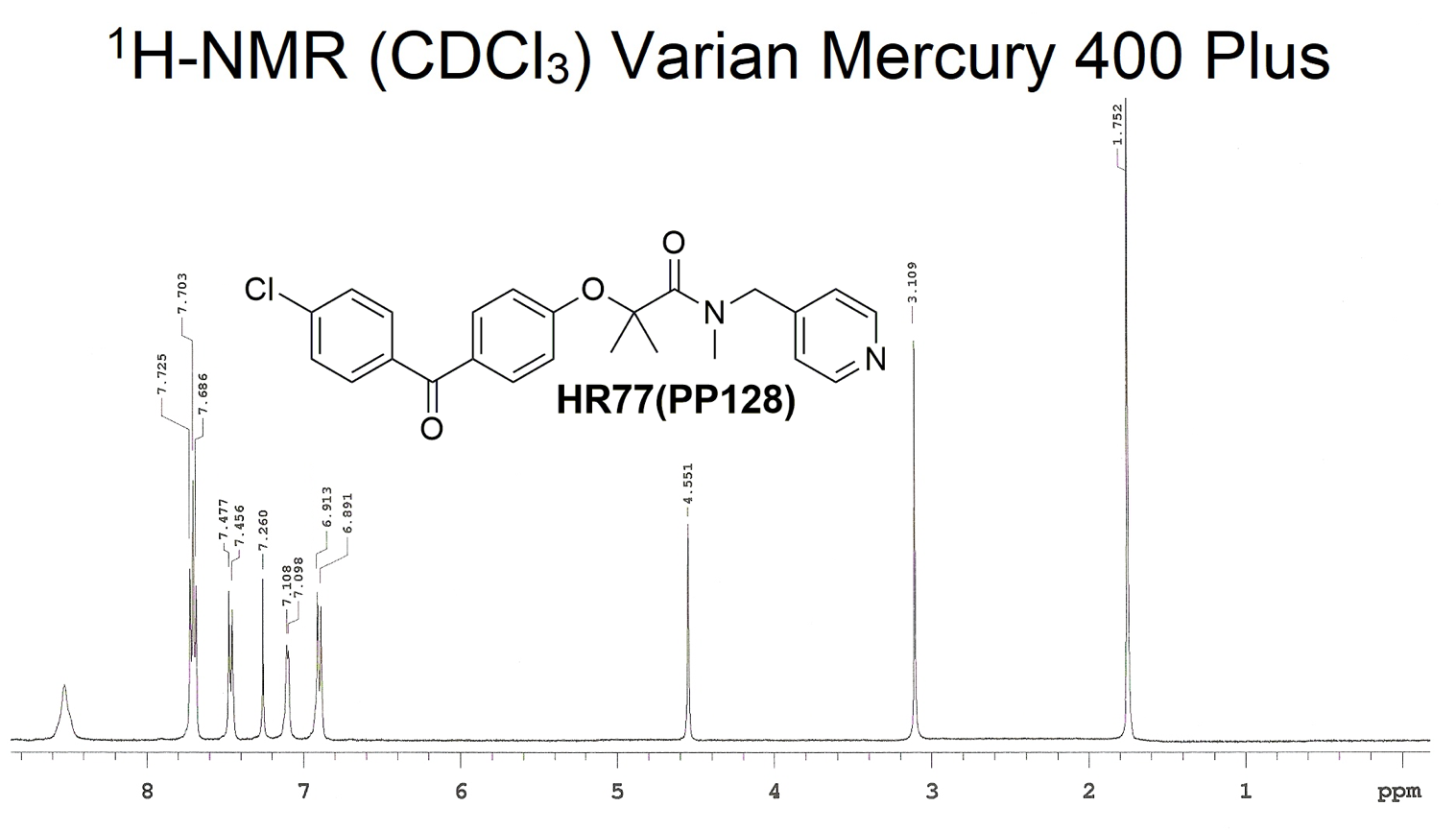


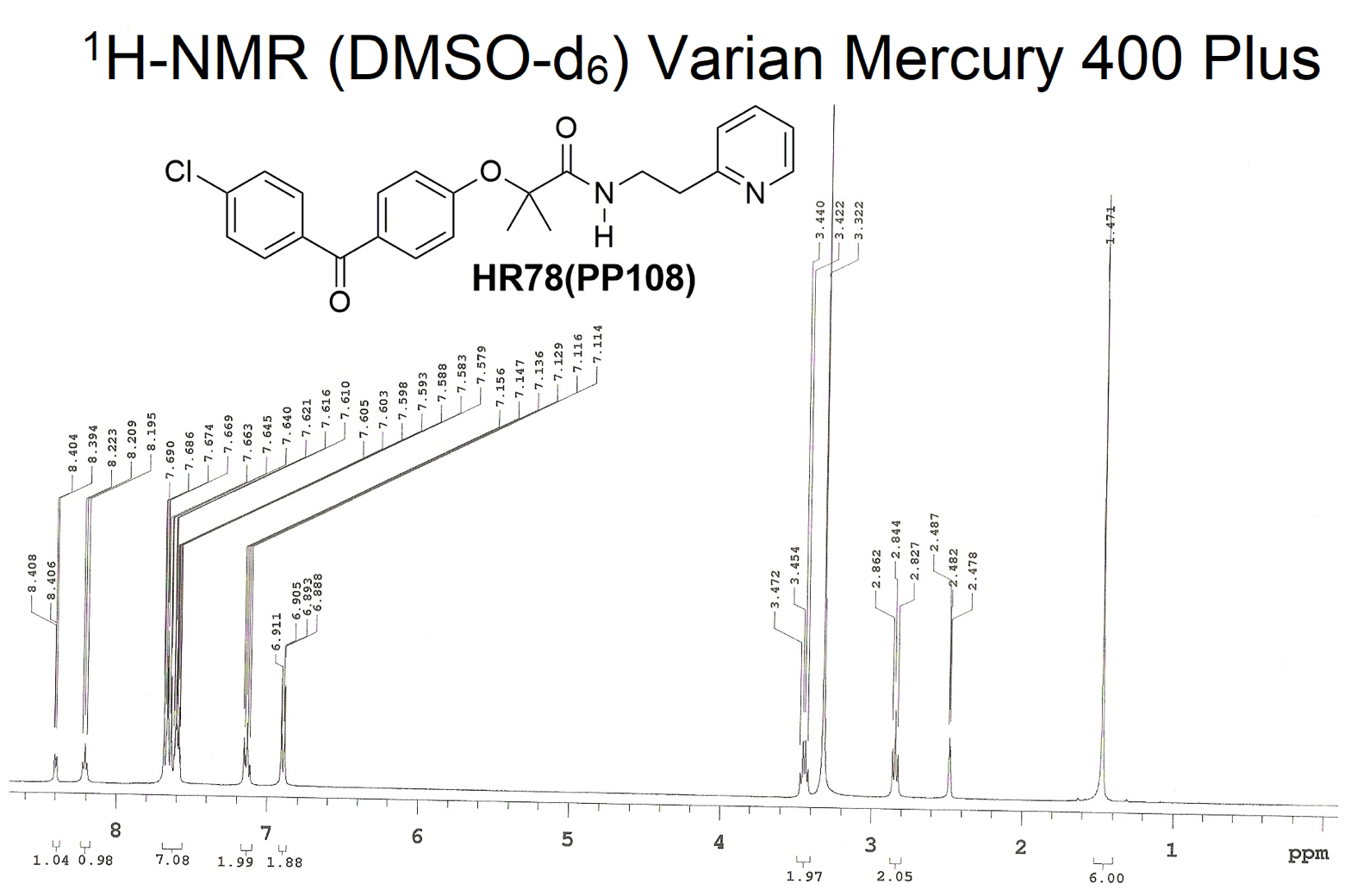


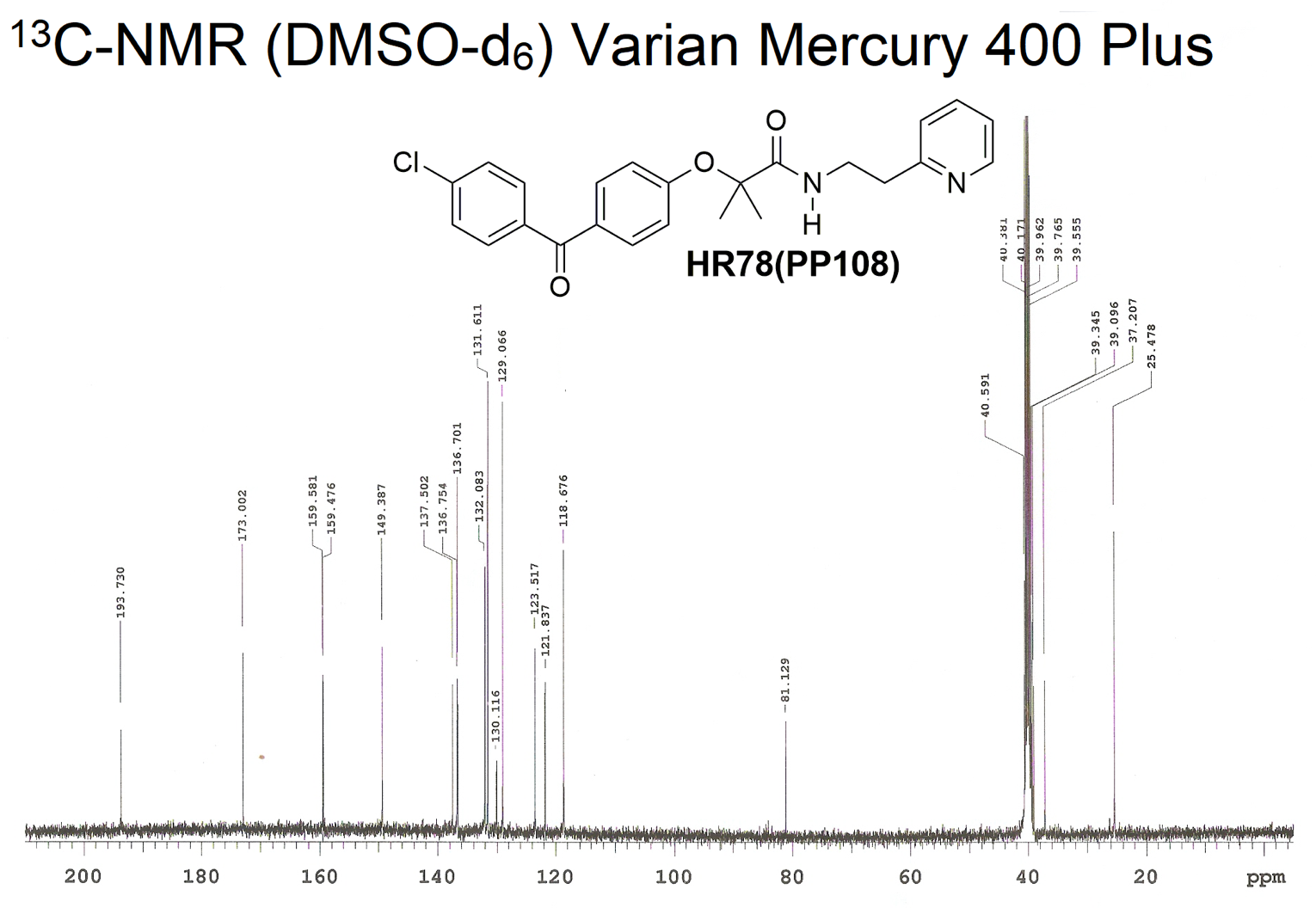


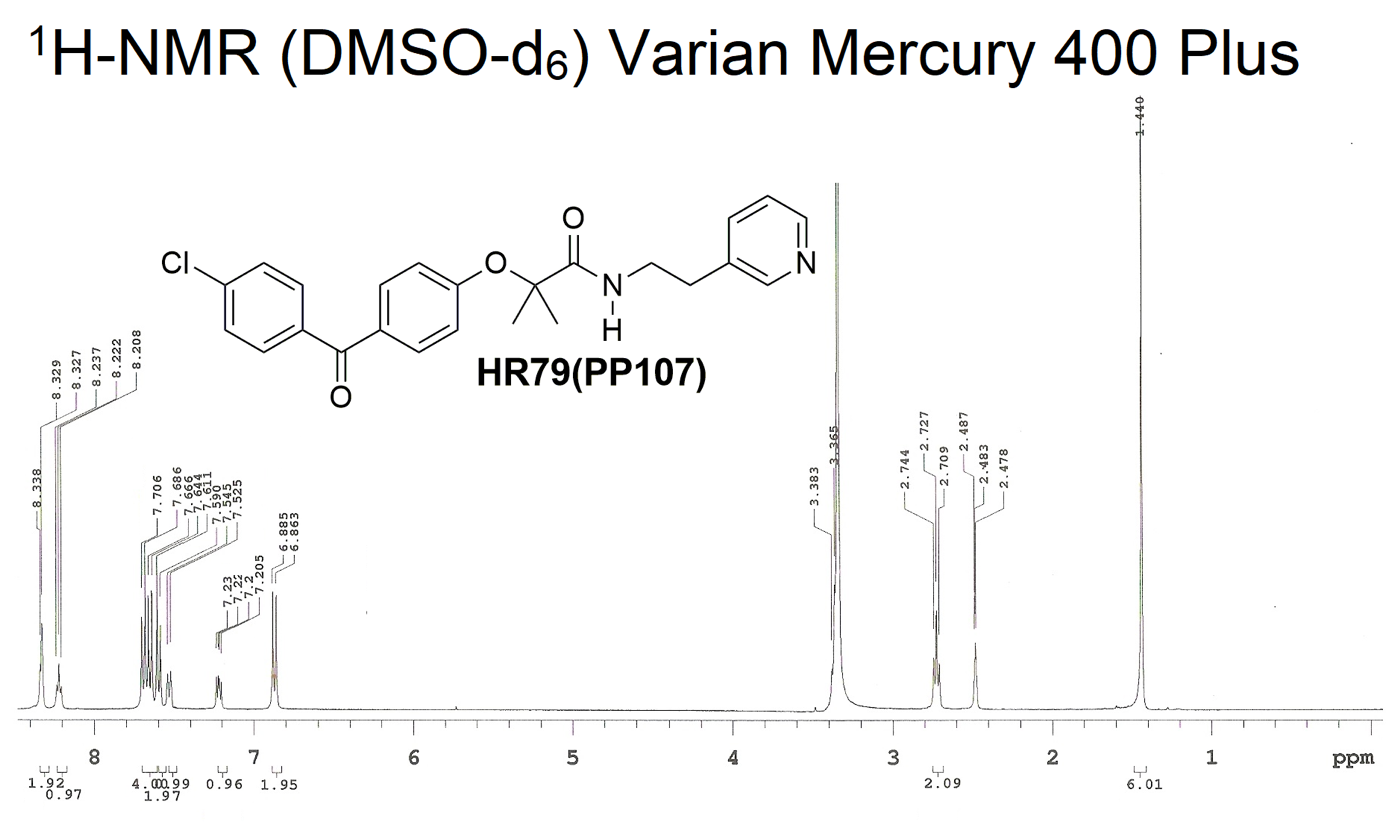


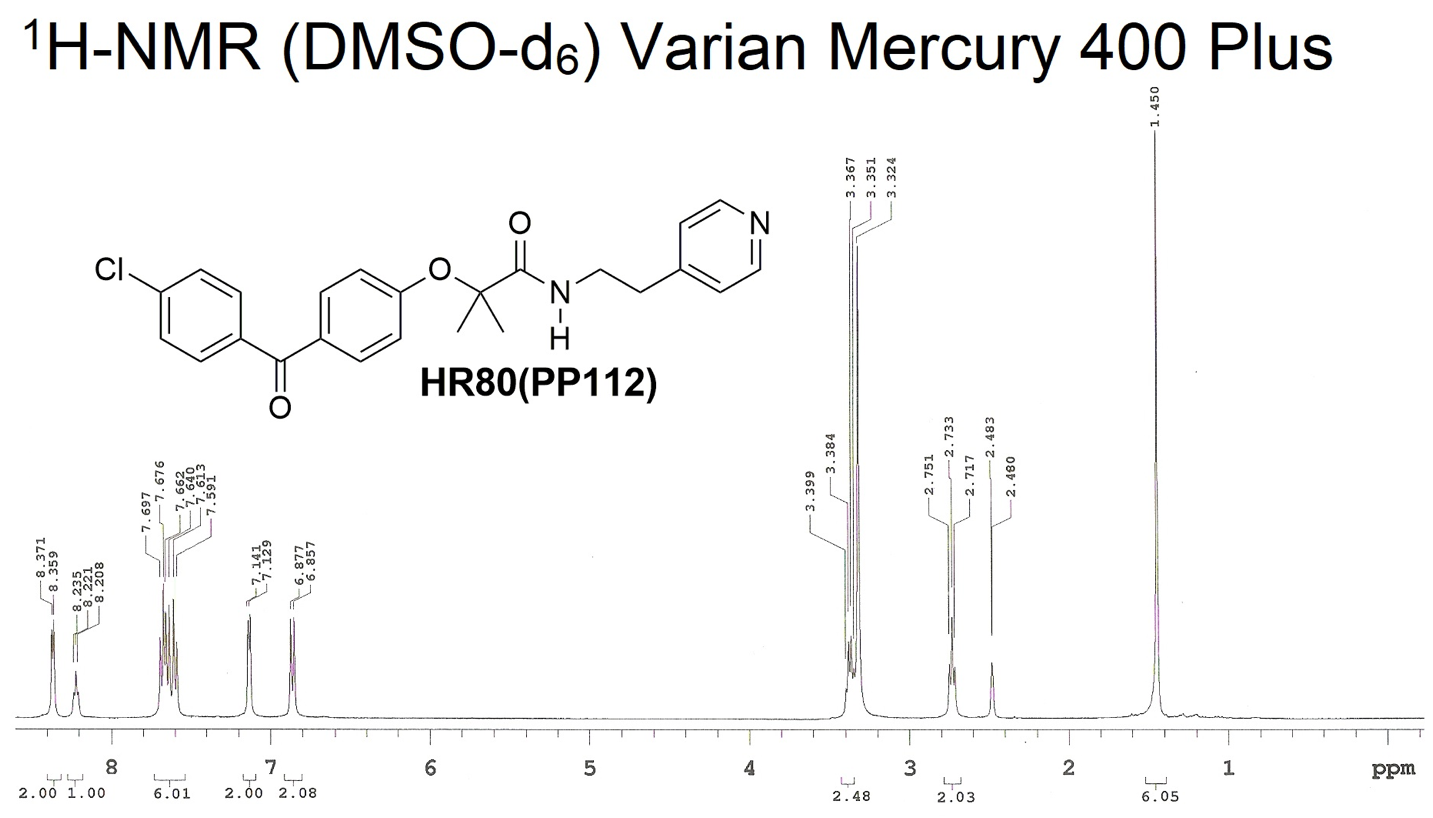


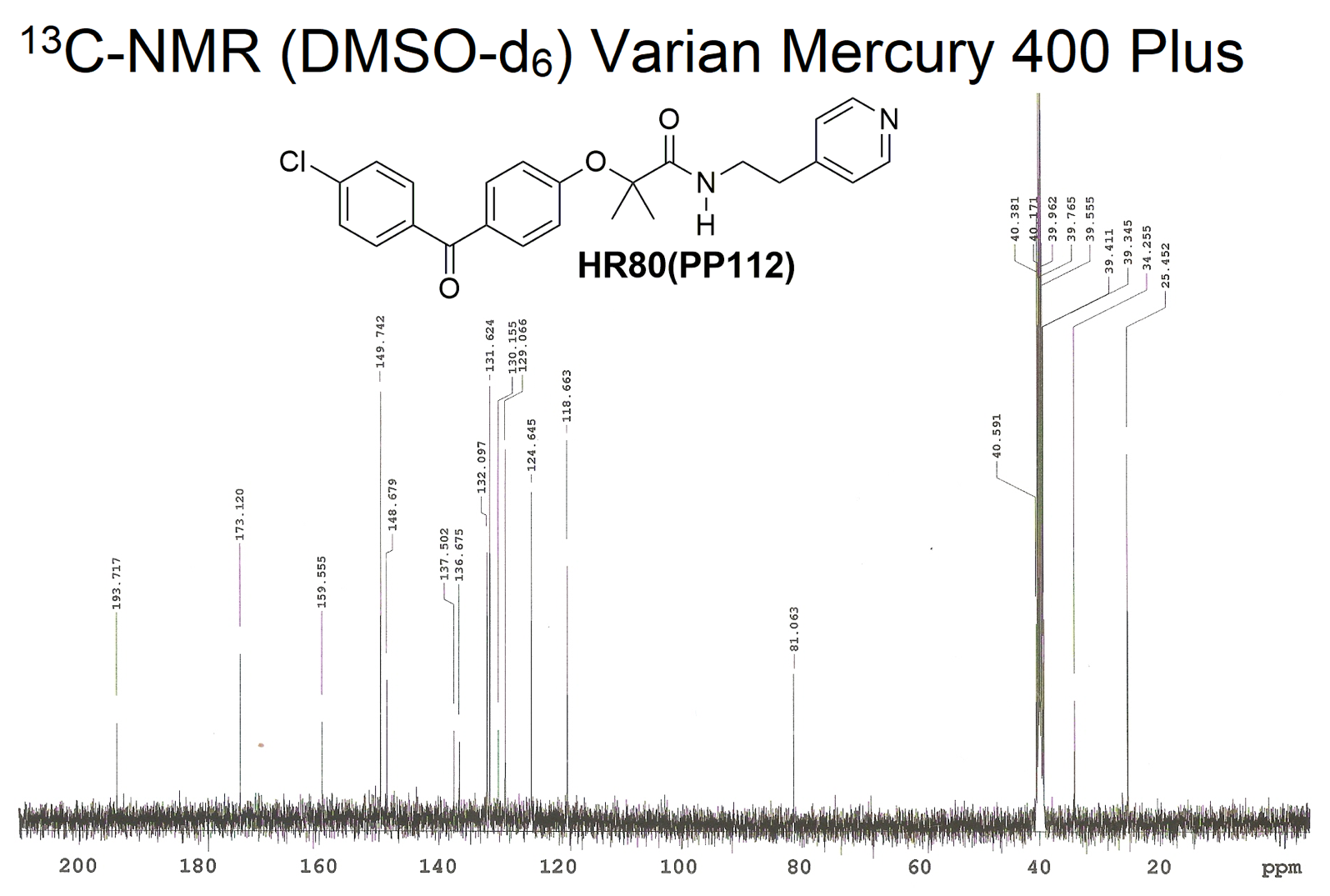


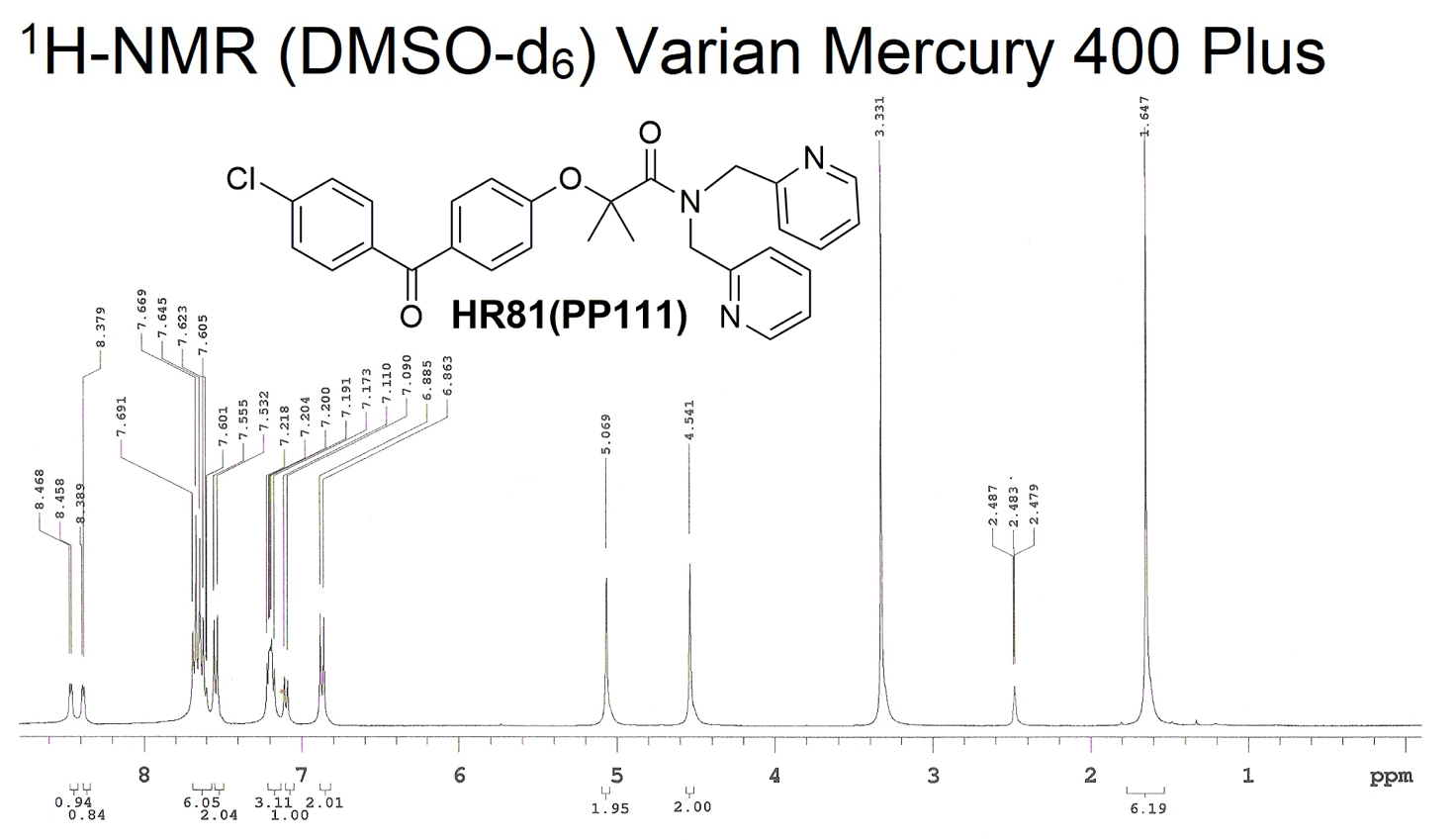


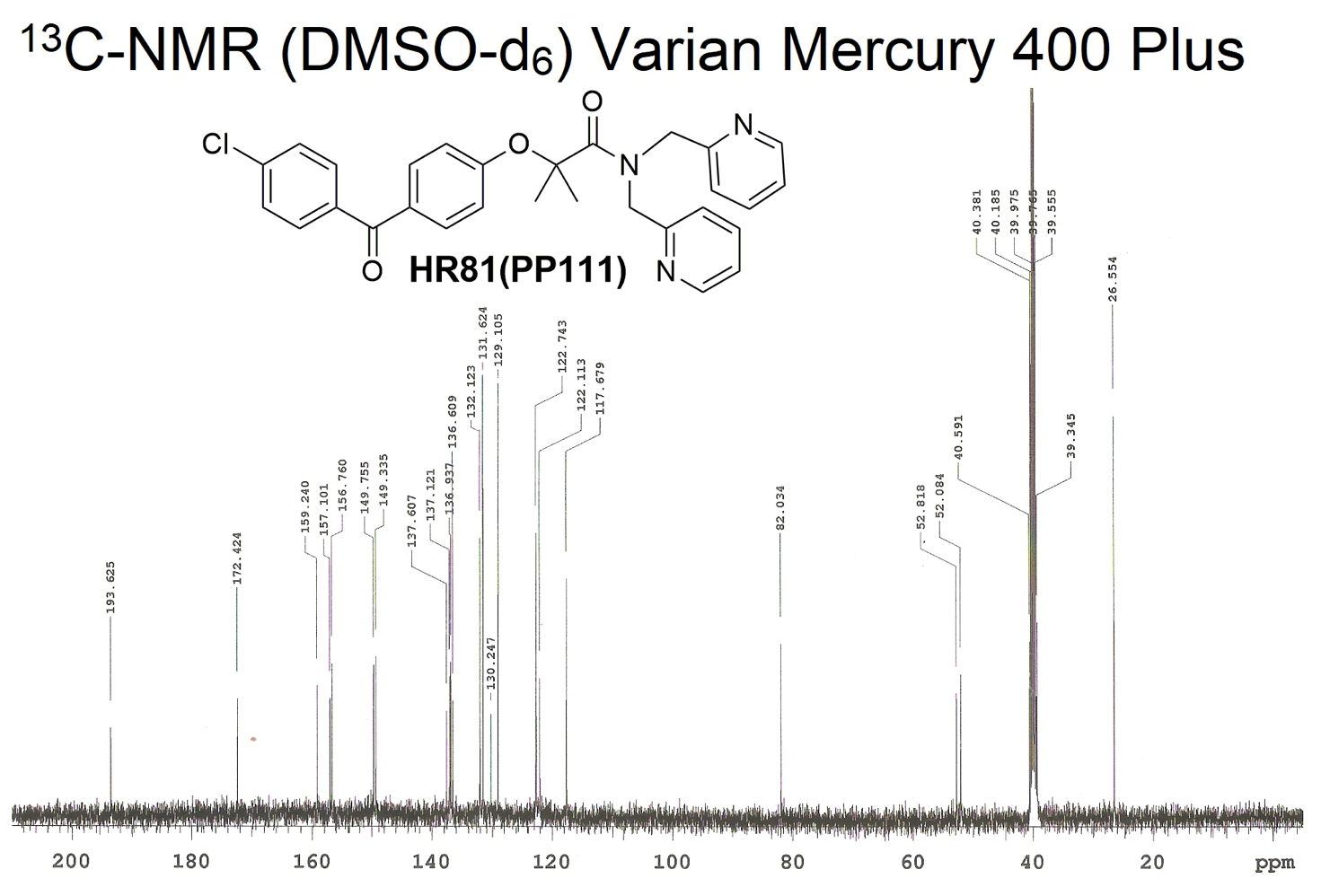


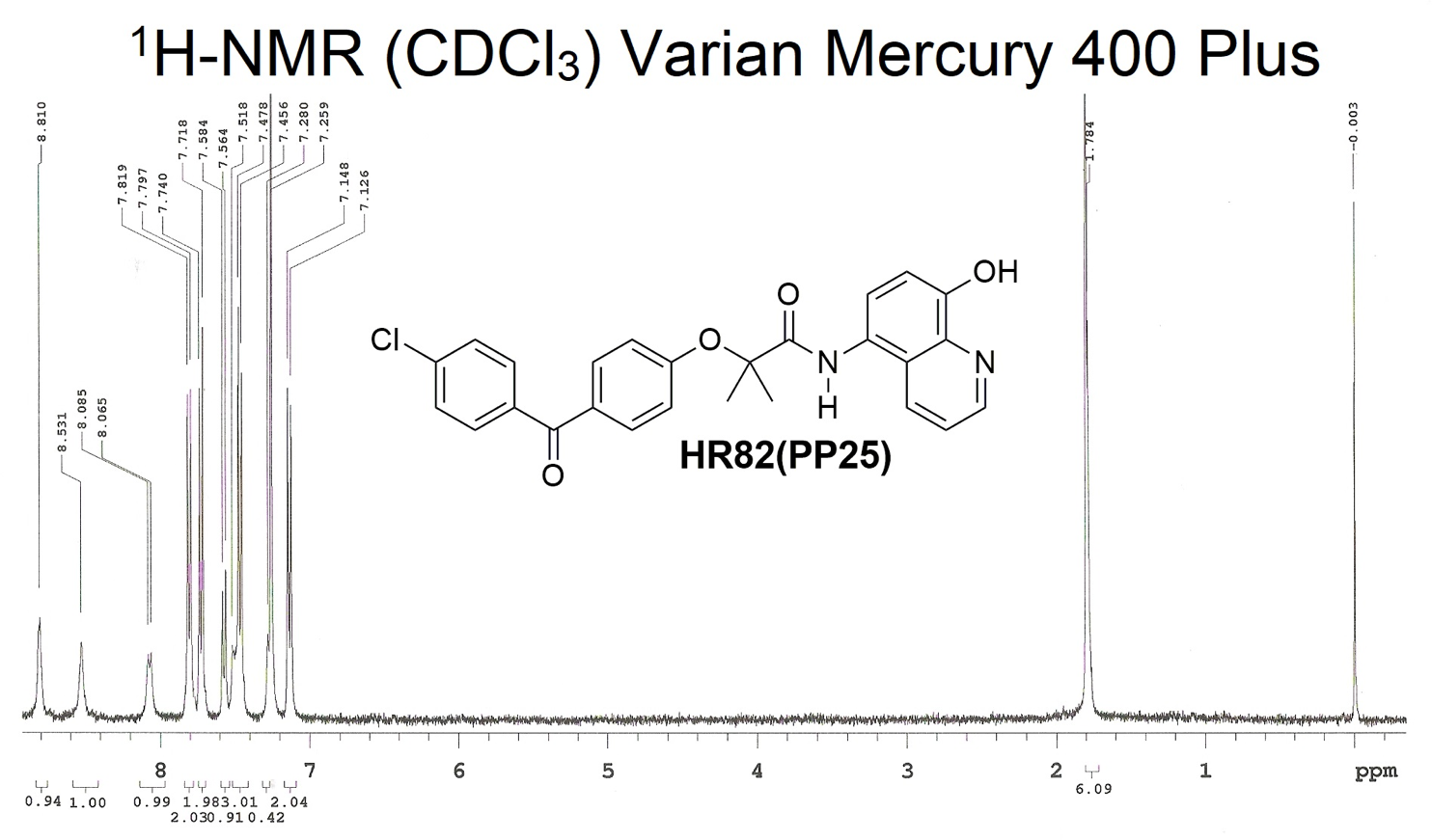


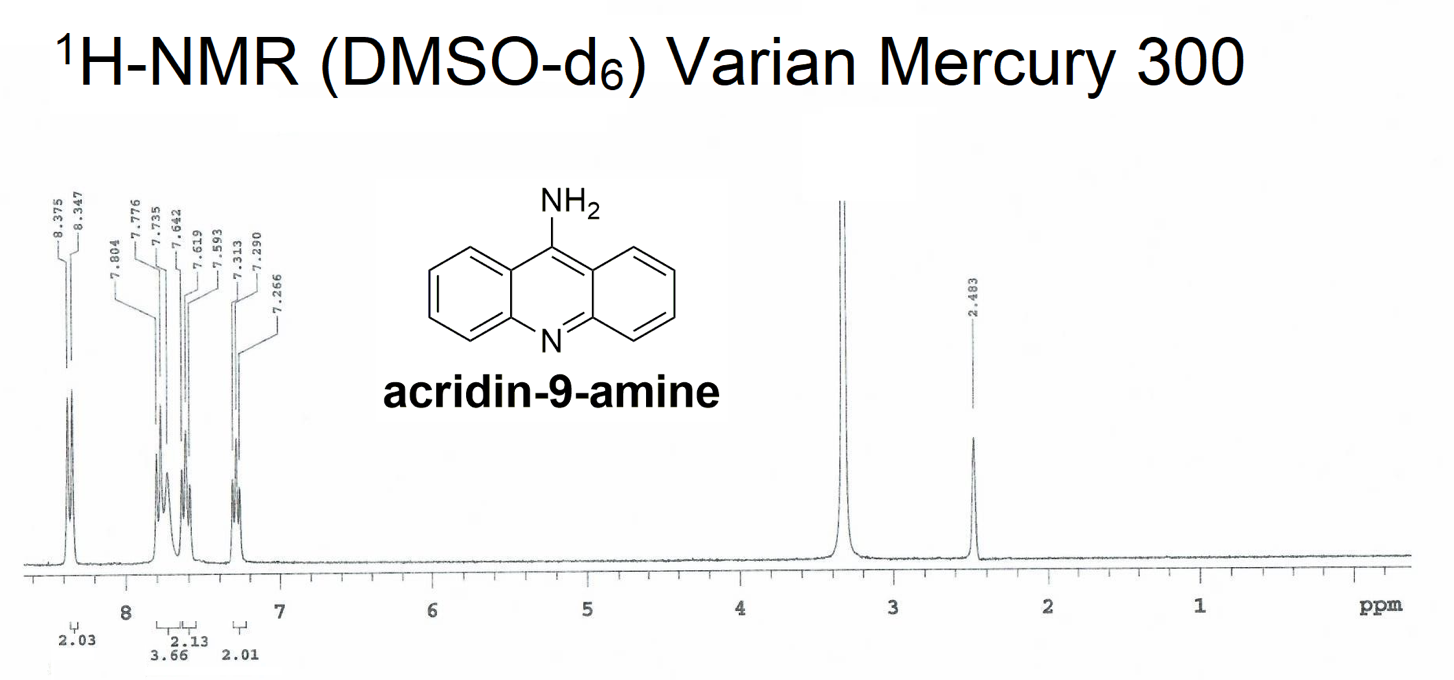


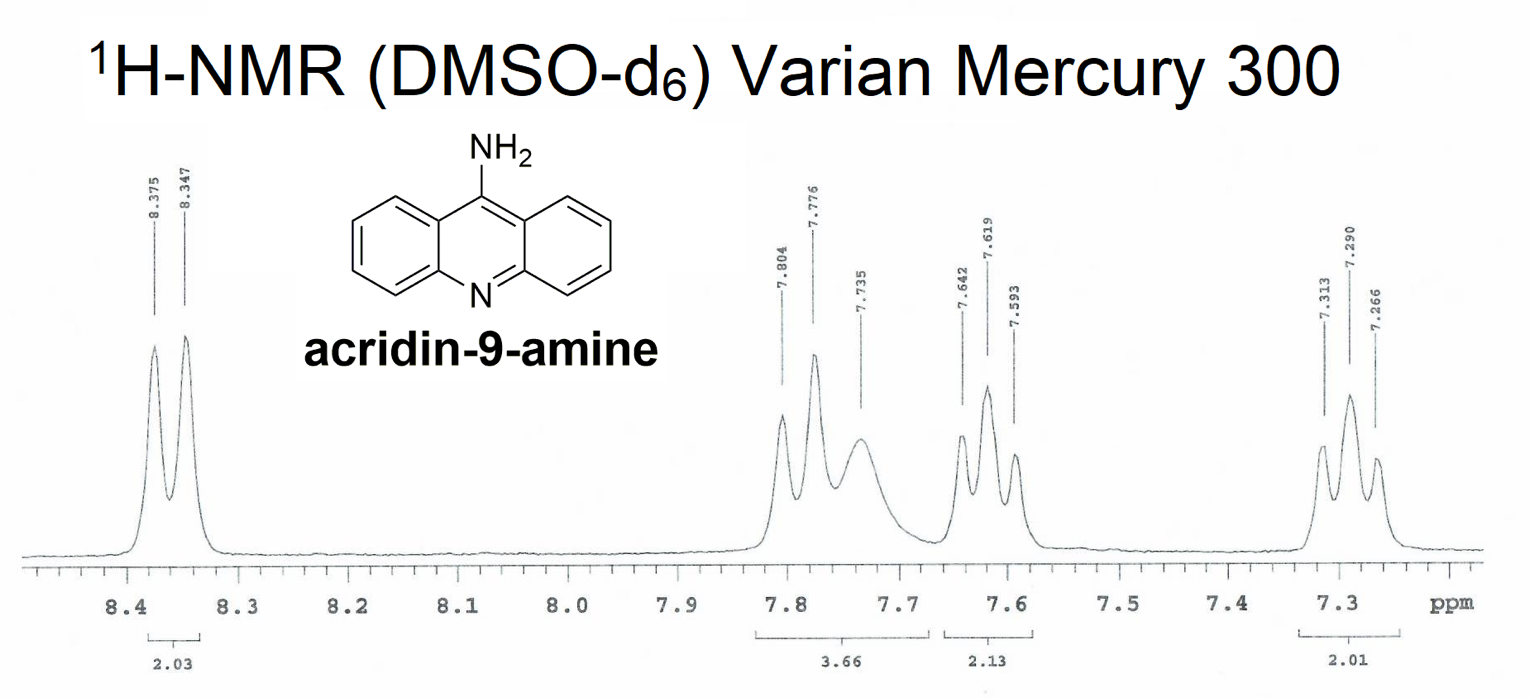


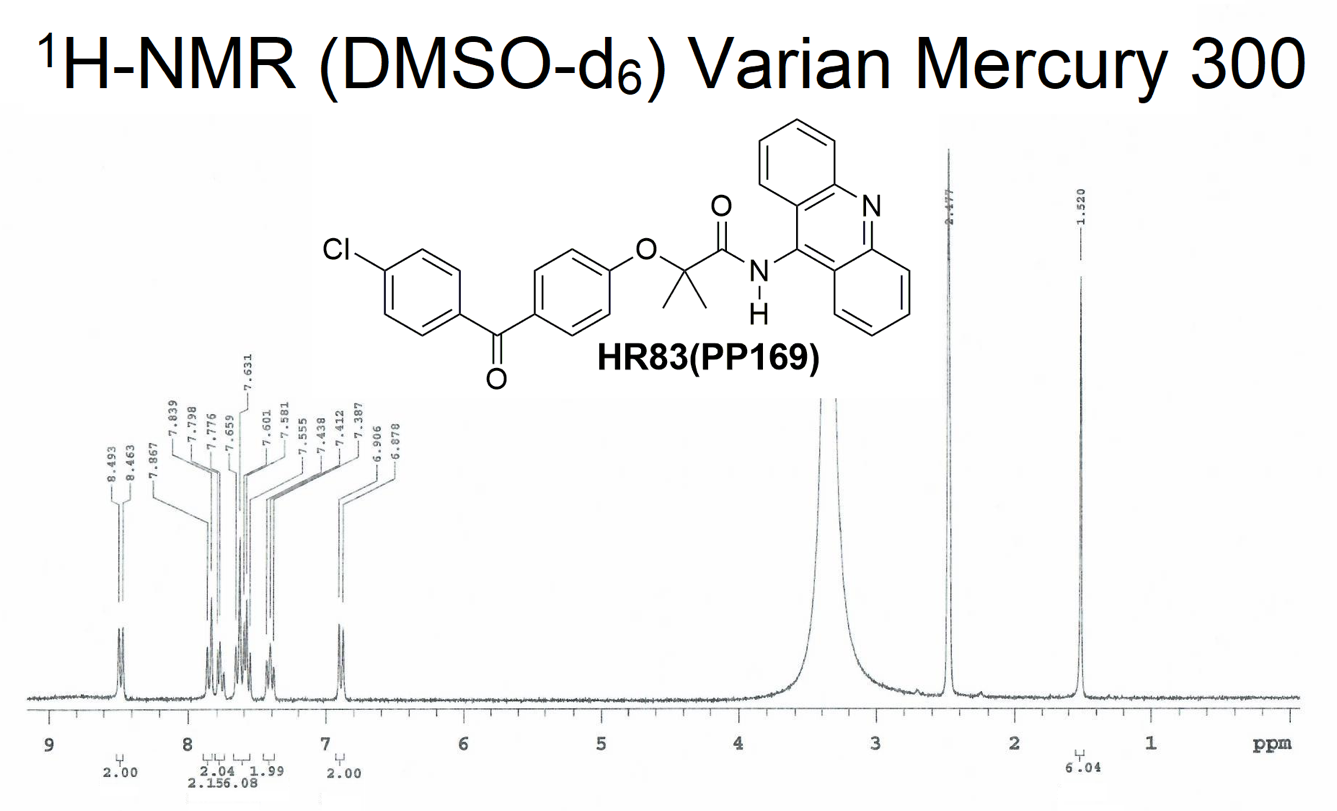


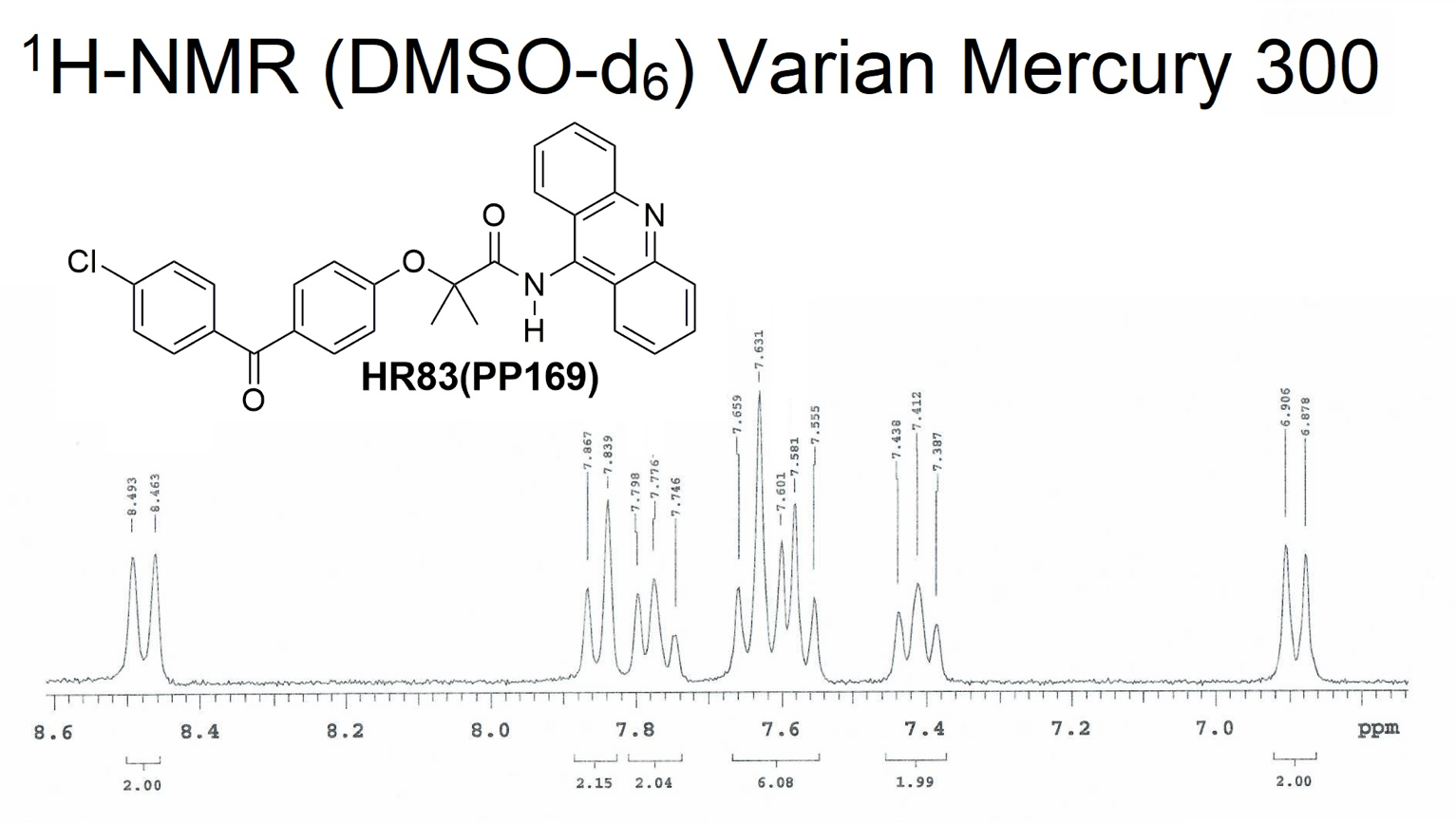


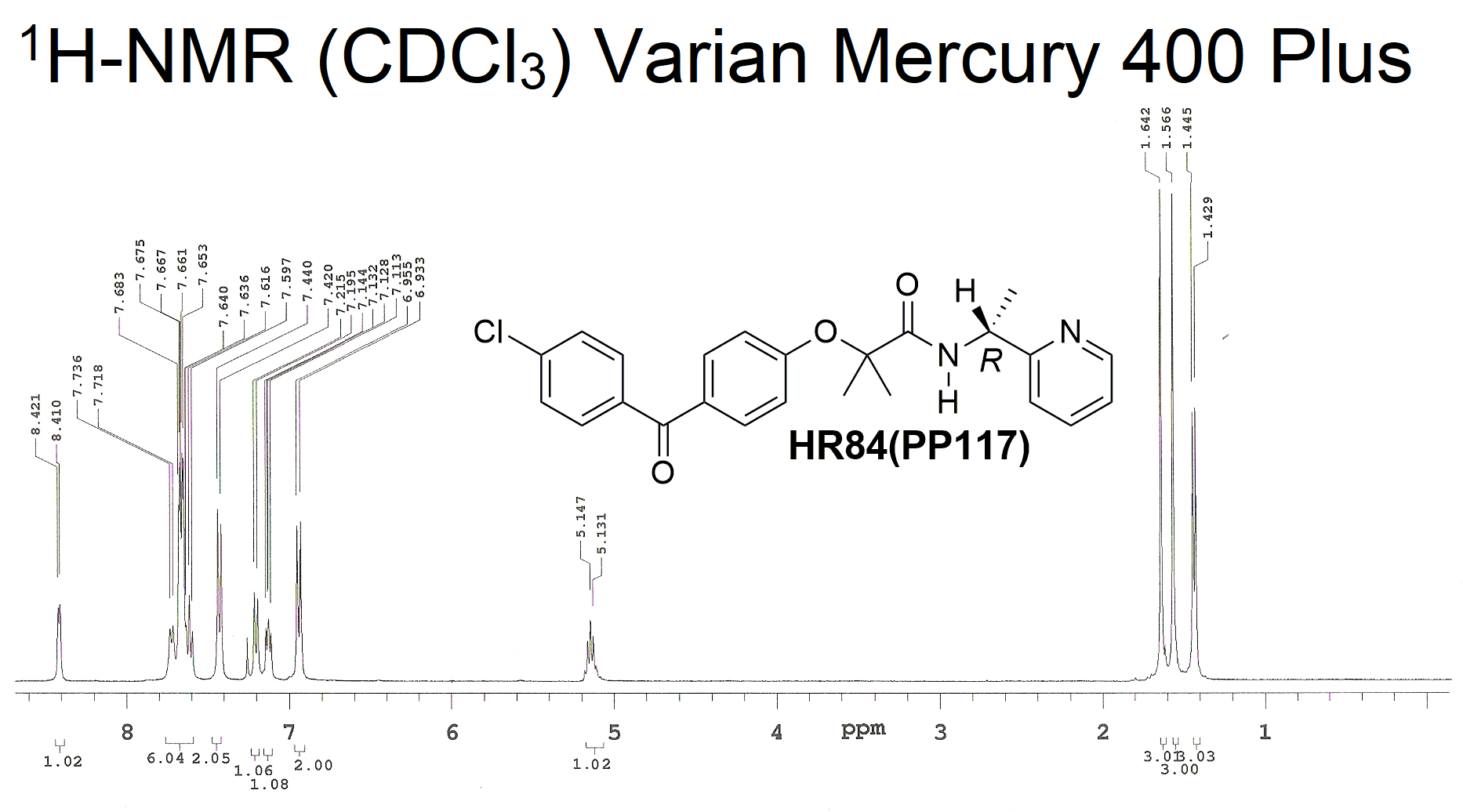


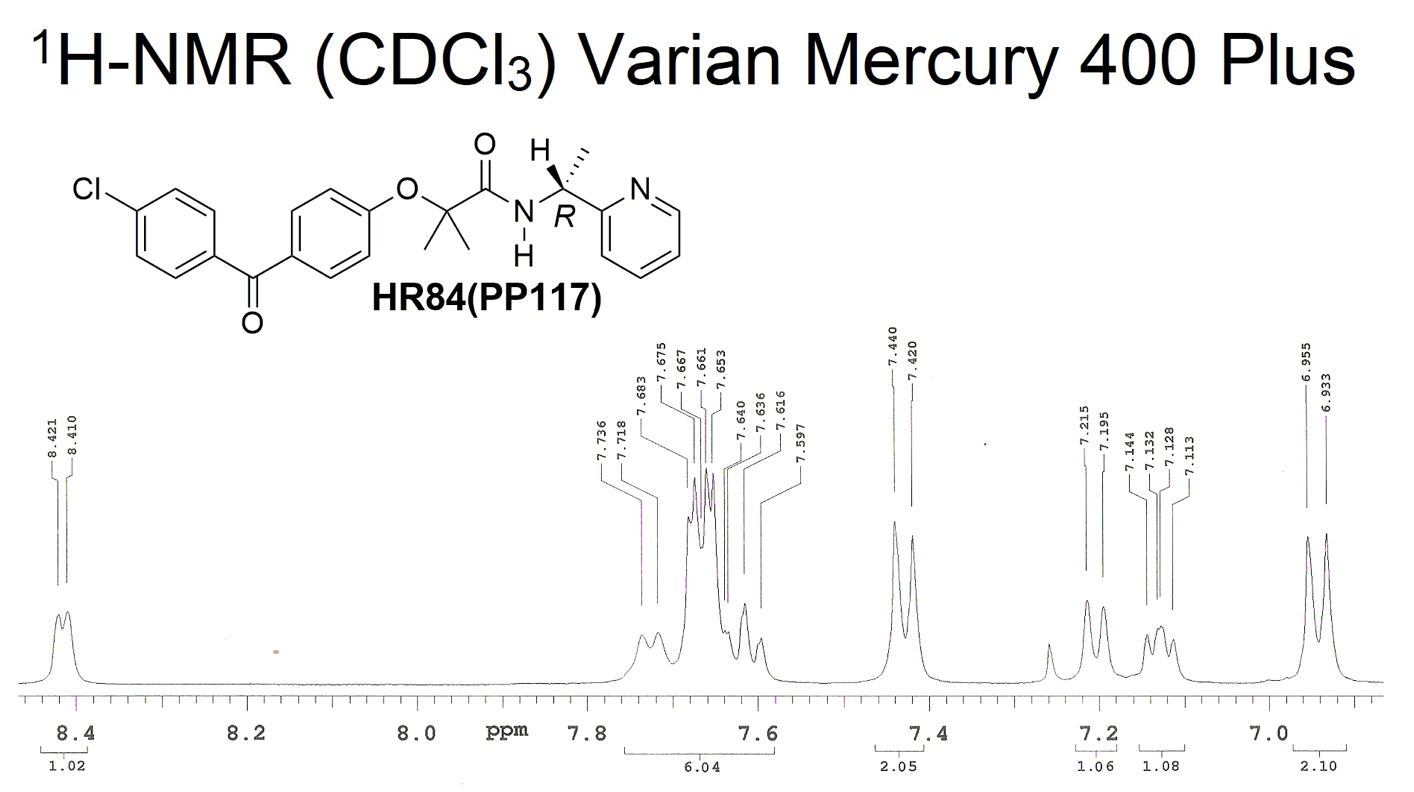


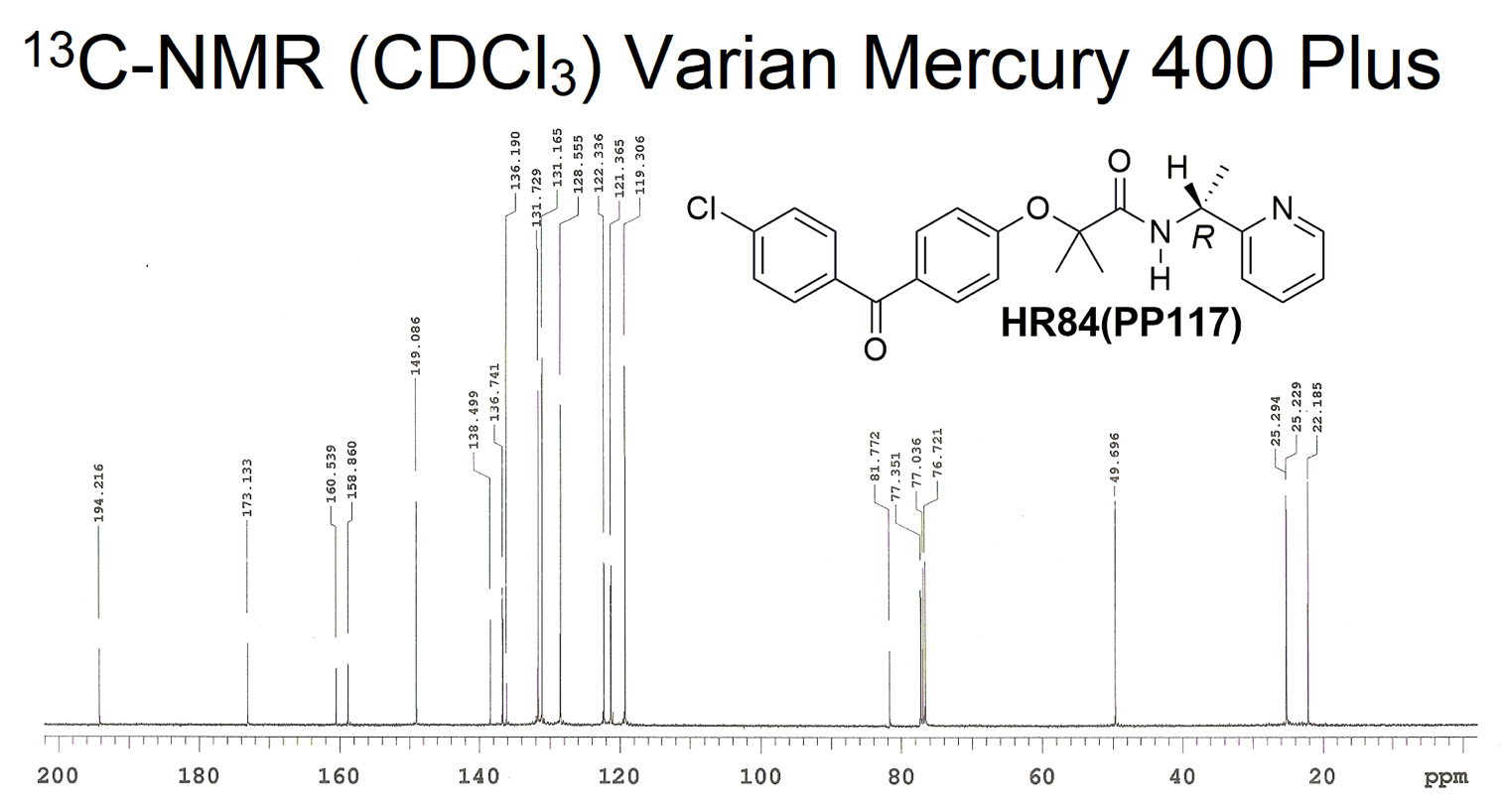


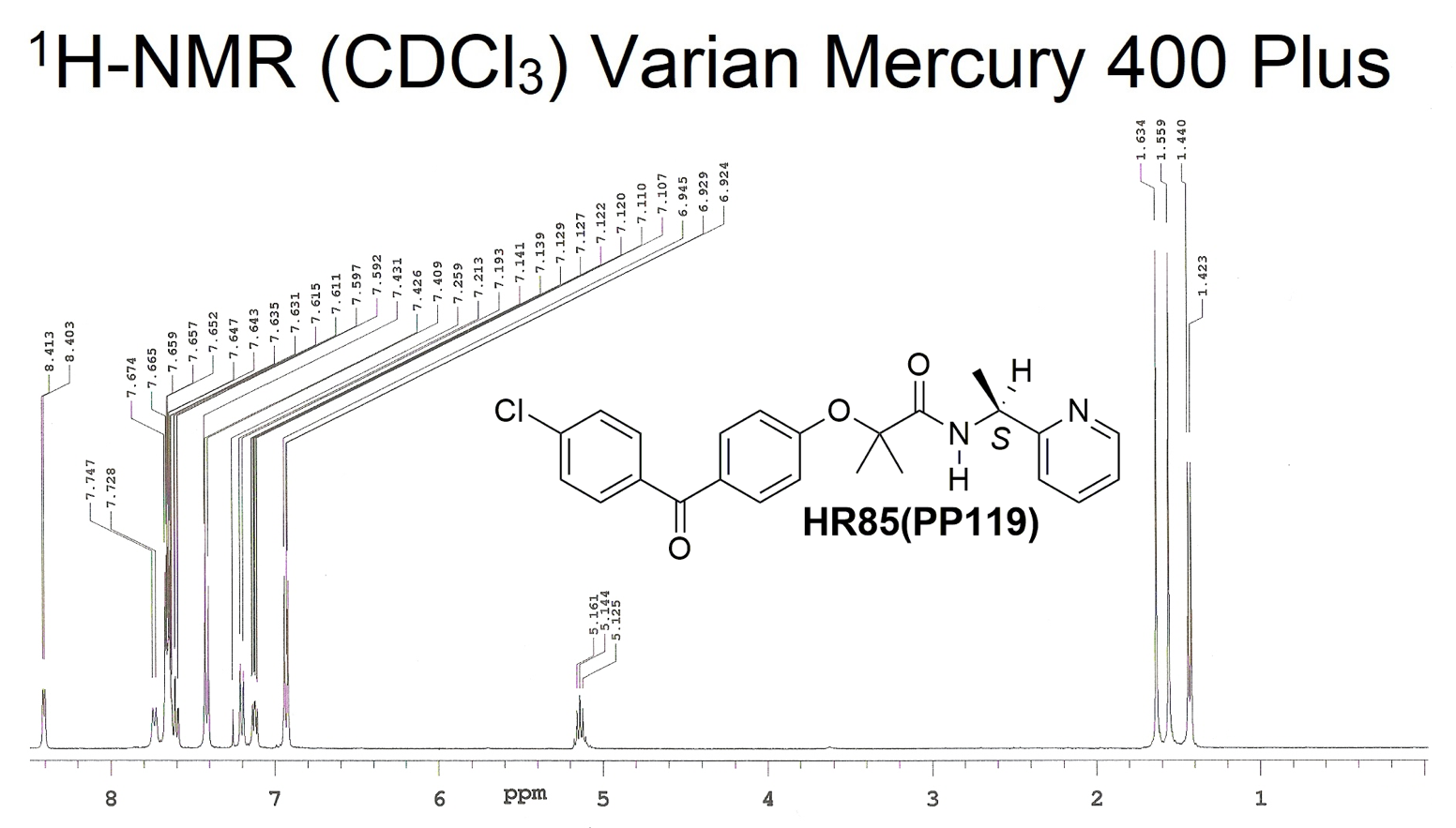


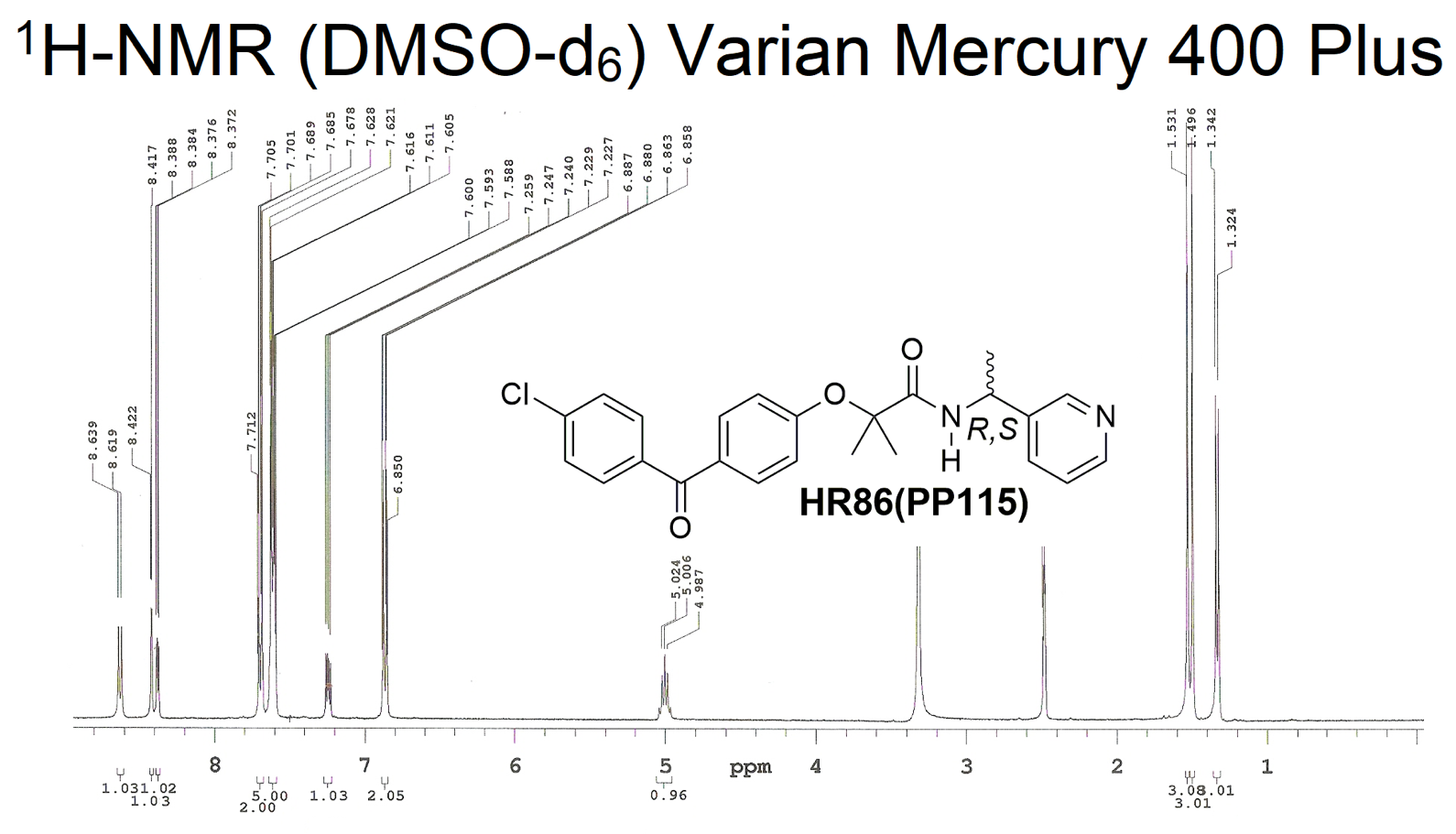


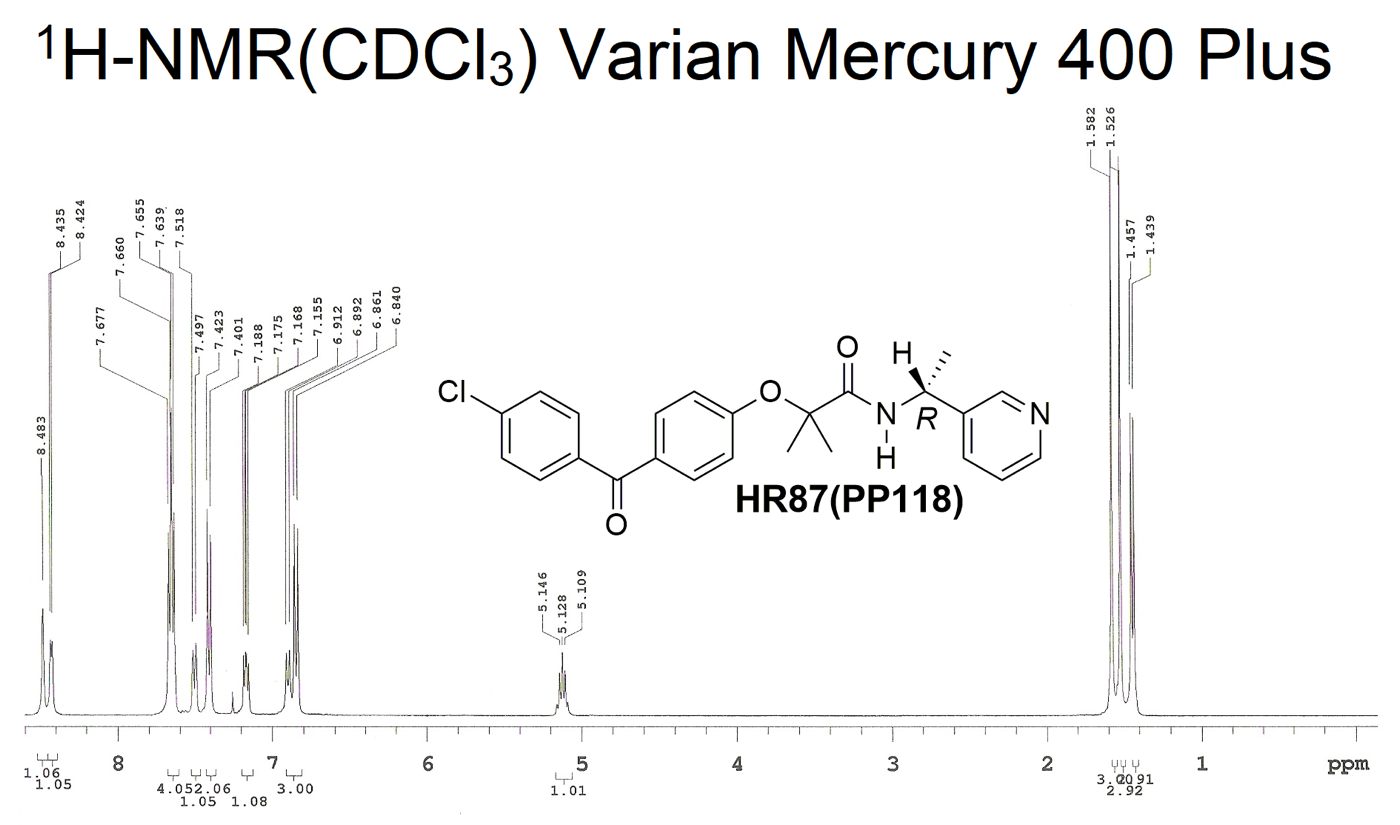


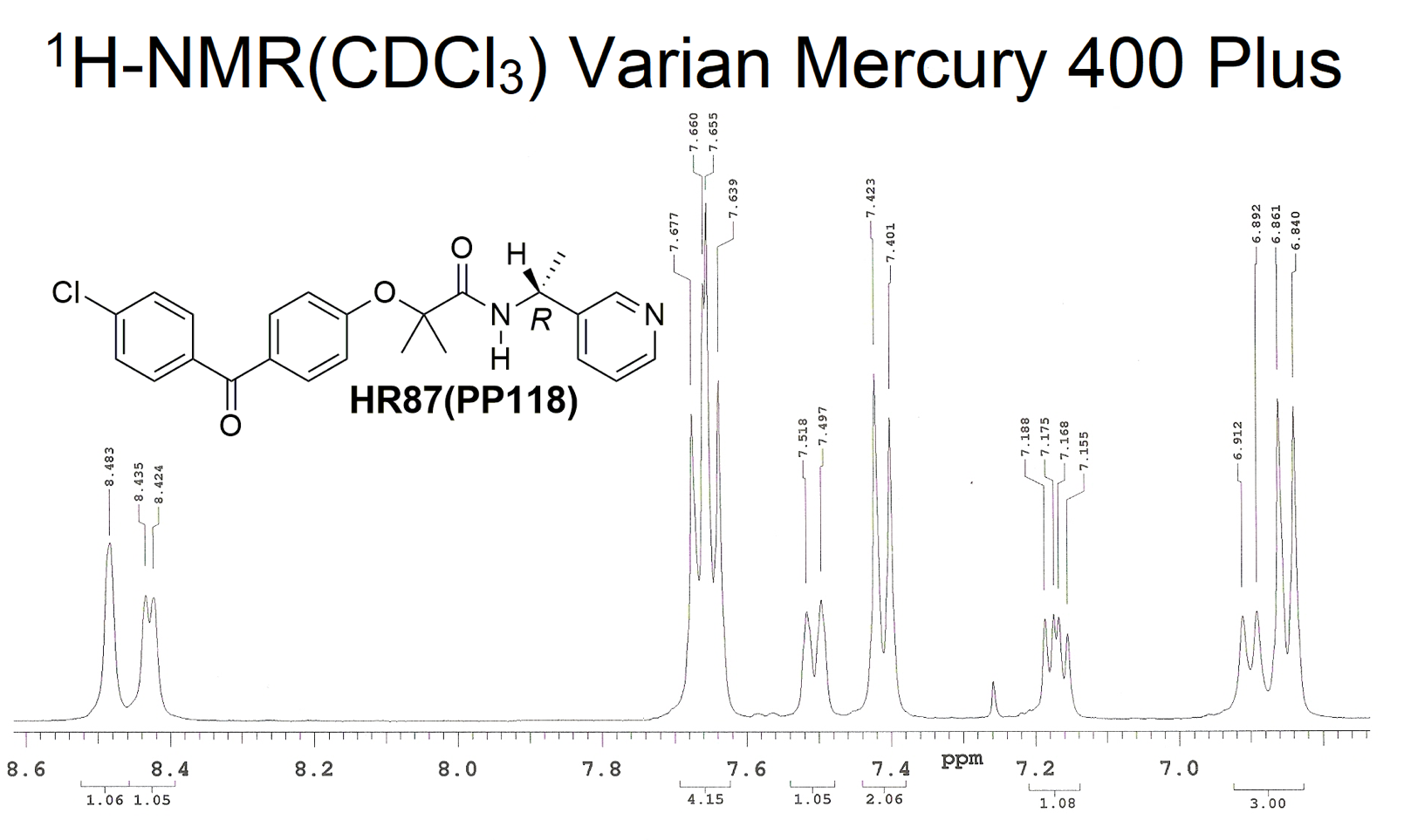


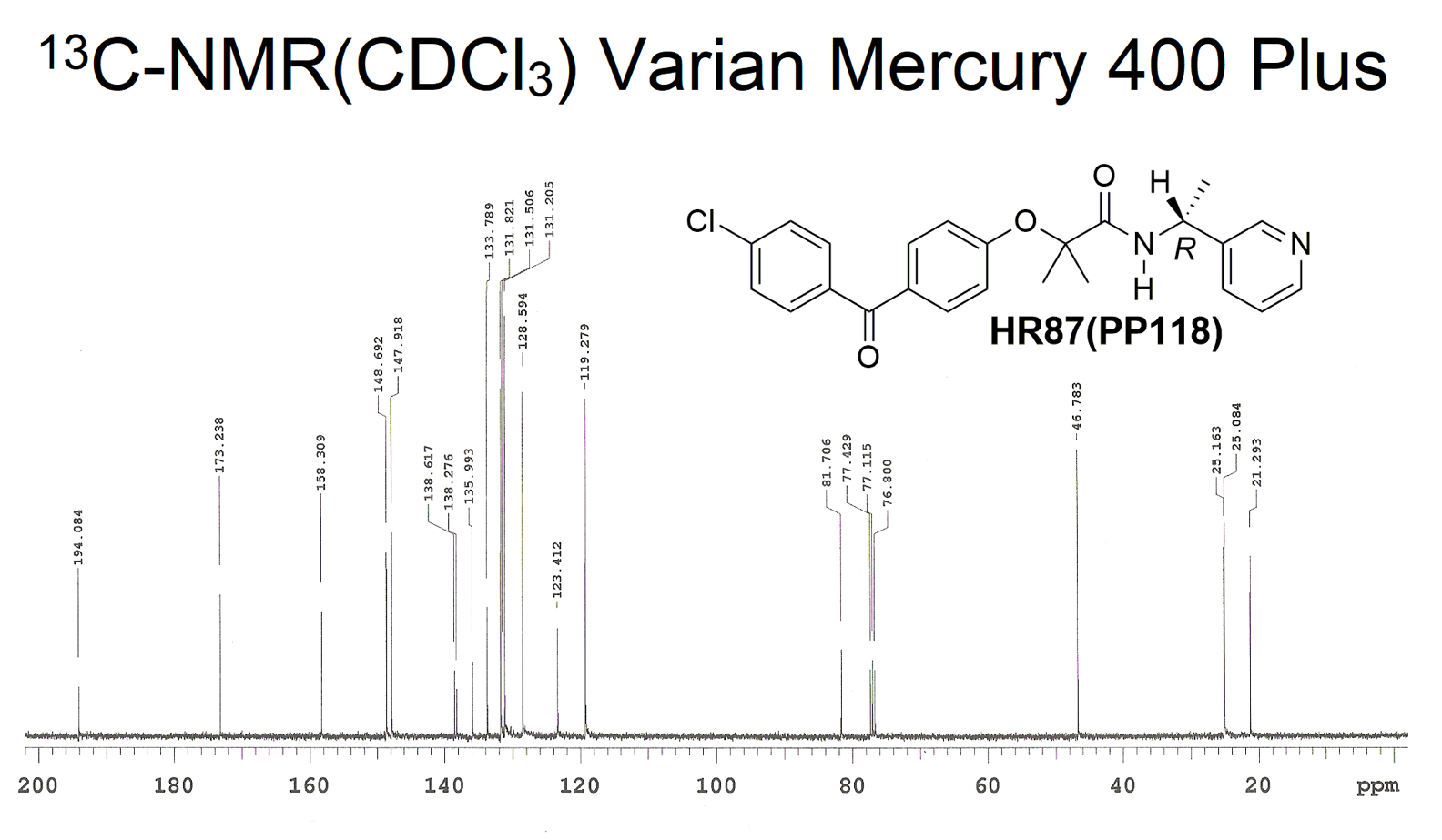


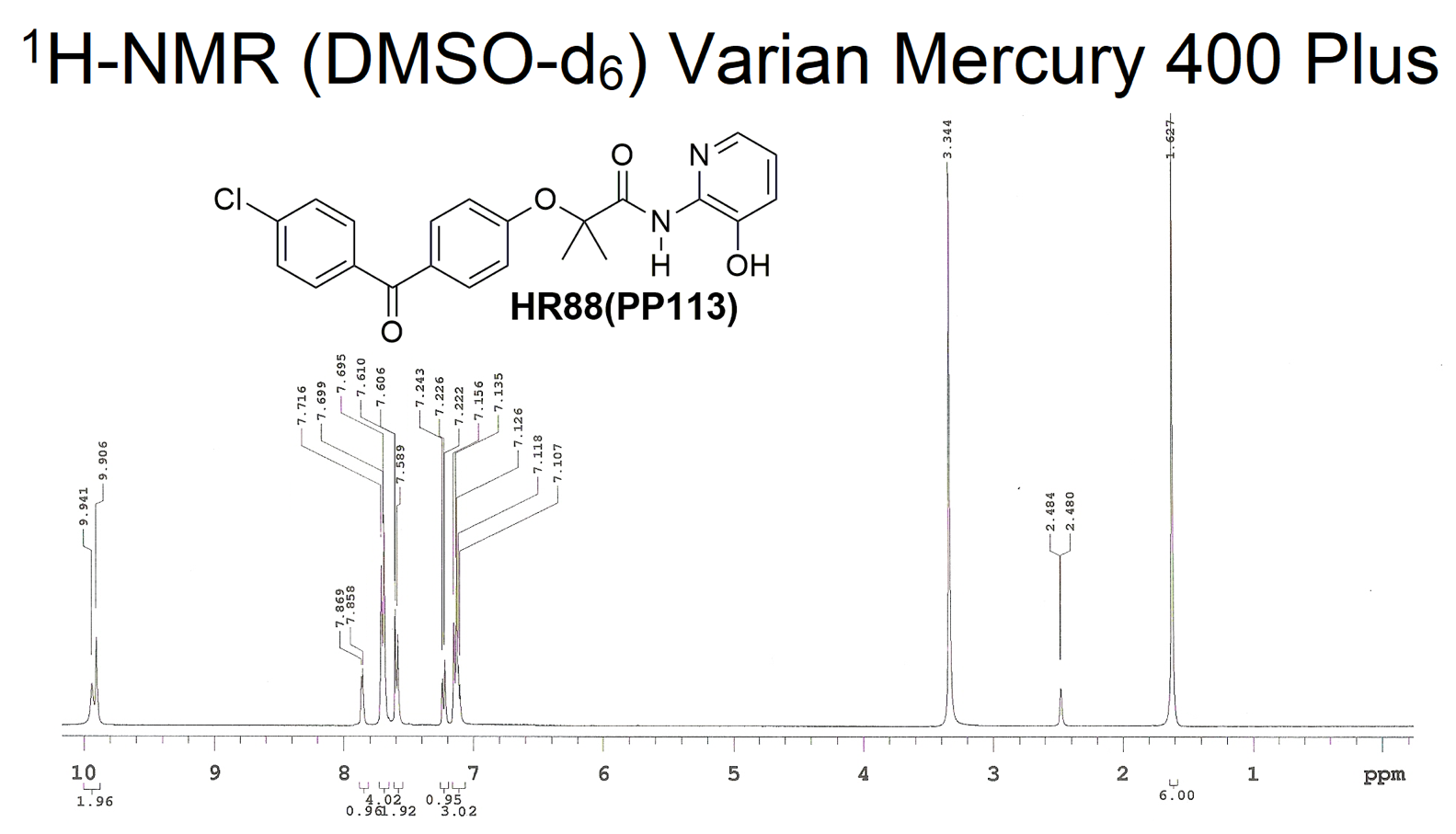


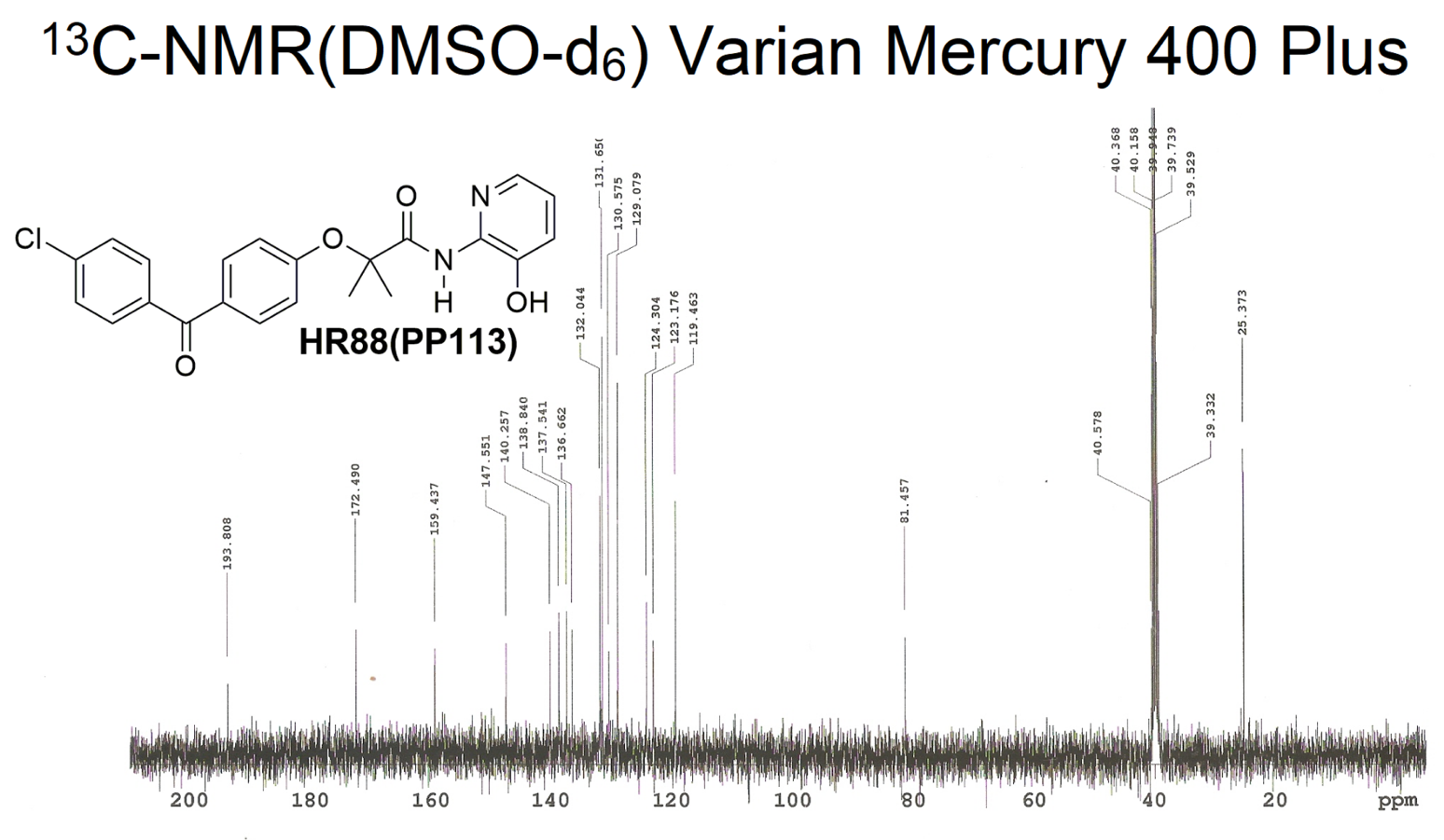


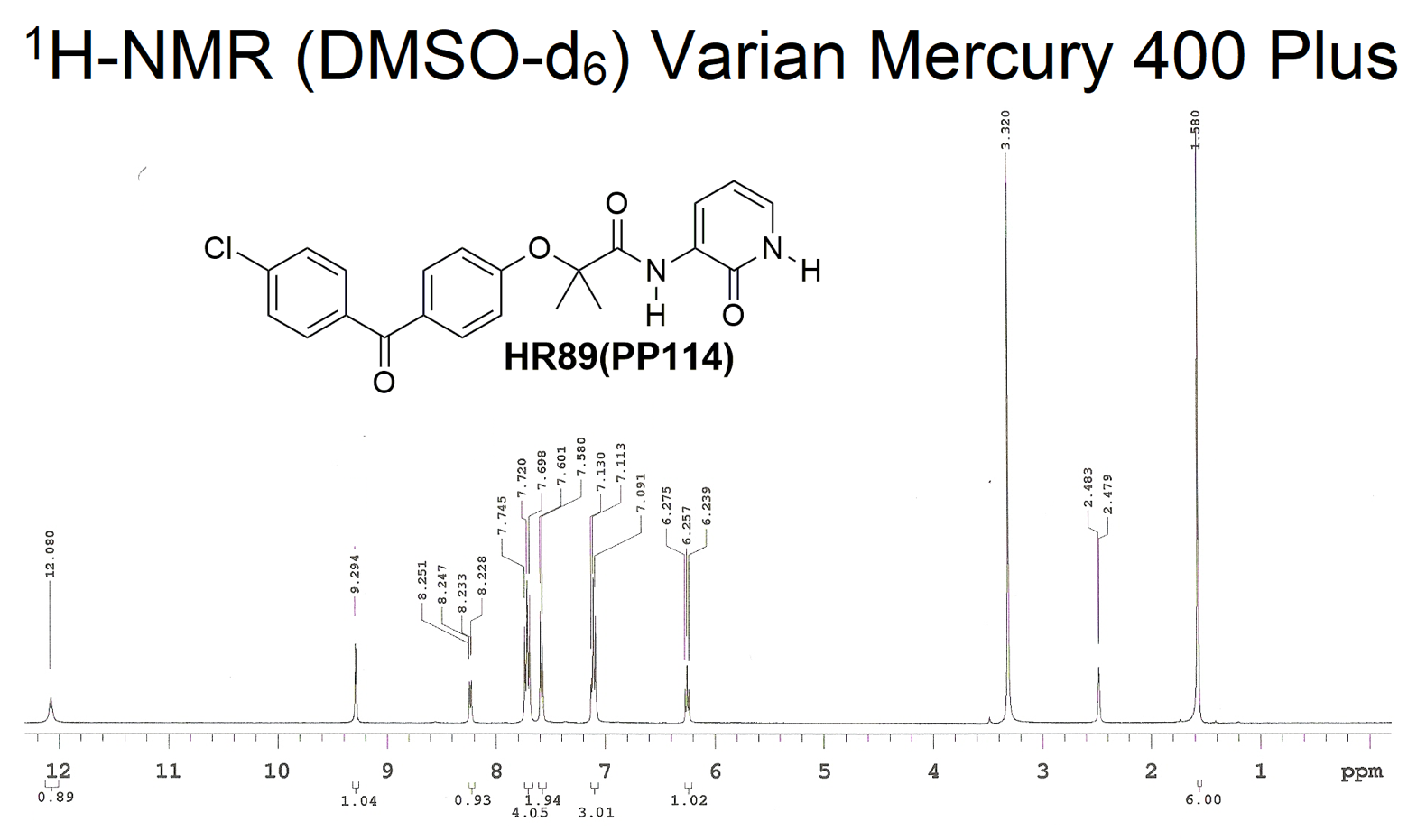


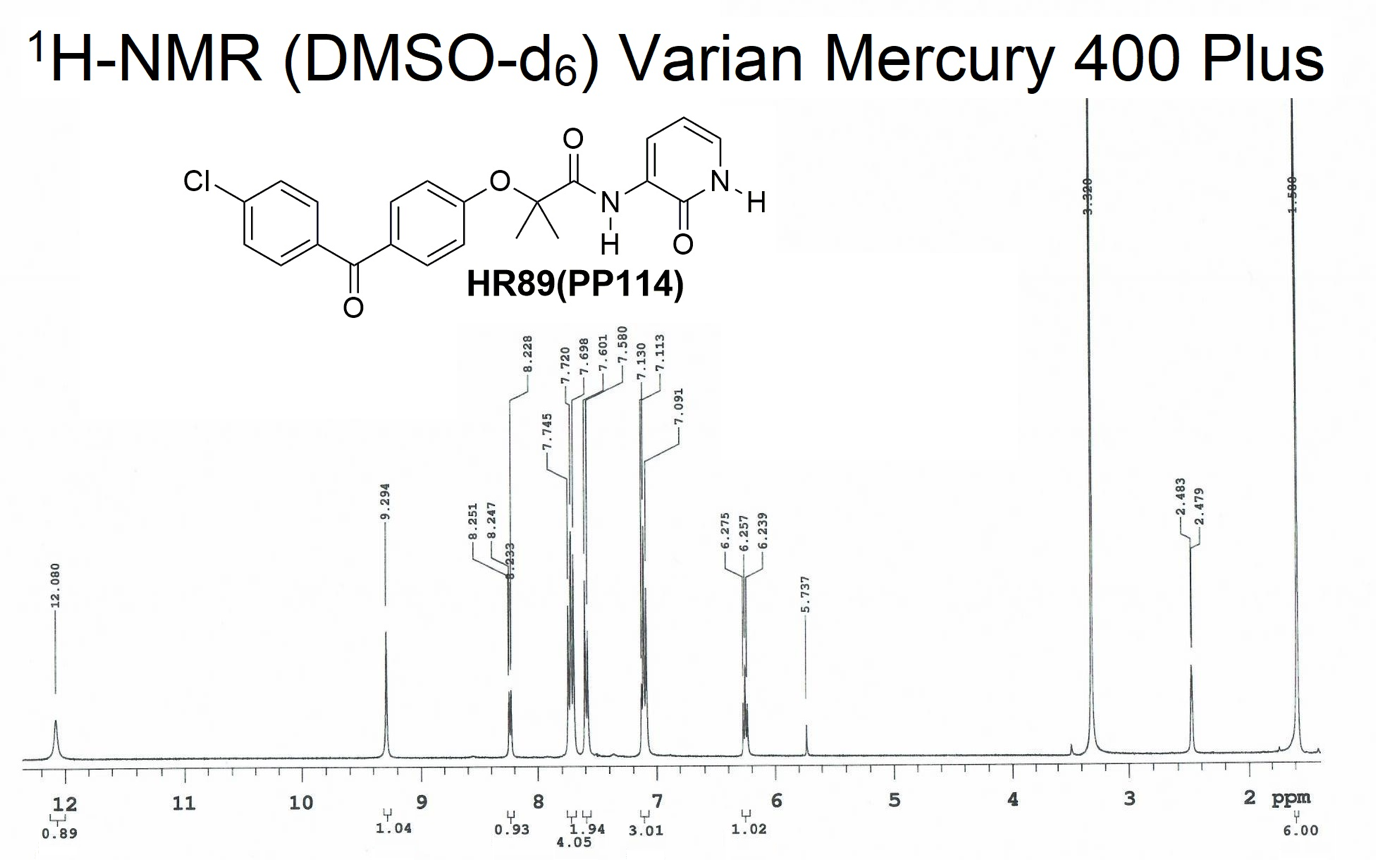


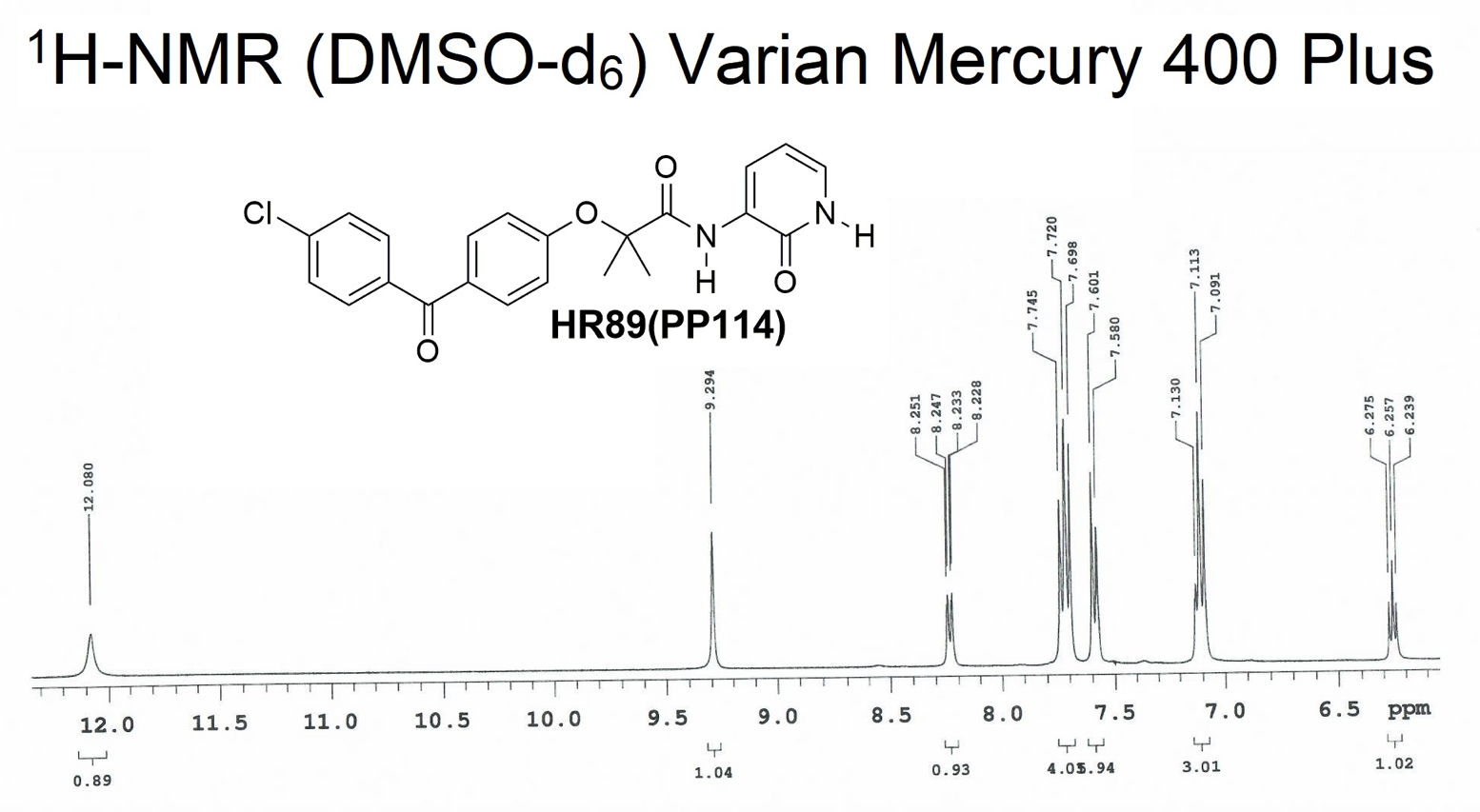


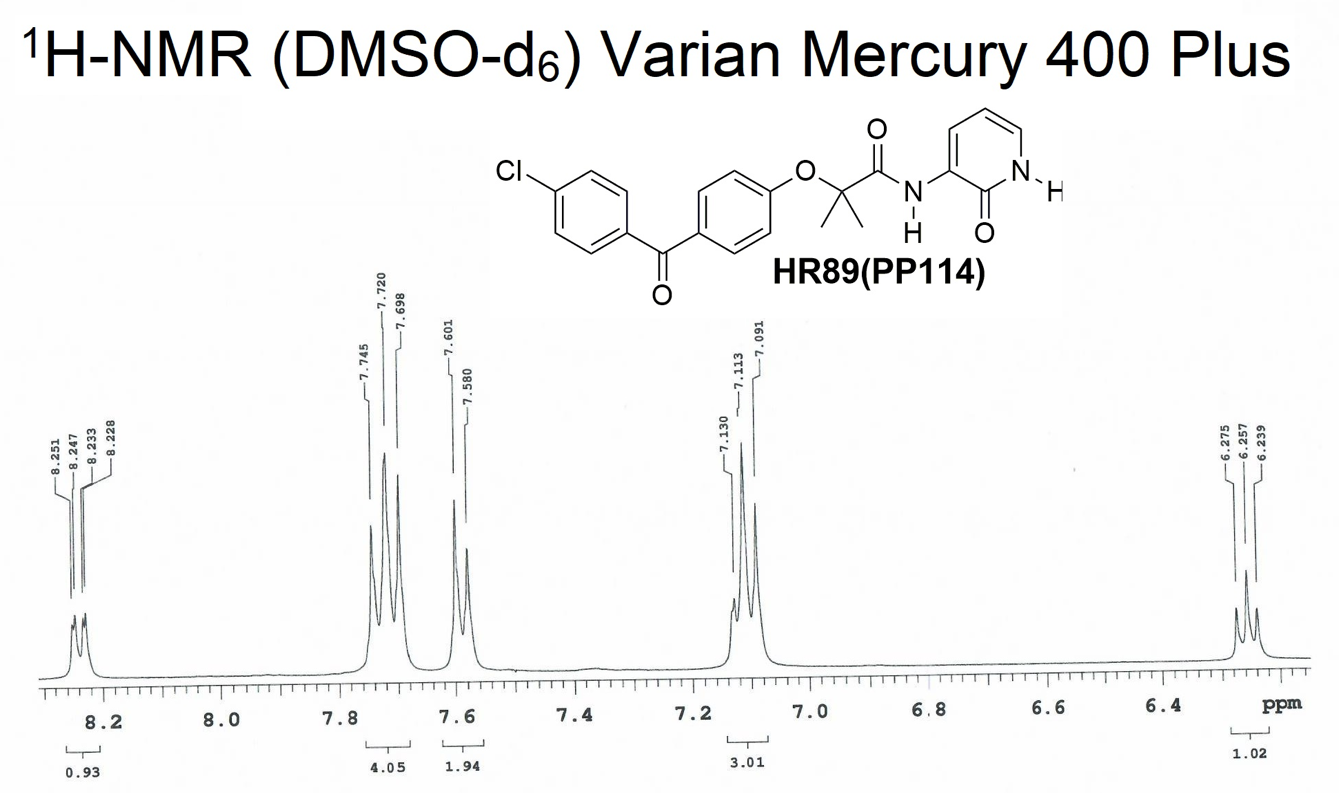


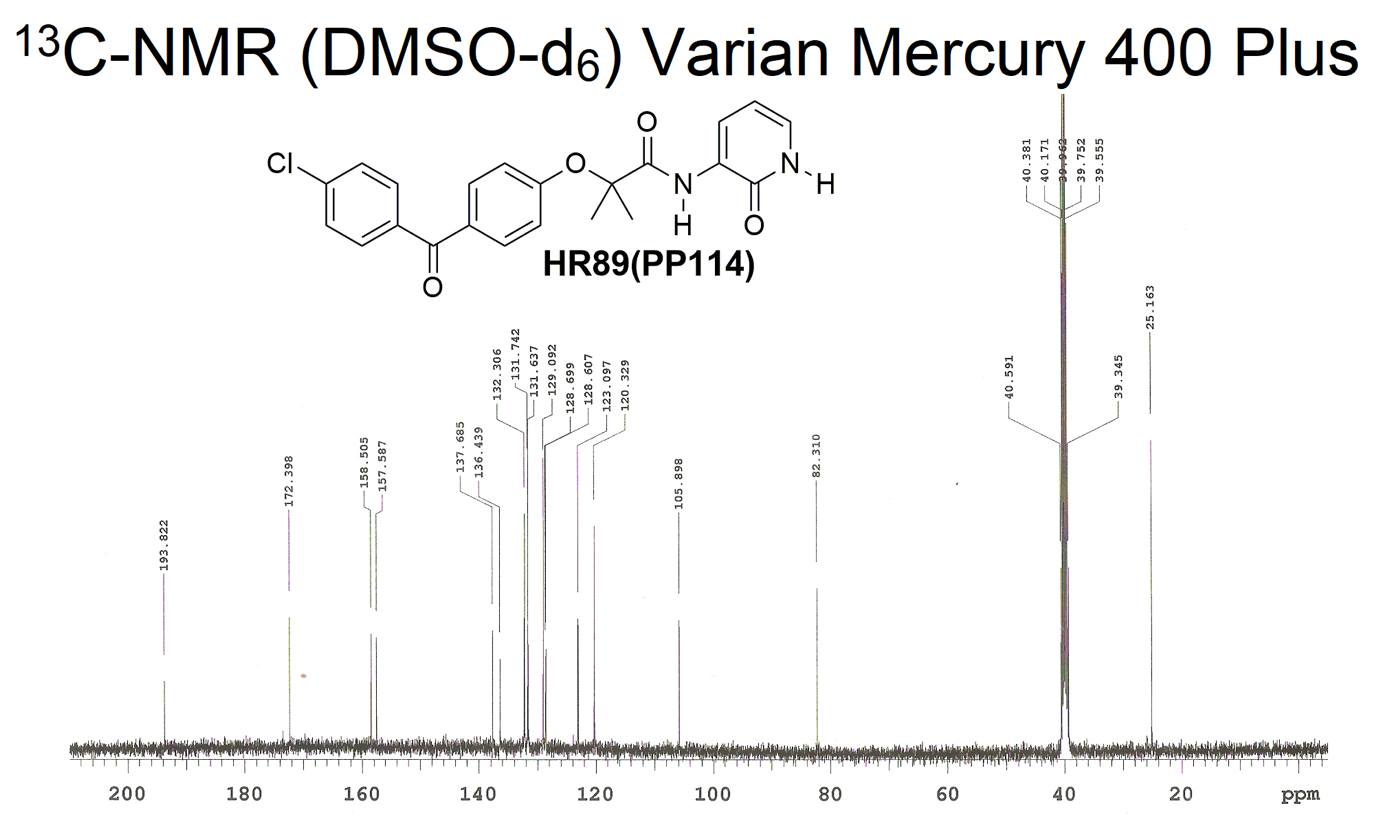


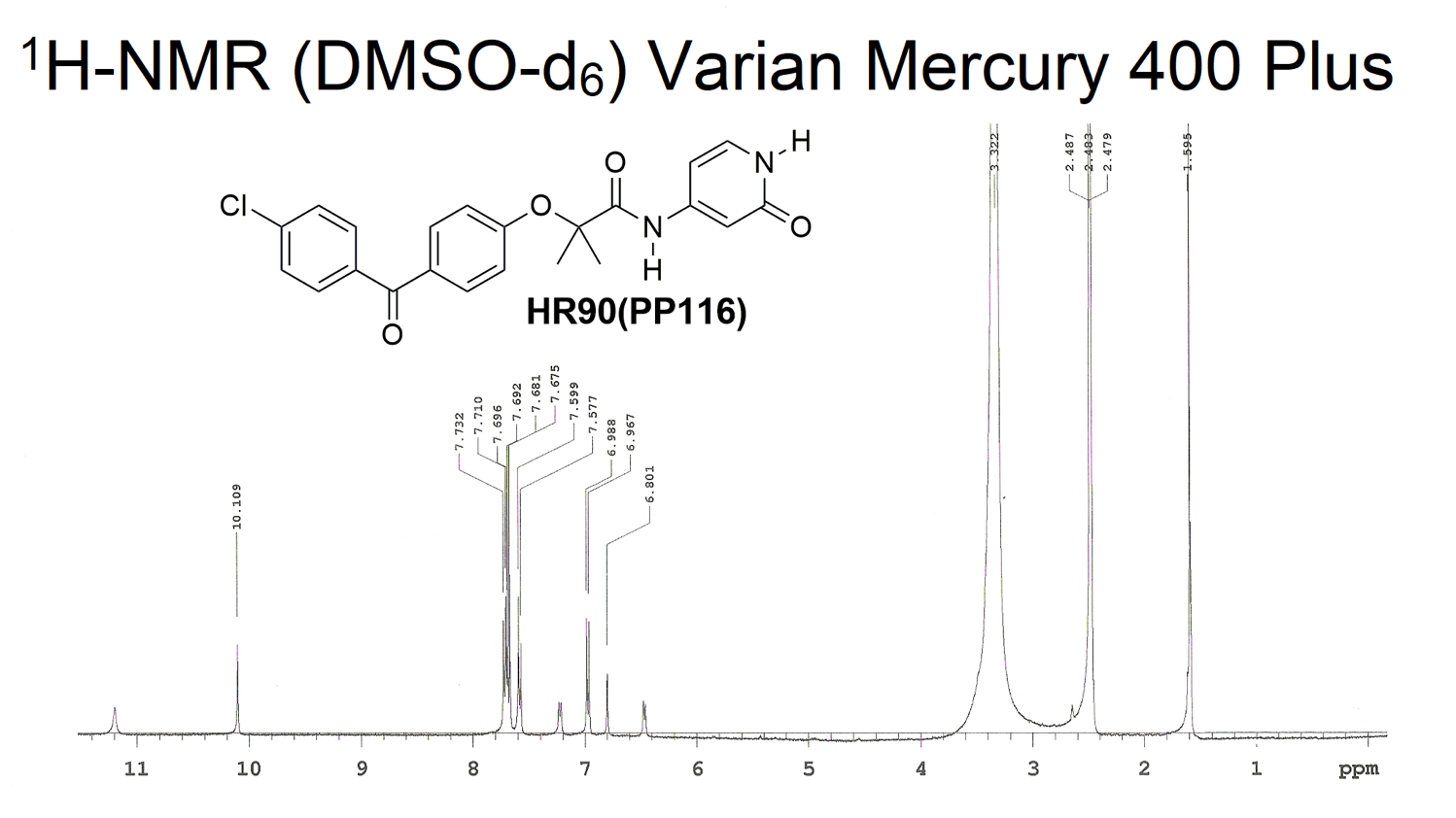


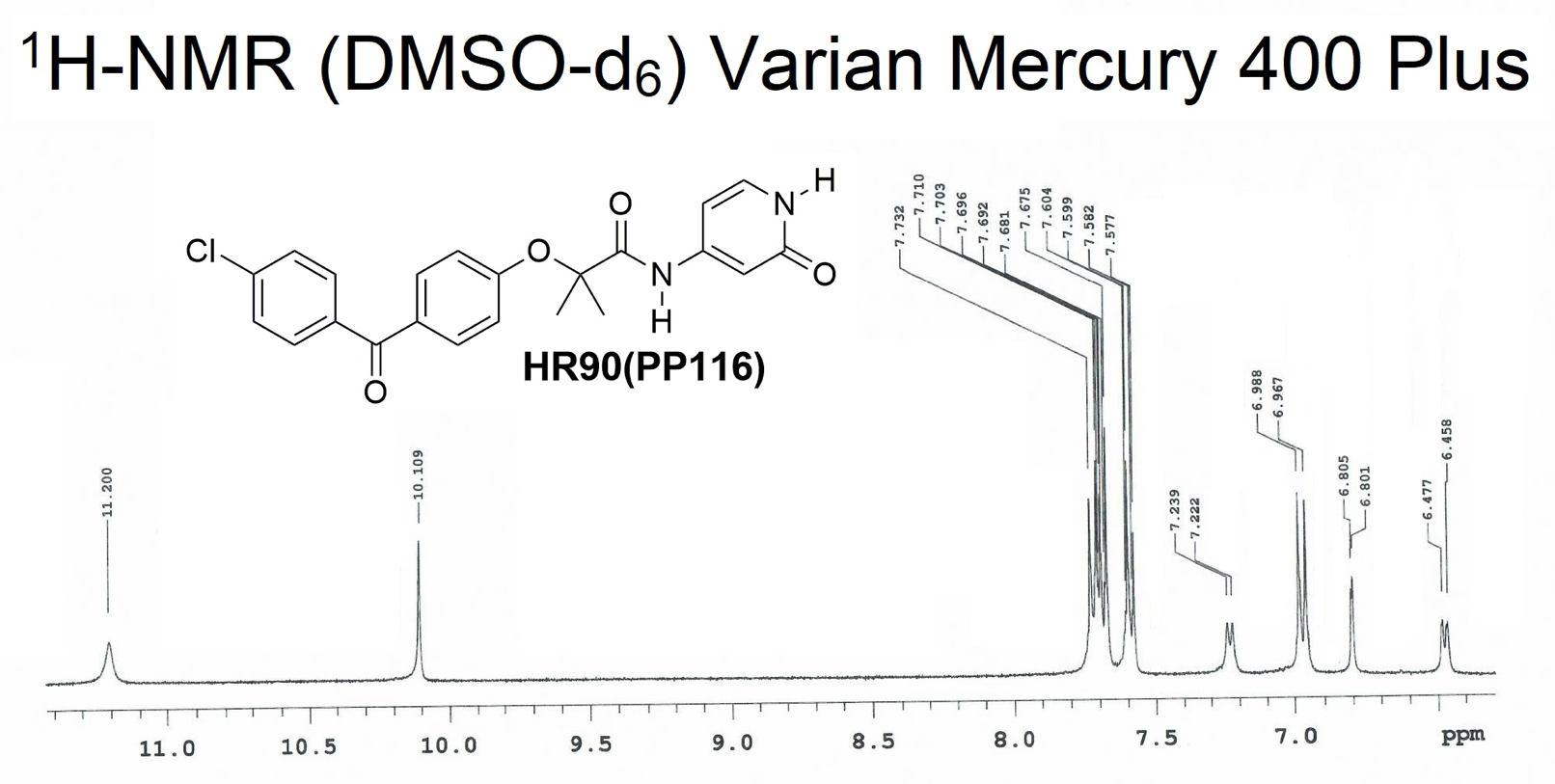


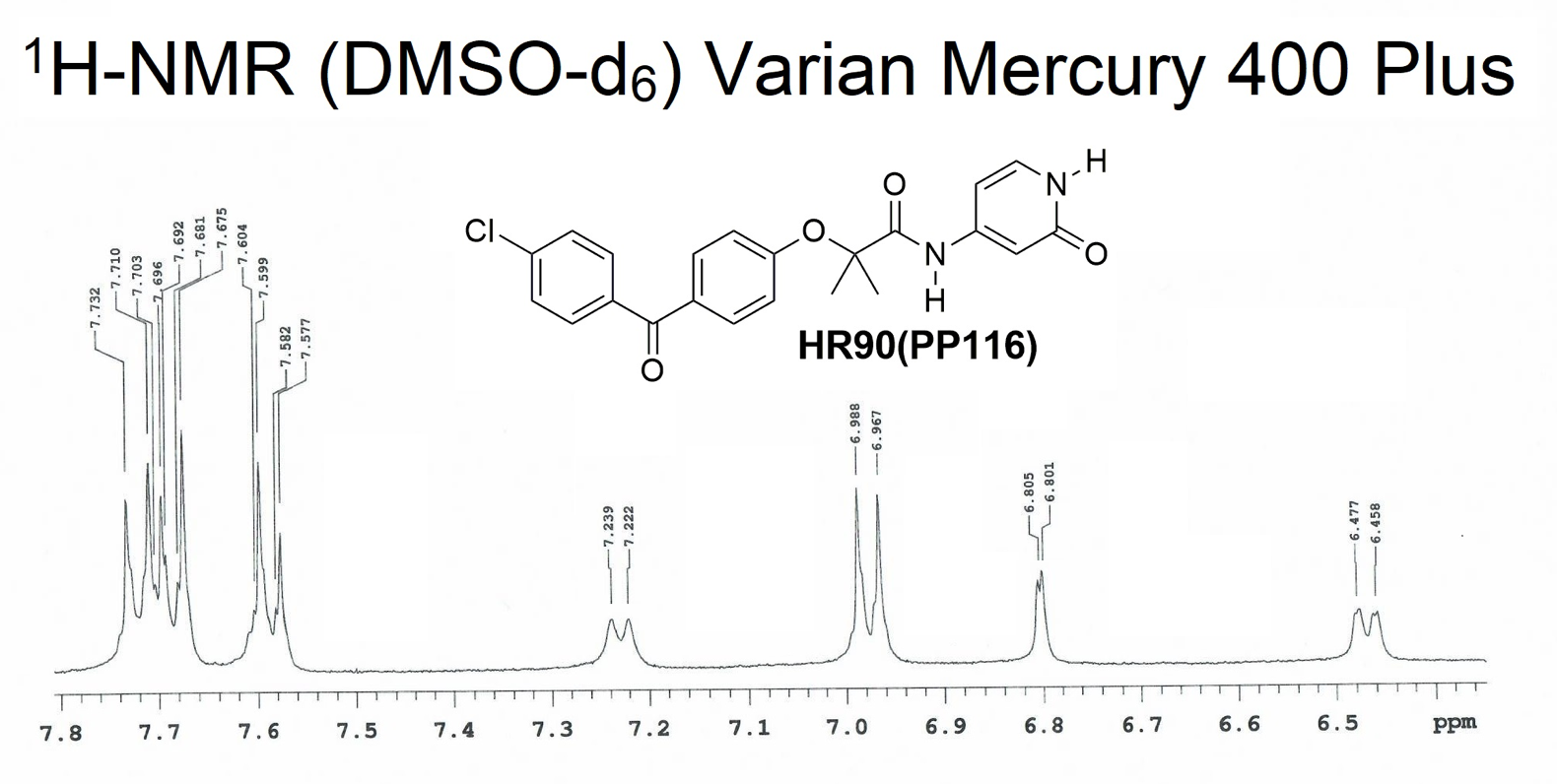


***2. MarvinSketch (Product version 22.21) Generated data including common chemotherapy drugs for CNS tumors compared to Pyridine-BPA variants:***

***(HR48-HR90)***

The most common chemotherapy drug combination used to treat brain and spinal cord tumors is PCV (procarbazine, lomustine and vincristine)

| Calc. | MarvinSketch | | IP | RB | HLB | logS | PL | MPA | MSA | HBA | MR | hERG_AM_ | hERG_CM_ |
| --- | --- | --- | --- | --- | --- | --- | --- | --- | --- | --- | --- | --- | --- |
|  |  | T_0_ | NA | 1 | 18.31 | -0.90 | 16.08 | 26.31 | 232.02 | 7 | 47.86 | 3.67 | safe |
| ClogP | -0.28 | 1.00 |  |  |  |  |  |  |  |  |  |  |  |
| ClogD | -0.28 | 1.00 |  |  |  |  |  |  |  |  |  |  |  |
| MW | 194.15 | 1.00 |  |  |  |  |  |  |  |  |  |  |  |
| PSA | 105.94 | 0.47 |  |  |  |  |  |  |  |  |  |  |  |
| HBD | 2.00 | 0.50 |  |  |  |  |  |  |  |  |  |  |  |
| pKa | -0.96 | 1.00 |  |  |  |  |  |  |  |  |  |  |  |
| MPO | 4.97 | |  |  |  |  |  |  |  |  |  |  |  |
| BBB | 3.44 | |  |  |  |  |  |  |  |  |  |  |  |
| LogBB | -1.47 | |  |  |  |  |  |  |  |  |  |  |  |

IP = Isoelectronic Point; RB = Number of rotatable bonds; HLB = hydrophilic-lipophilic balance; logS = water solubility; PL = polarizability(Å^3^); Minimal Projection Area (Å^2^); MSA = molecular surface area; HBA = hydrogen bond acceptors; MR = Molar refractivity; ClogD = distribution at pH = 7.4; PSA = polar surface area (Å^2^); HBD = hydrogen bond donors; pKa = estimated acid strength; MPO = score for CNS penetration; LogBB = blood-brain distribution (0.152ClogP – 0.0148PSA + 0.138); hERG = estimated pIC50 (pAct) value for hERG (the human ether-a-go-go (hERG) ion channel); hERG_AM_ = hERG activity model; hERG_CM_ = hERG classification model

| BBB Score Calculator | | |
| --- | --- | --- |
| Property | Value | T_0_ |
| ARO_R_SCORE | 2 | 1.00 |
| HA_SCORE | 14 | 0.89 |
| MWHBN_SCORE | 0.43 | 0.36 |
| TPSA_SCORE | 105.94 | 0.26 |
| PKA_SCORE | 8.81 | 1.00 |
| BBB SCORE | | 3.44 |

ARO_R_SCORE = Number of aromatic rings; HA_SCORE = Number of heavy atoms. MWHNM_SCORE = [-0.5MW(HBA+HBD)] where MW = molecular weight, HBA = number of hydrogen bond acceptor sites, and HBD = number of hydrogen bond donors; TPSA = topological polar surface area; PKA_SCORE = Acidity strength. Gupta, M.; Lee, H. J.; Barden, C. J.; Weaver, D. “The Blood-Brain Barrier (BBB) Score” J. Med. Chem. 2019, 62 (21), 9824-9836.

| Calc. | MarvinSketch | | IP | RB | HLB | logS | PL | MPA | MSA | HBA | MR | hERG_AM_ | hERG_CM_ |
| --- | --- | --- | --- | --- | --- | --- | --- | --- | --- | --- | --- | --- | --- |
|  |  |  | NA | 5 | 14.05 | -2.38 | 17.32 | 29.69 | 61.77 | 4 | 46.98 | 3.49 | safe |
| ClogP | 1.02 | 1.00 |  |  |  |  |  |  |  |  |  |  |  |
| ClogD | 1.02 | 1.00 |  |  |  |  |  |  |  |  |  |  |  |
| MW | 214.05 | 1.00 |  |  |  |  |  |  |  |  |  |  |  |
| PSA | 61.77 | 1.00 |  |  |  |  |  |  |  |  |  |  |  |
| HBD | 1.00 | 0.75 |  |  |  |  |  |  |  |  |  |  |  |
| pKa | -5.34 | 1.00 |  |  |  |  |  |  |  |  |  |  |  |
| MPO | 5.75 | |  |  |  |  |  |  |  |  |  |  |  |
| BBB | 4.26 | |  |  |  |  |  |  |  |  |  |  |  |
| LogBB | -0.62 | |  |  |  |  |  |  |  |  |  |  |  |

IP = Isoelectronic Point; RB = Number of rotatable bonds; HLB = hydrophilic-lipophilic balance; logS = water solubility; PL = polarizability(Å^3^); Minimal Projection Area (Å^2^); MSA = molecular surface area; HBA = hydrogen bond acceptors; MR = Molar refractivity; ClogD = distribution at pH = 7.4; PSA = polar surface area; HBD = hydrogen bond donors; pKa = estimated acid strength; MPO = score for CNS penetration; LogBB = blood-brain distribution (0.152ClogP – 0.0148PSA + 0.138); hERG = estimated pIC50 (pAct) value for hERG (the human ether-a-go-go (hERG) ion channel); hERG_AM_ = hERG activity model; hERG_CM_ = hERG classification model

| BBB Score Calculator | | |
| --- | --- | --- |
| Property | Value | T_0_ |
| ARO_R_SCORE | 0 | 0.34 |
| HA_SCORE | 12 | 0.79 |
| HWHBN_SCORE | 0.21 | 1.00 |
| TPSA_SCORE | 61.77 | 0.57 |
| PKA_SCORE | 8.81 | 1.00 |
| BBB_SCORE | | 4.26 |

ARO_R_SCORE = Number of aromatic rings; HA_SCORE = Number of heavy atoms. MWHNM_SCORE = [-0.5MW(HBA+HBD)] where MW = molecular weight, HBA = number of hydrogen bond acceptor sites, and HBD = number of hydrogen bond donors; TPSA = topological polar surface area; PKA_SCORE = Acidity strength. Gupta, M.; Lee, H. J.; Barden, C. J.; Weaver, D. “The Blood-Brain Barrier (BBB) Score” J. Med. Chem. 2019, 62 (21), 9824-9836.

| Calc. | MarvinSketch | | IP | RB | HLB | logS | PL | MPA | MSA | HBA | MR | hERG_AM_ | hERG_CM_ |
| --- | --- | --- | --- | --- | --- | --- | --- | --- | --- | --- | --- | --- | --- |
|  |  | T_0_ | NA | 4 | 11.95 | -3.28 | 21.92 | 33.97 | 351.15 | 4 | 58.65 | 3.70 | safe |
| ClogP | 2.16 | 1.00 |  |  |  |  |  |  |  |  |  |  |  |
| ClogD | 2.16 | 0.92 |  |  |  |  |  |  |  |  |  |  |  |
| MW | 233.70 | 1.00 |  |  |  |  |  |  |  |  |  |  |  |
| PSA | 61.77 | 1.00 |  |  |  |  |  |  |  |  |  |  |  |
| HBD | 1.00 | 0.75 |  |  |  |  |  |  |  |  |  |  |  |
| pKa | -5.35 | 1.00 |  |  |  |  |  |  |  |  |  |  |  |
| MPO | 5.67 | |  |  |  |  |  |  |  |  |  |  |  |
| BBB | 4.40 | |  |  |  |  |  |  |  |  |  |  |  |
| LogBB | -0.45 | |  |  |  |  |  |  |  |  |  |  |  |

IP = Isoelectronic Point; RB = Number of rotatable bonds; HLB = hydrophilic-lipophilic balance; logS = water solubility; PL = polarizability(Å^3^); Minimal Projection Area (Å^2^); MSA = molecular surface area; HBA = hydrogen bond acceptors; MR = Molar refractivity; ClogD = distribution at pH = 7.4; PSA = polar surface area; HBD = hydrogen bond donors; pKa = estimated acid strength; MPO = score for CNS penetration; LogBB = blood-brain distribution (0.152ClogP – 0.0148PSA + 0.138); hERG = estimated pIC50 (pAct) value for hERG (the human ether-a-go-go (hERG) ion channel); hERG_AM_ = hERG activity model; hERG_CM_ = hERG classification model

| BBB Score Calculator | | |
| --- | --- | --- |
| Property | Value | T_0_ |
| ARO_R_SCORE | 0 | 0.34 |
| HA_SCORE | 15 | 0.93 |
| HWHBN_SCORE | 0.20 | 1.00 |
| TPSA_SCORE | 61.77 | 0.57 |
| PKA_SCORE | 8.81 | 1.00 |
| BBB_SCORE | | 4.40 |

ARO_R_SCORE = Number of aromatic rings; HA_SCORE = Number of heavy atoms. MWHNM_SCORE = [-0.5MW(HBA+HBD)] where MW = molecular weight, HBA = number of hydrogen bond acceptor sites, and HBD = number of hydrogen bond donors; TPSA = topological polar surface area; PKA_SCORE = Acidity strength. Gupta, M.; Lee, H. J.; Barden, C. J.; Weaver, D. “The Blood-Brain Barrier (BBB) Score” J. Med. Chem. 2019, 62 (21), 9824-9836.

| Calc. | MarvinSketch | | IP | RB | HLB | logS | PL | MPA | MSA | HBA | MR | hERG_AM_ | hERG_CM_ |
| --- | --- | --- | --- | --- | --- | --- | --- | --- | --- | --- | --- | --- | --- |
|  |  | T_0_ | NA | 5 | 14.79 | -3.40 | 55.93 | 73.05 | 781.41 | 24 | 139.02 | 4.94 | safe |
| ClogP | 1.16 | 1.00 |  |  |  |  |  |  |  |  |  |  |  |
| ClogD | 1.16 | 1.00 |  |  |  |  |  |  |  |  |  |  |  |
| MW | 588.56 | 0.00 |  |  |  |  |  |  |  |  |  |  |  |
| PSA | 160.83 | 0.00 |  |  |  |  |  |  |  |  |  |  |  |
| HBD | 3.00 | 0.25 |  |  |  |  |  |  |  |  |  |  |  |
| pKa | -3.69 | 1.00 |  |  |  |  |  |  |  |  |  |  |  |
| MPO | 3.25 | |  |  |  |  |  |  |  |  |  |  |  |
| BBB | 1.74 | |  |  |  |  |  |  |  |  |  |  |  |
| LogBB | -2.07 | |  |  |  |  |  |  |  |  |  |  |  |

IP = Isoelectronic Point; RB = Number of rotatable bonds; HLB = hydrophilic-lipophilic balance; logS = water solubility; PL = polarizability(Å^3^); Minimal Projection Area (Å^2^); MSA = molecular surface area; HBA = hydrogen bond acceptors; MR = Molar refractivity; ClogD = distribution at pH = 7.4; PSA = polar surface area; HBD = hydrogen bond donors; pKa = estimated acid strength; MPO = score for CNS penetration; LogBB = blood-brain distribution (0.152ClogP – 0.0148PSA + 0.138); hERG = estimated pIC50 (pAct) value for hERG (the human ether-a-go-go (hERG) ion channel); hERG_AM_ = hERG activity model; hERG_CM_ = hERG classification model

| BBB Score Calculator | | |
| --- | --- | --- |
| Property | Value | T_0_ |
| ARO_R_SCORE | 2 | 1.00 |
| HA_SCORE | 42 | 0.24 |
| HWHBN_SCORE | 0.62 | 0.00 |
| TPSA_SCORE | 160.83 | 0.00 |
| PKA_SCORE | 8.81 | 1.00 |
| BBB_SCORE | | 1.74 |

ARO_R_SCORE = Number of aromatic rings; HA_SCORE = Number of heavy atoms. MWHNM_SCORE = [-0.5MW(HBA+HBD)] where MW = molecular weight, HBA = number of hydrogen bond acceptor sites, and HBD = number of hydrogen bond donors; TPSA = topological polar surface area; PKA_SCORE = Acidity strength. Gupta, M.; Lee, H. J.; Barden, C. J.; Weaver, D. “The Blood-Brain Barrier (BBB) Score” J. Med. Chem. 2019, 62 (21), 9824-9836.

| Calc. | MarvinSketch | | IP | RB | HLB | logS | PL | MPA | MSA | HBA | MR | hERG_AM_ | hERG_CM_ |
| --- | --- | --- | --- | --- | --- | --- | --- | --- | --- | --- | --- | --- | --- |
|  |  | T_0_ | 5.95 | 4 | 12.29 | -4.07 | 35.63 | 44.34 | 459.71 | 4 | 88.66 | 5.06 | safe |
| ClogP | 1.93 | 1.00 |  |  |  |  |  |  |  |  |  |  |  |
| ClogD | 1.93 | 1.00 |  |  |  |  |  |  |  |  |  |  |  |
| MW | 345.35 | 1.00 |  |  |  |  |  |  |  |  |  |  |  |
| PSA | 57.50 | 1.00 |  |  |  |  |  |  |  |  |  |  |  |
| HBD | 2.00 | 0.50 |  |  |  |  |  |  |  |  |  |  |  |
| pKa | -1.85 | 1.00 |  |  |  |  |  |  |  |  |  |  |  |
| MPO | 5.50 | |  |  |  |  |  |  |  |  |  |  |  |
| BBB | 4.81 | |  |  |  |  |  |  |  |  |  |  |  |
| LogBB | -0.42 | |  |  |  |  |  |  |  |  |  |  |  |

IP = Isoelectronic Point; RB = Number of rotatable bonds; HLB = hydrophilic-lipophilic balance; logS = water solubility; PL = polarizability(Å^3^); Minimal Projection Area (Å^2^); MSA = molecular surface area; HBA = hydrogen bond acceptors; MR = Molar refractivity; ClogD = distribution at pH = 7.4; PSA = polar surface area; HBD = hydrogen bond donors; pKa = estimated acid strength; MPO = score for CNS penetration; LogBB = blood-brain distribution (0.152ClogP – 0.0148PSA + 0.138); hERG = estimated pIC50 (pAct) value for hERG (the human ether-a-go-go (hERG) ion channel); hERG_AM_ = hERG activity model; hERG_CM_ = hERG classification model

| BBB Score Calculator | | |
| --- | --- | --- |
| Property | Value | T_0_ |
| ARO_R_SCORE | 3.00 | 0.69 |
| HA_SCORE | 25.00 | 0.52 |
| HWHBN_SCORE | 0.22 | 1.00 |
| TPSA_SCORE | 57.50 | 0.60 |
| PKA_SCORE | 8.81 | 1.00 |
| BBB_SCORE | | 4.81 |

ARO_R_SCORE = Number of aromatic rings; HA_SCORE = Number of heavy atoms. MWHNM_SCORE = [-0.5MW(HBA+HBD)] where MW = molecular weight, HBA = number of hydrogen bond acceptor sites, and HBD = number of hydrogen bond donors; TPSA = topological polar surface area; PKA_SCORE = Acidity strength. Gupta, M.; Lee, H. J.; Barden, C. J.; Weaver, D. “The Blood-Brain Barrier (BBB) Score” J. Med. Chem. 2019, 62 (21), 9824-9836.

| Calc. | MarvinSketch | | IP | RB | HLB | logS | PL | MPA | MSA | HBA | MR | hERG_AM_ | hERG_CM_ |
| --- | --- | --- | --- | --- | --- | --- | --- | --- | --- | --- | --- | --- | --- |
|  |  | T_0_ | 10.51 | 5 | 17.01 | -3.47 | 63.01 | 67.37 | 841.25 | 10 | 161.33 | 5.49 | toxic |
| ClogP | 2.78 | 1.00 |  |  |  |  |  |  |  |  |  |  |  |
| ClogD | 1.00 | 1.00 |  |  |  |  |  |  |  |  |  |  |  |
| MW | 586.69 | 0.00 |  |  |  |  |  |  |  |  |  |  |  |
| PSA | 112.51 | 0.25 |  |  |  |  |  |  |  |  |  |  |  |
| HBD | 1.00 | 0.75 |  |  |  |  |  |  |  |  |  |  |  |
| pKa | 9.17 | 0.41 |  |  |  |  |  |  |  |  |  |  |  |
| MPO | 3.41 | |  |  |  |  |  |  |  |  |  |  |  |
| BBB | 3.13 | |  |  |  |  |  |  |  |  |  |  |  |
| LogBB | -1.10 | |  |  |  |  |  |  |  |  |  |  |  |

IP = Isoelectronic Point; RB = Number of rotatable bonds; HLB = hydrophilic-lipophilic balance; logS = water solubility; PL = polarizability(Å^3^); Minimal Projection Area (Å^2^); MSA = molecular surface area; HBA = hydrogen bond acceptors; MR = Molar refractivity; ClogD = distribution at pH = 7.4; PSA = polar surface area; HBD = hydrogen bond donors; pKa = estimated acid strength; MPO = score for CNS penetration; LogBB = blood-brain distribution (0.152ClogP – 0.0148PSA + 0.138); hERG = estimated pIC50 (pAct) value for hERG (the human ether-a-go-go (hERG) ion channel); hERG_AM_ = hERG activity model; hERG_CM_ = hERG classification model

| BBB Score Calculator | | |
| --- | --- | --- |
| Property | Value | T_0_ |
| ARO_R_SCORE | 3 | 0.69 |
| HA_SCORE | 43 | 0.21 |
| HWHBN_SCORE | 0.29 | 0.87 |
| TPSA_SCORE | 112.51 | 0.21 |
| PKA_SCORE | 9.17 | 0.99 |
| BBB_SCORE | | 3.13 |

ARO_R_SCORE = Number of aromatic rings; HA_SCORE = Number of heavy atoms. MWHNM_SCORE = [-0.5MW(HBA+HBD)] where MW = molecular weight, HBA = number of hydrogen bond acceptor sites, and HBD = number of hydrogen bond donors; TPSA = topological polar surface area; PKA_SCORE = Acidity strength. Gupta, M.; Lee, H. J.; Barden, C. J.; Weaver, D. “The Blood-Brain Barrier (BBB) Score” J. Med. Chem. 2019, 62 (21), 9824-9836.

| Calc. | MarvinSketch | | IP | RB | HLB | logS | PL | MPA | MSA | HBA | MR | hERG_AM_ | hERG_CM_ |
| --- | --- | --- | --- | --- | --- | --- | --- | --- | --- | --- | --- | --- | --- |
|  |  | T_0_ | 3.16 | 9 | 15.30 | 0.00 | 43.52 | 55.08 | 601.64 | 17 | 119.21 | 4.71 | safe |
| ClogP | -1.63 | 1.00 |  |  |  |  |  |  |  |  |  |  |  |
| ClogD | -5.70 | 1.00 |  |  |  |  |  |  |  |  |  |  |  |
| MW | 454.45 | 0.33 |  |  |  |  |  |  |  |  |  |  |  |
| PSA | 210.54 | 0.00 |  |  |  |  |  |  |  |  |  |  |  |
| HBD | 7.00 | 0.00 |  |  |  |  |  |  |  |  |  |  |  |
| pKa | 5.57 | 1.00 |  |  |  |  |  |  |  |  |  |  |  |
| MPO | 3.33 | |  |  |  |  |  |  |  |  |  |  |  |
| BBB | 1.55 | |  |  |  |  |  |  |  |  |  |  |  |
| LogBB | -3.04 | |  |  |  |  |  |  |  |  |  |  |  |

IP = Isoelectronic Point; RB = Number of rotatable bonds; HLB = hydrophilic-lipophilic balance; logS = water solubility; PL = polarizability(Å^3^); Minimal Projection Area (Å^2^); MSA = molecular surface area; HBA = hydrogen bond acceptors; MR = Molar refractivity; ClogD = distribution at pH = 7.4; PSA = polar surface area; HBD = hydrogen bond donors; pKa = estimated acid strength; MPO = score for CNS penetration; LogBB = blood-brain distribution (0.152ClogP – 0.0148PSA + 0.138); hERG = estimated pIC50 (pAct) value for hERG (the human ether-a-go-go (hERG) ion channel); hERG_AM_ = hERG activity model; hERG_CM_ = hERG classification model

| BBB Score Calculator | | |
| --- | --- | --- |
| Property | Value | T_0_ |
| ARO_R_SCORE | 3 | 0.69 |
| HA_SCORE | 33 | 0.61 |
| HWHBN_SCORE | 0.80 | 0.00 |
| TPSA_SCORE | 210.54 | 0.00 |
| PKA_SCORE | 5.57 | 0.48 |
| BBB_SCORE | | 1.55 |

ARO_R_SCORE = Number of aromatic rings; HA_SCORE = Number of heavy atoms. MWHNM_SCORE = [-0.5MW(HBA+HBD)] where MW = molecular weight, HBA = number of hydrogen bond acceptor sites, and HBD = number of hydrogen bond donors; TPSA = topological polar surface area; PKA_SCORE = Acidity strength. Gupta, M.; Lee, H. J.; Barden, C. J.; Weaver, D. “The Blood-Brain Barrier (BBB) Score” J. Med. Chem. 2019, 62 (21), 9824-9836.

| Calc. | MarvinSketch | | IP | RB | HLB | logS | PL | MPA | MSA | HBA | MR | hERG_AM_ | hERG_CM_ |
| --- | --- | --- | --- | --- | --- | --- | --- | --- | --- | --- | --- | --- | --- |
|  |  | T_0_ | 11.66 | 5 | 22.85 | -1.02 | 25.22 | 38.17 | 376.41 | 4 | 86.98 | 4.84 | Safe |
| ClogP | 0.99 | 1.00 |  |  |  |  |  |  |  |  |  |  |  |
| ClogD | -0.01 | 1.00 |  |  |  |  |  |  |  |  |  |  |  |
| MW | 221.30 | 1.00 |  |  |  |  |  |  |  |  |  |  |  |
| PSA | 53.16 | 1.00 |  |  |  |  |  |  |  |  |  |  |  |
| HBD | 3.00 | 0.25 |  |  |  |  |  |  |  |  |  |  |  |
| pKa | 8.36 | 0.82 |  |  |  |  |  |  |  |  |  |  |  |
| MPO | 5.07 | |  |  |  |  |  |  |  |  |  |  |  |
| BBB | 4.22 | |  |  |  |  |  |  |  |  |  |  |  |
| LogBB | -0.49 | |  |  |  |  |  |  |  |  |  |  |  |

IP = Isoelectronic Point; RB = Number of rotatable bonds; HLB = hydrophilic-lipophilic balance; logS = water solubility; PL = polarizability(Å^3^); Minimal Projection Area (Å^2^); MSA = molecular surface area; HBA = hydrogen bond acceptors; MR = Molar refractivity; ClogD = distribution at pH = 7.4; PSA = polar surface area; HBD = hydrogen bond donors; pKa = estimated acid strength; MPO = score for CNS penetration; LogBB = blood-brain distribution (0.152ClogP – 0.0148PSA + 0.138); hERG = estimated pIC50 (pAct) value for hERG (the human ether-a-go-go (hERG) ion channel); hERG_AM_ = hERG activity model; hERG_CM_ = hERG classification model

| BBB Score Calculator | | |
| --- | --- | --- |
| Property | Value | T_0_ |
| ARO_R_SCORE | 1 | 0.82 |
| HA_SCORE | 16 | 0.95 |
| HWHBN_SCORE | 0.40 | 0.46 |
| TPSA_SCORE | 53.16 | 0.63 |
| PKA_SCORE | 8.36 | 0.99 |
| BBB_SCORE | | 4.22 |

ARO_R_SCORE = Number of aromatic rings; HA_SCORE = Number of heavy atoms. MWHNM_SCORE = [-0.5MW(HBA+HBD)] where MW = molecular weight, HBA = number of hydrogen bond acceptor sites, and HBD = number of hydrogen bond donors; TPSA = topological polar surface area; PKA_SCORE = Acidity strength. Gupta, M.; Lee, H. J.; Barden, C. J.; Weaver, D. “The Blood-Brain Barrier (BBB) Score” J. Med. Chem. 2019, 62 (21), 9824-9836.

| Calc. | MarvinSketch | | IP | RB | HLB | logS | PL | MPA | MSA | HBA | MR | hERG_AM_ | hERG_CM_ |
| --- | --- | --- | --- | --- | --- | --- | --- | --- | --- | --- | --- | --- | --- |
|  |  | T_0_ | NA | 5 | 13.24 | -1.27 | 23.02 | 44.34 | 355.40 | 2 | 58.48 | 3.32 | safe |
| ClogP | 0.10 | 1.00 |  |  |  |  |  |  |  |  |  |  |  |
| ClogD | 0.10 | 1.00 |  |  |  |  |  |  |  |  |  |  |  |
| MW | 261.08 | 1.00 |  |  |  |  |  |  |  |  |  |  |  |
| PSA | 41.57 | 1.00 |  |  |  |  |  |  |  |  |  |  |  |
| HBD | 1.00 | 0.75 |  |  |  |  |  |  |  |  |  |  |  |
| pKa | 0.00 | 1.00 |  |  |  |  |  |  |  |  |  |  |  |
| MPO | 5.75 | |  |  |  |  |  |  |  |  |  |  |  |
| BBB | 4.41 | |  |  |  |  |  |  |  |  |  |  |  |
| LogBB | -0.46 | |  |  |  |  |  |  |  |  |  |  |  |

IP = Isoelectronic Point; RB = Number of rotatable bonds; HLB = hydrophilic-lipophilic balance; logS = water solubility; PL = polarizability(Å^3^); Minimal Projection Area (Å^2^); MSA = molecular surface area; HBA = hydrogen bond acceptors; MR = Molar refractivity; ClogD = distribution at pH = 7.4; PSA = polar surface area; HBD = hydrogen bond donors; pKa = estimated acid strength; MPO = score for CNS penetration; LogBB = blood-brain distribution (0.152ClogP – 0.0148PSA + 0.138); hERG = estimated pIC50 (pAct) value for hERG (the human ether-a-go-go (hERG) ion channel); hERG_AM_ = hERG activity model; hERG_CM_ = hERG classification model

| BBB Score Calculator | | |
| --- | --- | --- |
| Property | Value | T_0_ |
| ARO_R_SCORE | 0 | 0.34 |
| HA_SCORE | 14 | 0.89 |
| HWHBN_SCORE | 0.12 | 0.85 |
| TPSA_SCORE | 41.57 | 0.71 |
| PKA_SCORE | 8.81 | 1.00 |
| BBB_SCORE | | 4.41 |

ARO_R_SCORE = Number of aromatic rings; HA_SCORE = Number of heavy atoms. MWHNM_SCORE = [-0.5MW(HBA+HBD)] where MW = molecular weight, HBA = number of hydrogen bond acceptor sites, and HBD = number of hydrogen bond donors; TPSA = topological polar surface area; PKA_SCORE = Acidity strength. Gupta, M.; Lee, H. J.; Barden, C. J.; Weaver, D. “The Blood-Brain Barrier (BBB) Score” J. Med. Chem. 2019, 62 (21), 9824-9836.

| Calc. | MarvinSketch | | IP | RB | HLB | logS | PL | MPA | MSA | HBA | MR | hERG_AM_ | hERG_CM_ |
| --- | --- | --- | --- | --- | --- | --- | --- | --- | --- | --- | --- | --- | --- |
|  |  | T_0_ | 9.81 | 10 | 22.96 | -2.04 | 87.84 | 99.55 | NA | 16 | 221.48 | 5.16 | toxic |
| ClogP | 3.13 | 0.94 |  |  |  |  |  |  |  |  |  |  |  |
| ClogD | 1.54 | 1.00 |  |  |  |  |  |  |  |  |  |  |  |
| MW | 824.97 | 0.00 |  |  |  |  |  |  |  |  |  |  |  |
| PSA | 171.17 | 0.00 |  |  |  |  |  |  |  |  |  |  |  |
| HBD | 3.00 | 0.25 |  |  |  |  |  |  |  |  |  |  |  |
| pKa | 8.42 | 0.79 |  |  |  |  |  |  |  |  |  |  |  |
| MPO | 2.97 | |  |  |  |  |  |  |  |  |  |  |  |
| BBB | 1.80 | |  |  |  |  |  |  |  |  |  |  |  |
| LogBB | -1.92 | |  |  |  |  |  |  |  |  |  |  |  |

IP = Isoelectronic Point; RB = Number of rotatable bonds; HLB = hydrophilic-lipophilic balance; logS = water solubility; PL = polarizability(Å^3^); Minimal Projection Area (Å^2^); MSA = molecular surface area; HBA = hydrogen bond acceptors; MR = Molar refractivity; ClogD = distribution at pH = 7.4; PSA = polar surface area; HBD = hydrogen bond donors; pKa = estimated acid strength; MPO = score for CNS penetration; LogBB = blood-brain distribution (0.152ClogP – 0.0148PSA + 0.138); hERG = estimated pIC50 (pAct) value for hERG (the human ether-a-go-go (hERG) ion channel); hERG_AM_ = hERG activity model; hERG_CM_ = hERG classification model

| BBB Score Calculator | | |
| --- | --- | --- |
| Property | Value | T_0_ |
| ARO_R_SCORE | 3 | 0.69 |
| HA_SCORE | 60 | 0.00 |
| HWHBN_SCORE | 0.42 | 0.41 |
| TPSA_SCORE | 171.17 | 0.00 |
| PKA_SCORE | 8.42 | 0.99 |
| BBB_SCORE | | 1.80 |

ARO_R_SCORE = Number of aromatic rings; HA_SCORE = Number of heavy atoms. MWHNM_SCORE = [-0.5MW(HBA+HBD)] where MW = molecular weight, HBA = number of hydrogen bond acceptor sites, and HBD = number of hydrogen bond donors; TPSA = topological polar surface area; PKA_SCORE = Acidity strength. Gupta, M.; Lee, H. J.; Barden, C. J.; Weaver, D. “The Blood-Brain Barrier (BBB) Score” J. Med. Chem. 2019, 62 (21), 9824-9836.

| Calc. | MarvinSketch | | IP | RB | HLB | logS | PL | MPA | MSA | HBA | MR | hERG_AM_ | hERG_CM_ |
| --- | --- | --- | --- | --- | --- | --- | --- | --- | --- | --- | --- | --- | --- |
|  |  | T_0_ | NA | 6 | 2.88 | -7.07 | 42.67 | 45.91 | 551.01 | 6 | 111.47 | 5.30 | safe |
| ClogP | 5.79 | 0.00 |  |  |  |  |  |  |  |  |  |  |  |
| ClogD | 5.79 | 0.00 |  |  |  |  |  |  |  |  |  |  |  |
| MW | 393.87 | 0.76 |  |  |  |  |  |  |  |  |  |  |  |
| PSA | 55.40 | 1.00 |  |  |  |  |  |  |  |  |  |  |  |
| HBD | 1.00 | 0.75 |  |  |  |  |  |  |  |  |  |  |  |
| pKa | -4.93 | 1.00 |  |  |  |  |  |  |  |  |  |  |  |
| MPO | 3.51 | |  |  |  |  |  |  |  |  |  |  |  |
| BBB | 4.74 | |  |  |  |  |  |  |  |  |  |  |  |
| LogBB | 0.20 | |  |  |  |  |  |  |  |  |  |  |  |

IP = Isoelectronic Point; RB = Number of rotatable bonds; HLB = hydrophilic-lipophilic balance; logS = water solubility; PL = polarizability(Å^3^); Minimal Projection Area (Å^2^); MSA = molecular surface area; HBA = hydrogen bond acceptors; MR = Molar refractivity; ClogD = distribution at pH = 7.4; PSA = polar surface area; HBD = hydrogen bond donors; pKa = estimated acid strength; MPO = score for CNS penetration; LogBB = blood-brain distribution (0.152ClogP – 0.0148PSA + 0.138); hERG = estimated pIC50 (pAct) value for hERG (the human ether-a-go-go (hERG) ion channel); hERG_AM_ = hERG activity model; hERG_CM_ = hERG classification model

| BBB Score Calculator | | |
| --- | --- | --- |
| Property | Value | T_0_ |
| ARO_R_SCORE | 3 | 0.69 |
| HA_SCORE | 28 | 0.82 |
| HWHBN_SCORE | 0.20 | 1.00 |
| TPSA_SCORE | 55.40 | 0.61 |
| PKA_SCORE | 8.81 | 1.00 |
| BBB_SCORE | | 4.74 |

ARO_R_SCORE = Number of aromatic rings; HA_SCORE = Number of heavy atoms. MWHNM_SCORE = [-0.5MW(HBA+HBD)] where MW = molecular weight, HBA = number of hydrogen bond acceptor sites, and HBD = number of hydrogen bond donors; TPSA = topological polar surface area; PKA_SCORE = Acidity strength. Gupta, M.; Lee, H. J.; Barden, C. J.; Weaver, D. “The Blood-Brain Barrier (BBB) Score” J. Med. Chem. 2019, 62 (21), 9824-9836.

| Calc. | MarvinSketch | | IP | RB | HLB | logS | PL | MPA | MSA | HBA | MR | hERG_AM_ | hERG_CM_ |
| --- | --- | --- | --- | --- | --- | --- | --- | --- | --- | --- | --- | --- | --- |
|  |  | T0 | 7.57 | 6 | 3.99 | -6.45 | 41.78 | 49.64 | 543.63 | 7 | 109.63 | 5.19 | safe |
| ClogP | 5.17 | 0.00 |  |  |  |  |  |  |  |  |  |  |  |
| ClogD | 5.17 | 0.00 |  |  |  |  |  |  |  |  |  |  |  |
| MW | 394.86 | 0.75 |  |  |  |  |  |  |  |  |  |  |  |
| PSA | 68.29 | 1.00 |  |  |  |  |  |  |  |  |  |  |  |
| HBD | 1.00 | 0.75 |  |  |  |  |  |  |  |  |  |  |  |
| pKa | 3.99 | 1.00 |  |  |  |  |  |  |  |  |  |  |  |
| MPO | 3.50 | |  |  |  |  |  |  |  |  |  |  |  |
| BBB | 4.50 | |  |  |  |  |  |  |  |  |  |  |  |
| LogBB | -0.09 | |  |  |  |  |  |  |  |  |  |  |  |

IP = Isoelectronic Point; RB = Number of rotatable bonds; HLB = hydrophilic-lipophilic balance; logS = water solubility; PL = polarizability(Å^3^); Minimal Projection Area (Å^2^); MSA = molecular surface area; HBA = hydrogen bond acceptors; MR = Molar refractivity; ClogD = distribution at pKa; PSA = polar surface area; HBD = hydrogen bond donors; pKa = estimated acid strength; MPO = score for CNS penetration; LogBB = blood-brain distribution (0.152ClogP – 0.0148PSA + 0.138); hERG = estimated pIC50 (pAct) value for hERG (the human ether-a-go-go (hERG) ion channel); hERG_AM_ = hERG activity model; hERG_CM_ = hERG classification model

| BBB Score Calculator | | |
| --- | --- | --- |
| Property | Value | T_0_ |
| ARO_R_SCORE | 3 | 0.69 |
| HA_SCORE | 28 | 0.82 |
| HWHBN_SCORE | 0.25 | 0.96 |
| TPSA_SCORE | 68.29 | 0.52 |
| PKA_SCORE | 8.81 | 1.00 |
| BBB_SCORE | | 4.50 |

ARO_R_SCORE = Number of aromatic rings; HA_SCORE = Number of heavy atoms. MWHNM_SCORE = [-0.5MW(HBA+HBD)] where MW = molecular weight, HBA = number of hydrogen bond acceptor sites, and HBD = number of hydrogen bond donors; TPSA = topological polar surface area; PKA_SCORE = Acidity strength. Gupta, M.; Lee, H. J.; Barden, C. J.; Weaver, D. “The Blood-Brain Barrier (BBB) Score” J. Med. Chem. 2019, 62 (21), 9824-9836.

| Calc. | MarvinSketch | | IP | RB | HLB | logS | PL | MPA | MSA | HBA | MR | hERG_AM_ | hERG_CM_ |
| --- | --- | --- | --- | --- | --- | --- | --- | --- | --- | --- | --- | --- | --- |
|  |  | T0 | 8.39 | 6 | 4.54 | -5.89 | 41.78 | 41.15 | 543.37 | 7 | 109.31 | 5.28 | safe |
| ClogP | 4.58 | 0.21 |  |  |  |  |  |  |  |  |  |  |  |
| ClogD | 4.57 | 0.00 |  |  |  |  |  |  |  |  |  |  |  |
| MW | 394.86 | 0.75 |  |  |  |  |  |  |  |  |  |  |  |
| PSA | 68.29 | 1.00 |  |  |  |  |  |  |  |  |  |  |  |
| HBD | 1.00 | 0.75 |  |  |  |  |  |  |  |  |  |  |  |
| pKa | 4.37 | 1.00 |  |  |  |  |  |  |  |  |  |  |  |
| MPO | 3.71 | |  |  |  |  |  |  |  |  |  |  |  |
| BBB | 4.50 | |  |  |  |  |  |  |  |  |  |  |  |
| LogBB | -0.18 | |  |  |  |  |  |  |  |  |  |  |  |

IP = Isoelectronic Point; RB = Number of rotatable bonds; HLB = hydrophilic-lipophilic balance; logS = water solubility; PL = polarizability(Å^3^); Minimal Projection Area (Å^2^); MSA = molecular surface area; HBA = hydrogen bond acceptors; MR = Molar refractivity; ClogD = distribution at pKa; PSA = polar surface area; HBD = hydrogen bond donors; pKa = estimated acid strength; MPO = score for CNS penetration; LogBB = blood-brain distribution (0.152ClogP – 0.0148PSA + 0.138); hERG = estimated pIC50 (pAct) value for hERG (the human ether-a-go-go (hERG) ion channel); hERG_AM_ = hERG activity model; hERG_CM_ = hERG classification model

| BBB Score Calculator | | |
| --- | --- | --- |
| Property | Value | T_0_ |
| ARO_R_SCORE | 3 | 0.69 |
| HA_SCORE | 28 | 0.82 |
| HWHBN_SCORE | 0.25 | 0.96 |
| TPSA_SCORE | 68.29 | 0.52 |
| PKA_SCORE | 8.81 | 1.00 |
| BBB_SCORE | | 4.50 |

ARO_R_SCORE = Number of aromatic rings; HA_SCORE = Number of heavy atoms. MWHNM_SCORE = [-0.5MW(HBA+HBD)] where MW = molecular weight, HBA = number of hydrogen bond acceptor sites, and HBD = number of hydrogen bond donors; TPSA = topological polar surface area; PKA_SCORE = Acidity strength. Gupta, M.; Lee, H. J.; Barden, C. J.; Weaver, D. “The Blood-Brain Barrier (BBB) Score” J. Med. Chem. 2019, 62 (21), 9824-9836.

| Calc. | MarvinSketch | | IP | RB | HLB | logS | PL | MPA | MSA | HBA | MR | hERG_AM_ | hERG_CM_ |
| --- | --- | --- | --- | --- | --- | --- | --- | --- | --- | --- | --- | --- | --- |
|  |  | T0 | 8.96 | 6 | 4.54 | -5.89 | 41.78 | 44.70 | 453.10 | 7 | 109.31 | 5.28 | Safe |
| ClogP | 4.58 | 0.21 |  |  |  |  |  |  |  |  |  |  |  |
| ClogD | 4.57 | 0.00 |  |  |  |  |  |  |  |  |  |  |  |
| MW | 394.86 | 0.75 |  |  |  |  |  |  |  |  |  |  |  |
| PSA | 68.29 | 1.00 |  |  |  |  |  |  |  |  |  |  |  |
| HBD | 1.00 | 0.75 |  |  |  |  |  |  |  |  |  |  |  |
| pKa | 4.85 | 1.00 |  |  |  |  |  |  |  |  |  |  |  |
| MPO | 3.71 | |  |  |  |  |  |  |  |  |  |  |  |
| BBB | 4.50 | |  |  |  |  |  |  |  |  |  |  |  |
| LogBB | -0.18 | |  |  |  |  |  |  |  |  |  |  |  |

IP = Isoelectronic Point; RB = Number of rotatable bonds; HLB = hydrophilic-lipophilic balance; logS = water solubility; PL = polarizability(Å^3^); Minimal Projection Area (Å^2^); MSA = molecular surface area; HBA = hydrogen bond acceptors; MR = Molar refractivity; ClogD = distribution at pKa; PSA = polar surface area; HBD = hydrogen bond donors; pKa = estimated acid strength; MPO = score for CNS penetration; LogBB = blood-brain distribution (0.152ClogP – 0.0148PSA + 0.138); hERG = estimated pIC50 (pAct) value for hERG (the human ether-a-go-go (hERG) ion channel); hERG_AM_ = hERG activity model; hERG_CM_ = hERG classification model

| BBB Score Calculator | | |
| --- | --- | --- |
| Property | Value | T_0_ |
| ARO_R_SCORE | 3 | 0.69 |
| HA_SCORE | 28 | 0.82 |
| HWHBN_SCORE | 0.25 | 0.96 |
| TPSA_SCORE | 68.29 | 0.52 |
| PKA_SCORE | 8.81 | 1.00 |
| BBB_SCORE | | 4.50 |

ARO_R_SCORE = Number of aromatic rings; HA_SCORE = Number of heavy atoms. MWHNM_SCORE = [-0.5MW(HBA+HBD)] where MW = molecular weight, HBA = number of hydrogen bond acceptor sites, and HBD = number of hydrogen bond donors; TPSA = topological polar surface area; PKA_SCORE = Acidity strength. Gupta, M.; Lee, H. J.; Barden, C. J.; Weaver, D. “The Blood-Brain Barrier (BBB) Score” J. Med. Chem. 2019, 62 (21), 9824-9836.

| Calc. | MarvinSketch | | IP | RB | HLB | logS | PL | MPA | MSA | HBA | MR | hERG_AM_ | hERG_CM_ |
| --- | --- | --- | --- | --- | --- | --- | --- | --- | --- | --- | --- | --- | --- |
|  |  | T0 | NA | 6 | 4.69 | -5.97 | 43.62 | 50.87 | 575.89 | 7 | 112.43 | 5.44 | safe |
| ClogP | 4.44 | 0.28 |  |  |  |  |  |  |  |  |  |  |  |
| ClogD | 4.44 | 0.00 |  |  |  |  |  |  |  |  |  |  |  |
| MW | 408.88 | 0.65 |  |  |  |  |  |  |  |  |  |  |  |
| PSA | 59.50 | 1.00 |  |  |  |  |  |  |  |  |  |  |  |
| HBD | 0.00 | 1.00 |  |  |  |  |  |  |  |  |  |  |  |
| pKa | 4.33 | 1.00 |  |  |  |  |  |  |  |  |  |  |  |
| MPO | 3.93 | |  |  |  |  |  |  |  |  |  |  |  |
| BBB | 4.65 | |  |  |  |  |  |  |  |  |  |  |  |
| LogBB | -0.07 | |  |  |  |  |  |  |  |  |  |  |  |

IP = Isoelectronic Point; RB = Number of rotatable bonds; HLB = hydrophilic-lipophilic balance; logS = water solubility; PL = polarizability(Å^3^); Minimal Projection Area (Å^2^); MSA = molecular surface area; HBA = hydrogen bond acceptors; MR = Molar refractivity; ClogD = distribution at pKa; PSA = polar surface area; HBD = hydrogen bond donors; pKa = estimated acid strength; MPO = score for CNS penetration; LogBB = blood-brain distribution (0.152ClogP – 0.0148PSA + 0.138); hERG = estimated pIC50 (pAct) value for hERG (the human ether-a-go-go (hERG) ion channel); hERG_AM_ = hERG activity model; hERG_CM_ = hERG classification model

| BBB Score Calculator | | |
| --- | --- | --- |
| Property | Value | T_0_ |
| ARO_R_SCORE | 3 | 0.69 |
| HA_SCORE | 29 | 0.79 |
| HWHBN_SCORE | 0.20 | 1.00 |
| TPSA_SCORE | 59.50 | 0.58 |
| PKA_SCORE | 8.81 | 1.00 |
| BBB_SCORE | | 4.65 |

ARO_R_SCORE = Number of aromatic rings; HA_SCORE = Number of heavy atoms. MWHNM_SCORE = [-0.5MW(HBA+HBD)] where MW = molecular weight, HBA = number of hydrogen bond acceptor sites, and HBD = number of hydrogen bond donors; TPSA = topological polar surface area; PKA_SCORE = Acidity strength. Gupta, M.; Lee, H. J.; Barden, C. J.; Weaver, D. “The Blood-Brain Barrier (BBB) Score” J. Med. Chem. 2019, 62 (21), 9824-9836.

| Calc. | MarvinSketch | | IP | RB | HLB | logS | PL | MPA | MSA | HBA | MR | hERG_AM_ | hERG_CM_ |
| --- | --- | --- | --- | --- | --- | --- | --- | --- | --- | --- | --- | --- | --- |
|  |  | T0 | 5.88 | 6 | 5.65 | -5.27 | 40.90 | 44.62 | 536.59 | 8 | 107.47 | 5.18 | safe |
| ClogP | 3.95 | 0.52 |  |  |  |  |  |  |  |  |  |  |  |
| ClogD | 3.95 | 0.02 |  |  |  |  |  |  |  |  |  |  |  |
| MW | 395.84 | 0.74 |  |  |  |  |  |  |  |  |  |  |  |
| PSA | 81.18 | 1.00 |  |  |  |  |  |  |  |  |  |  |  |
| HBD | 1.00 | 7.25 |  |  |  |  |  |  |  |  |  |  |  |
| pKa | 0.07 | 1.00 |  |  |  |  |  |  |  |  |  |  |  |
| MPO | 4.04 | |  |  |  |  |  |  |  |  |  |  |  |
| BBB | 4.14 | |  |  |  |  |  |  |  |  |  |  |  |
| LogBB | -0.46 | |  |  |  |  |  |  |  |  |  |  |  |

IP = Isoelectronic Point; RB = Number of rotatable bonds; HLB = hydrophilic-lipophilic balance; logS = water solubility; PL = polarizability(Å^3^); Minimal Projection Area (Å^2^); MSA = molecular surface area; HBA = hydrogen bond acceptors; MR = Molar refractivity; ClogD = distribution at pKa; PSA = polar surface area; HBD = hydrogen bond donors; pKa = estimated acid strength; MPO = score for CNS penetration; LogBB = blood-brain distribution (0.152ClogP – 0.0148PSA + 0.138); hERG = estimated pIC50 (pAct) value for hERG (the human ether-a-go-go (hERG) ion channel); hERG_AM_ = hERG activity model; hERG_CM_ = hERG classification model

| BBB Score Calculator | | |
| --- | --- | --- |
| Property | Value | T_0_ |
| ARO_R_SCORE | 3.00 | 0.69 |
| HA_SCORE | 28.00 | 0.82 |
| HWHBN_SCORE | 0.30 | 0.84 |
| TPSA_SCORE | 81.18 | 0.43 |
| PKA_SCORE | 8.81 | 1.00 |
| BBB_SCORE | | 4.14 |

ARO_R_SCORE = Number of aromatic rings; HA_SCORE = Number of heavy atoms. MWHNM_SCORE = [-0.5MW(HBA+HBD)] where MW = molecular weight, HBA = number of hydrogen bond acceptor sites, and HBD = number of hydrogen bond donors; TPSA = topological polar surface area; PKA_SCORE = Acidity strength. Gupta, M.; Lee, H. J.; Barden, C. J.; Weaver, D. “The Blood-Brain Barrier (BBB) Score” J. Med. Chem. 2019, 62 (21), 9824-9836.

| Calc. | MarvinSketch | | IP | RB | HLB | logS | PL | MPA | MSA | HBA | MR | hERG_AM_ | hERG_CM_ |
| --- | --- | --- | --- | --- | --- | --- | --- | --- | --- | --- | --- | --- | --- |
|  |  | T0 | NA | 7 | 3.80 | -6.92 | 44.52 | 49.64 | 582.41 | 6 | 114.52 | 5.34 | safe |
| ClogP | 5.50 | 0.00 |  |  |  |  |  |  |  |  |  |  |  |
| ClogD | 5.50 | 0.00 |  |  |  |  |  |  |  |  |  |  |  |
| MW | 407.89 | 0.66 |  |  |  |  |  |  |  |  |  |  |  |
| PSA | 55.40 | 1.00 |  |  |  |  |  |  |  |  |  |  |  |
| HBD | 1.00 | 0.75 |  |  |  |  |  |  |  |  |  |  |  |
| pKa | 2.70 | 1.00 |  |  |  |  |  |  |  |  |  |  |  |
| MPO | 3.41 | |  |  |  |  |  |  |  |  |  |  |  |
| BBB | 4.70 | |  |  |  |  |  |  |  |  |  |  |  |
| LogBB | 0.15 | |  |  |  |  |  |  |  |  |  |  |  |

IP = Isoelectronic Point; RB = Number of rotatable bonds; HLB = hydrophilic-lipophilic balance; logS = water solubility; PL = polarizability(Å^3^); Minimal Projection Area (Å^2^); MSA = molecular surface area; HBA = hydrogen bond acceptors; MR = Molar refractivity; ClogD = distribution at pKa; PSA = polar surface area; HBD = hydrogen bond donors; pKa = estimated acid strength; MPO = score for CNS penetration; LogBB = blood-brain distribution (0.152ClogP – 0.0148PSA + 0.138); hERG = estimated pIC50 (pAct) value for hERG (the human ether-a-go-go (hERG) ion channel); hERG_AM_ = hERG activity model; hERG_CM_ = hERG classification model

| BBB Score Calculator | | |
| --- | --- | --- |
| Property | Value | T_0_ |
| ARO_R_SCORE | 3 | 0.69 |
| HA_SCORE | 29.00 | 0.79 |
| HWHBN_SCORE | 0.20 | 1.00 |
| TPSA_SCORE | 55.40 | 0.61 |
| PKA_SCORE | 8.81 | 1.00 |
| BBB_SCORE | | 4.70 |

ARO_R_SCORE = Number of aromatic rings; HA_SCORE = Number of heavy atoms. MWHNM_SCORE = [-0.5MW(HBA+HBD)] where MW = molecular weight, HBA = number of hydrogen bond acceptor sites, and HBD = number of hydrogen bond donors; TPSA = topological polar surface area; PKA_SCORE = Acidity strength. Gupta, M.; Lee, H. J.; Barden, C. J.; Weaver, D. “The Blood-Brain Barrier (BBB) Score” J. Med. Chem. 2019, 62 (21), 9824-9836.

| Calc. | MarvinSketch | | IP | RB | HLB | logS | PL | MPA | MSA | HBA | MR | hERG_AM_ | hERG_CM_ |
| --- | --- | --- | --- | --- | --- | --- | --- | --- | --- | --- | --- | --- | --- |
|  |  | T0 | 8.23 | 7 | 5.41 | -5.97 | 43.63 | 53.16 | 575.97 | 4 | 111.85 | 5.28 | safe |
| ClogP | 4.36 | 0.32 |  |  |  |  |  |  |  |  |  |  |  |
| ClogD | 4.36 | 0.00 |  |  |  |  |  |  |  |  |  |  |  |
| MW | 408.88 | 0.65 |  |  |  |  |  |  |  |  |  |  |  |
| PSA | 68.29 | 1.00 |  |  |  |  |  |  |  |  |  |  |  |
| HBD | 1.00 | 0.75 |  |  |  |  |  |  |  |  |  |  |  |
| pKa | 4.16 | 1.00 |  |  |  |  |  |  |  |  |  |  |  |
| MPO | 3.72 | |  |  |  |  |  |  |  |  |  |  |  |
| BBB | 4.47 | |  |  |  |  |  |  |  |  |  |  |  |
| LogBB | -0.21 | |  |  |  |  |  |  |  |  |  |  |  |

IP = Isoelectronic Point; RB = Number of rotatable bonds; HLB = hydrophilic-lipophilic balance; logS = water solubility; PL = polarizability(Å^3^); Minimal Projection Area (Å^2^); MSA = molecular surface area; HBA = hydrogen bond acceptors; MR = Molar refractivity; ClogD = distribution at pKa; PSA = polar surface area; HBD = hydrogen bond donors; pKa = estimated acid strength; MPO = score for CNS penetration; LogBB = blood-brain distribution (0.152ClogP – 0.0148PSA + 0.138); hERG = estimated pIC50 (pAct) value for hERG (the human ether-a-go-go (hERG) ion channel); hERG_AM_ = hERG activity model; hERG_CM_ = hERG classification model

| BBB Score Calculator | | |
| --- | --- | --- |
| Property | Value | T_0_ |
| ARO_R_SCORE | 3 | 0.69 |
| HA_SCORE | 29 | 0.79 |
| HWHBN_SCORE | 0.25 | 0.96 |
| TPSA_SCORE | 68.29 | 0.52 |
| PKA_SCORE | 8.81 | 1.00 |
| BBB_SCORE | | 4.47 |

ARO_R_SCORE = Number of aromatic rings; HA_SCORE = Number of heavy atoms. MWHNM_SCORE = [-0.5MW(HBA+HBD)] where MW = molecular weight, HBA = number of hydrogen bond acceptor sites, and HBD = number of hydrogen bond donors; TPSA = topological polar surface area; PKA_SCORE = Acidity strength. Gupta, M.; Lee, H. J.; Barden, C. J.; Weaver, D. “The Blood-Brain Barrier (BBB) Score” J. Med. Chem. 2019, 62 (21), 9824-9836.

| Calc. | MarvinSketch | | IP | RB | HLB | logS | PL | MPA | MSA | HBA | MR | hERG_AM_ | hERG_CM_ |
| --- | --- | --- | --- | --- | --- | --- | --- | --- | --- | --- | --- | --- | --- |
|  |  | T0 | 8.87 | 7 | 5.43 | -5.74 | 43.63 | 51.36 | 575.34 | 7 | 112.37 | 5.24 | safe |
| ClogP | 4.28 | 0.36 |  |  |  |  |  |  |  |  |  |  |  |
| ClogD | 4.28 | 0.00 |  |  |  |  |  |  |  |  |  |  |  |
| MW | 408.88 | 0.65 |  |  |  |  |  |  |  |  |  |  |  |
| PSA | 68.29 | 1.00 |  |  |  |  |  |  |  |  |  |  |  |
| HBD | 1.00 | 0.75 |  |  |  |  |  |  |  |  |  |  |  |
| pKa | 4.82 | 1.00 |  |  |  |  |  |  |  |  |  |  |  |
| MPO | 3.76 | |  |  |  |  |  |  |  |  |  |  |  |
| BBB | 4.47 | |  |  |  |  |  |  |  |  |  |  |  |
| LogBB | -0.22 | |  |  |  |  |  |  |  |  |  |  |  |

IP = Isoelectronic Point; RB = Number of rotatable bonds; HLB = hydrophilic-lipophilic balance; logS = water solubility; PL = polarizability(Å^3^); Minimal Projection Area (Å^2^); MSA = molecular surface area; HBA = hydrogen bond acceptors; MR = Molar refractivity; ClogD = distribution at pKa; PSA = polar surface area; HBD = hydrogen bond donors; pKa = estimated acid strength; MPO = score for CNS penetration; LogBB = blood-brain distribution (0.152ClogP – 0.0148PSA + 0.138); hERG = estimated pIC50 (pAct) value for hERG (the human ether-a-go-go (hERG) ion channel); hERG_AM_ = hERG activity model; hERG_CM_ = hERG classification model

| BBB Score Calculator | | |
| --- | --- | --- |
| Property | Value | T_0_ |
| ARO_R_SCORE | 3 | 0.69 |
| HA_SCORE | 29 | 0.79 |
| HWHBN_SCORE | 0.25 | 0.96 |
| TPSA_SCORE | 68.29 | 0.52 |
| PKA_SCORE | 8.81 | 1.00 |
| BBB_SCORE | | 4.47 |

ARO_R_SCORE = Number of aromatic rings; HA_SCORE = Number of heavy atoms. MWHNM_SCORE = [-0.5MW(HBA+HBD)] where MW = molecular weight, HBA = number of hydrogen bond acceptor sites, and HBD = number of hydrogen bond donors; TPSA = topological polar surface area; PKA_SCORE = Acidity strength. Gupta, M.; Lee, H. J.; Barden, C. J.; Weaver, D. “The Blood-Brain Barrier (BBB) Score” J. Med. Chem. 2019, 62 (21), 9824-9836.

| Calc. | MarvinSketch | | IP | RB | HLB | logS | PL | MPA | MSA | HBA | MR | hERG_AM_ | hERG_CM_ |
| --- | --- | --- | --- | --- | --- | --- | --- | --- | --- | --- | --- | --- | --- |
|  |  | T0 | 8.99 | 7 | 5.43 | -5.74 | 43.63 | 51.47 | 575.38 | 7 | 112.37 | 5.27 | safe |
| ClogP | 4.28 | 0.36 |  |  |  |  |  |  |  |  |  |  |  |
| ClogD | 4.28 | 0.00 |  |  |  |  |  |  |  |  |  |  |  |
| MW | 408.88 | 0.65 |  |  |  |  |  |  |  |  |  |  |  |
| PSA | 68.29 | 1.00 |  |  |  |  |  |  |  |  |  |  |  |
| HBD | 1.00 | 0.75 |  |  |  |  |  |  |  |  |  |  |  |
| pKa | 5.02 | 1.00 |  |  |  |  |  |  |  |  |  |  |  |
| MPO | 3.76 | |  |  |  |  |  |  |  |  |  |  |  |
| BBB | 4.14 | |  |  |  |  |  |  |  |  |  |  |  |
| LogBB | -0.22 | |  |  |  |  |  |  |  |  |  |  |  |

IP = Isoelectronic Point; RB = Number of rotatable bonds; HLB = hydrophilic-lipophilic balance; logS = water solubility; PL = polarizability(Å^3^); Minimal Projection Area (Å^2^); MSA = molecular surface area; HBA = hydrogen bond acceptors; MR = Molar refractivity; ClogD = distribution at pKa; PSA = polar surface area; HBD = hydrogen bond donors; pKa = estimated acid strength; MPO = score for CNS penetration; LogBB = blood-brain distribution (0.152ClogP – 0.0148PSA + 0.138); hERG = estimated pIC50 (pAct) value for hERG (the human ether-a-go-go (hERG) ion channel); hERG_AM_ = hERG activity model; hERG_CM_ = hERG classification model

| BBB Score Calculator | | |
| --- | --- | --- |
| Property | Value | T_0_ |
| ARO_R_SCORE | 3 | 0.69 |
| HA_SCORE | 29 | 0.79 |
| HWHBN_SCORE | 0.25 | 0.96 |
| TPSA_SCORE | 68.29 | 0.52 |
| PKA_SCORE | 5.02 | 0.34 |
| BBB_SCORE | | 4.14 |

ARO_R_SCORE = Number of aromatic rings; HA_SCORE = Number of heavy atoms. MWHNM_SCORE = [-0.5MW(HBA+HBD)] where MW = molecular weight, HBA = number of hydrogen bond acceptor sites, and HBD = number of hydrogen bond donors; TPSA = topological polar surface area; PKA_SCORE = Acidity strength. Gupta, M.; Lee, H. J.; Barden, C. J.; Weaver, D. “The Blood-Brain Barrier (BBB) Score” J. Med. Chem. 2019, 62 (21), 9824-9836.

| Calc. | MarvinSketch | | IP | RB | HLB | logS | PL | MPA | MSA | HBA | MR | hERG_AM_ | hERG_CM_ |
| --- | --- | --- | --- | --- | --- | --- | --- | --- | --- | --- | --- | --- | --- |
|  |  | T0 | NA | 7 | 5.56 | -5.91 | 45.47 | 57.15 | 608.71 | 7 | 116.74 | 5.35 | safe |
| ClogP | 4.59 | 0.21 |  |  |  |  |  |  |  |  |  |  |  |
| ClogD | 4.59 | 0.00 |  |  |  |  |  |  |  |  |  |  |  |
| MW | 422.91 | 0.55 |  |  |  |  |  |  |  |  |  |  |  |
| PSA | 59.50 | 1.00 |  |  |  |  |  |  |  |  |  |  |  |
| HBD | 0.00 | 1.00 |  |  |  |  |  |  |  |  |  |  |  |
| pKa | 4.21 | 1.00 |  |  |  |  |  |  |  |  |  |  |  |
| MPO | 3.76 | |  |  |  |  |  |  |  |  |  |  |  |
| BBB | 4.60 | |  |  |  |  |  |  |  |  |  |  |  |
| LogBB | -0.04 | |  |  |  |  |  |  |  |  |  |  |  |

IP = Isoelectronic Point; RB = Number of rotatable bonds; HLB = hydrophilic-lipophilic balance; logS = water solubility; PL = polarizability(Å^3^); Minimal Projection Area (Å^2^); MSA = molecular surface area; HBA = hydrogen bond acceptors; MR = Molar refractivity; ClogD = distribution at pKa; PSA = polar surface area; HBD = hydrogen bond donors; pKa = estimated acid strength; MPO = score for CNS penetration; LogBB = blood-brain distribution (0.152ClogP – 0.0148PSA + 0.138); hERG = estimated pIC50 (pAct) value for hERG (the human ether-a-go-go (hERG) ion channel); hERG_AM_ = hERG activity model; hERG_CM_ = hERG classification model

| BBB Score Calculator | | |
| --- | --- | --- |
| Property | Value | T_0_ |
| ARO_R_SCORE | 3 | 0.69 |
| HA_SCORE | 30 | 0.75 |
| HWHBN_SCORE | 0.19 | 1.00 |
| TPSA_SCORE | 59.50 | 0.58 |
| PKA_SCORE | 8.81 | 1.00 |
| BBB_SCORE | | 4.60 |

ARO_R_SCORE = Number of aromatic rings; HA_SCORE = Number of heavy atoms. MWHNM_SCORE = [-0.5MW(HBA+HBD)] where MW = molecular weight, HBA = number of hydrogen bond acceptor sites, and HBD = number of hydrogen bond donors; TPSA = topological polar surface area; PKA_SCORE = Acidity strength. Gupta, M.; Lee, H. J.; Barden, C. J.; Weaver, D. “The Blood-Brain Barrier (BBB) Score” J. Med. Chem. 2019, 62 (21), 9824-9836.

| Calc. | MarvinSketch | | IP | RB | HLB | logS | PL | MPA | MSA | HBA | MR | hERG_AM_ | hERG_CM_ |
| --- | --- | --- | --- | --- | --- | --- | --- | --- | --- | --- | --- | --- | --- |
|  |  | T0 | NA | 7 | 5.58 | -5.77 | 45.47 | 57.81 | 608.06 | 7 | 117.26 | 5.41 | safe |
| ClogP | 4.51 | 0.25 |  |  |  |  |  |  |  |  |  |  |  |
| ClogD | 4.50 | 0.00 |  |  |  |  |  |  |  |  |  |  |  |
| MW | 422.91 | 0.55 |  |  |  |  |  |  |  |  |  |  |  |
| PSA | 59.50 | 1.00 |  |  |  |  |  |  |  |  |  |  |  |
| HBD | 0.00 | 1.00 |  |  |  |  |  |  |  |  |  |  |  |
| pKa | 4.83 | 1.00 |  |  |  |  |  |  |  |  |  |  |  |
| MPO | 3.80 | |  |  |  |  |  |  |  |  |  |  |  |
| BBB | 4.60 | |  |  |  |  |  |  |  |  |  |  |  |
| LogBB | -0.06 | |  |  |  |  |  |  |  |  |  |  |  |

IP = Isoelectronic Point; RB = Number of rotatable bonds; HLB = hydrophilic-lipophilic balance; logS = water solubility; PL = polarizability(Å^3^); Minimal Projection Area (Å^2^); MSA = molecular surface area; HBA = hydrogen bond acceptors; MR = Molar refractivity; ClogD = distribution at pKa; PSA = polar surface area; HBD = hydrogen bond donors; pKa = estimated acid strength; MPO = score for CNS penetration; LogBB = blood-brain distribution (0.152ClogP – 0.0148PSA + 0.138); hERG = estimated pIC50 (pAct) value for hERG (the human ether-a-go-go (hERG) ion channel); hERG_AM_ = hERG activity model; hERG_CM_ = hERG classification model

| BBB Score Calculator | | |
| --- | --- | --- |
| Property | Value | T_0_ |
| ARO_R_SCORE | 3 | 0.69 |
| HA_SCORE | 30 | 0.75 |
| HWHBN_SCORE | 0.19 | 1.00 |
| TPSA_SCORE | 59.50 | 0.58 |
| PKA_SCORE | 8.81 | 1.00 |
| BBB_SCORE | | 4.60 |

ARO_R_SCORE = Number of aromatic rings; HA_SCORE = Number of heavy atoms. MWHNM_SCORE = [-0.5MW(HBA+HBD)] where MW = molecular weight, HBA = number of hydrogen bond acceptor sites, and HBD = number of hydrogen bond donors; TPSA = topological polar surface area; PKA_SCORE = Acidity strength. Gupta, M.; Lee, H. J.; Barden, C. J.; Weaver, D. “The Blood-Brain Barrier (BBB) Score” J. Med. Chem. 2019, 62 (21), 9824-9836.

| Calc. | MarvinSketch | | IP | RB | HLB | logS | PL | MPA | MSA | HBA | MR | hERG_AM_ | hERG_CM_ |
| --- | --- | --- | --- | --- | --- | --- | --- | --- | --- | --- | --- | --- | --- |
|  |  | T0 | NA | 7 | 5.58 | -5.77 | 45.47 | 56.93 | 608.63 | 7 | 117.26 | 5.42 | safe |
| ClogP | 4.51 | 0.25 |  |  |  |  |  |  |  |  |  |  |  |
| ClogD | 4.50 | 0.00 |  |  |  |  |  |  |  |  |  |  |  |
| MW | 422.91 | 0.55 |  |  |  |  |  |  |  |  |  |  |  |
| PSA | 59.50 | 1.00 |  |  |  |  |  |  |  |  |  |  |  |
| HBD | 0.00 | 1.00 |  |  |  |  |  |  |  |  |  |  |  |
| pKa | 5.03 | 1.00 |  |  |  |  |  |  |  |  |  |  |  |
| MPO | 3.80 | |  |  |  |  |  |  |  |  |  |  |  |
| BBB | 4.27 | |  |  |  |  |  |  |  |  |  |  |  |
| LogBB | -0.06 | |  |  |  |  |  |  |  |  |  |  |  |

IP = Isoelectronic Point; RB = Number of rotatable bonds; HLB = hydrophilic-lipophilic balance; logS = water solubility; PL = polarizability(Å^3^); Minimal Projection Area (Å^2^); MSA = molecular surface area; HBA = hydrogen bond acceptors; MR = Molar refractivity; ClogD = distribution at pKa; PSA = polar surface area; HBD = hydrogen bond donors; pKa = estimated acid strength; MPO = score for CNS penetration; LogBB = blood-brain distribution (0.152ClogP – 0.0148PSA + 0.138); hERG = estimated pIC50 (pAct) value for hERG (the human ether-a-go-go (hERG) ion channel); hERG_AM_ = hERG activity model; hERG_CM_ = hERG classification model

| BBB Score Calculator | | |
| --- | --- | --- |
| Property | Value | T_0_ |
| ARO_R_SCORE | 3 | 0.69 |
| HA_SCORE | 30 | 0.75 |
| HWHBN_SCORE | 0.19 | 1.00 |
| TPSA_SCORE | 59.50 | 0.58 |
| PKA_SCORE | 5.03 | 0.34 |
| BBB_SCORE | | 4.27 |

ARO_R_SCORE = Number of aromatic rings; HA_SCORE = Number of heavy atoms. MWHNM_SCORE = [-0.5MW(HBA+HBD)] where MW = molecular weight, HBA = number of hydrogen bond acceptor sites, and HBD = number of hydrogen bond donors; TPSA = topological polar surface area; PKA_SCORE = Acidity strength. Gupta, M.; Lee, H. J.; Barden, C. J.; Weaver, D. “The Blood-Brain Barrier (BBB) Score” J. Med. Chem. 2019, 62 (21), 9824-9836.

| Calc. | MarvinSketch | | IP | RB | HLB | logS | PL | MPA | MSA | HBA | MR | hERG_AM_ | hERG_CM_ |
| --- | --- | --- | --- | --- | --- | --- | --- | --- | --- | --- | --- | --- | --- |
|  |  | T0 | 9.50 | 8 | 5.01 | -5.97 | 45.47 | 48.71 | 606.38 | 7 | 116.55 | 5.34 | Safe |
| ClogP | 4.60 | 0.20 |  |  |  |  |  |  |  |  |  |  |  |
| ClogD | 4.60 | 0.00 |  |  |  |  |  |  |  |  |  |  |  |
| MW | 422.91 | 0.55 |  |  |  |  |  |  |  |  |  |  |  |
| PSA | 68.29 | 1.00 |  |  |  |  |  |  |  |  |  |  |  |
| HBD | 1.00 | 0.75 |  |  |  |  |  |  |  |  |  |  |  |
| pKa | 4.55 | 1.00 |  |  |  |  |  |  |  |  |  |  |  |
| MPO | 3.50 | |  |  |  |  |  |  |  |  |  |  |  |
| BBB | 4.44 | |  |  |  |  |  |  |  |  |  |  |  |
| LogBB | -0.17 | |  |  |  |  |  |  |  |  |  |  |  |

IP = Isoelectronic Point; RB = Number of rotatable bonds; HLB = hydrophilic-lipophilic balance; logS = water solubility; PL = polarizability(Å^3^); Minimal Projection Area (Å^2^); MSA = molecular surface area; HBA = hydrogen bond acceptors; MR = Molar refractivity; ClogD = distribution at pKa; PSA = polar surface area; HBD = hydrogen bond donors; pKa = estimated acid strength; MPO = score for CNS penetration; LogBB = blood-brain distribution (0.152ClogP – 0.0148PSA + 0.138); hERG = estimated pIC50 (pAct) value for hERG (the human ether-a-go-go (hERG) ion channel); hERG_AM_ = hERG activity model; hERG_CM_ = hERG classification model

| BBB Score Calculator | | |
| --- | --- | --- |
| Property | Value | T_0_ |
| ARO_R_SCORE | 3 | 0.69 |
| HA_SCORE | 30 | 0.75 |
| HWHBN_SCORE | 0.24 | 0.97 |
| TPSA_SCORE | 68.29 | 0.52 |
| PKA_SCORE | 8.81 | 1.00 |
| BBB_SCORE | | 4.44 |

ARO_R_SCORE = Number of aromatic rings; HA_SCORE = Number of heavy atoms. MWHNM_SCORE = [-0.5MW(HBA+HBD)] where MW = molecular weight, HBA = number of hydrogen bond acceptor sites, and HBD = number of hydrogen bond donors; TPSA = topological polar surface area; PKA_SCORE = Acidity strength. Gupta, M.; Lee, H. J.; Barden, C. J.; Weaver, D. “The Blood-Brain Barrier (BBB) Score” J. Med. Chem. 2019, 62 (21), 9824-9836.

| Calc. | MarvinSketch | | IP | RB | HLB | logS | PL | MPA | MSA | HBA | MR | hERG_AM_ | hERG_CM_ |
| --- | --- | --- | --- | --- | --- | --- | --- | --- | --- | --- | --- | --- | --- |
|  |  | T0 | 9.68 | 8 | 5.03 | -5.74 | 45.47 | 46.89 | 605.78 | 7 | 117.12 | 5.39 | safe |
| ClogP | 4.57 | 0.21 |  |  |  |  |  |  |  |  |  |  |  |
| ClogD | 4.57 | 0.00 |  |  |  |  |  |  |  |  |  |  |  |
| MW | 422.91 | 0.55 |  |  |  |  |  |  |  |  |  |  |  |
| PSA | 68.29 | 1.00 |  |  |  |  |  |  |  |  |  |  |  |
| HBD | 1.00 | 0.75 |  |  |  |  |  |  |  |  |  |  |  |
| pKa | 4.93 | 1.00 |  |  |  |  |  |  |  |  |  |  |  |
| MPO | 3.52 | |  |  |  |  |  |  |  |  |  |  |  |
| BBB | 4.44 | |  |  |  |  |  |  |  |  |  |  |  |
| LogBB | -0.18 | |  |  |  |  |  |  |  |  |  |  |  |

IP = Isoelectronic Point; RB = Number of rotatable bonds; HLB = hydrophilic-lipophilic balance; logS = water solubility; PL = polarizability(Å^3^); Minimal Projection Area (Å^2^); MSA = molecular surface area; HBA = hydrogen bond acceptors; MR = Molar refractivity; ClogD = distribution at pKa; PSA = polar surface area; HBD = hydrogen bond donors; pKa = estimated acid strength; MPO = score for CNS penetration; LogBB = blood-brain distribution (0.152ClogP – 0.0148PSA + 0.138); hERG = estimated pIC50 (pAct) value for hERG (the human ether-a-go-go (hERG) ion channel); hERG_AM_ = hERG activity model; hERG_CM_ = hERG classification model

| BBB Score Calculator | | |
| --- | --- | --- |
| Property | Value | T_0_ |
| ARO_R_SCORE | 3 | 0.69 |
| HA_SCORE | 30 | 0.75 |
| HWHBN_SCORE | 0.24 | 0.97 |
| TPSA_SCORE | 68.29 | 0.52 |
| PKA_SCORE | 8.81 | 1.00 |
| BBB_SCORE | | 4.44 |

ARO_R_SCORE = Number of aromatic rings; HA_SCORE = Number of heavy atoms. MWHNM_SCORE = [-0.5MW(HBA+HBD)] where MW = molecular weight, HBA = number of hydrogen bond acceptor sites, and HBD = number of hydrogen bond donors; TPSA = topological polar surface area; PKA_SCORE = Acidity strength. Gupta, M.; Lee, H. J.; Barden, C. J.; Weaver, D. “The Blood-Brain Barrier (BBB) Score” J. Med. Chem. 2019, 62 (21), 9824-9836.

| Calc. | MarvinSketch | | IP | RB | HLB | logS | PL | MPA | MSA | HBA | MR | hERG_AM_ | hERG_CM_ |
| --- | --- | --- | --- | --- | --- | --- | --- | --- | --- | --- | --- | --- | --- |
|  |  | T0 | 9.75 | 8 | 5.03 | -5.74 | 45.47 | 49.21 | 605.22 | 7 | 117.12 | 5.45 | TOXIC |
| ClogP | 4.57 | 0.21 |  |  |  |  |  |  |  |  |  |  |  |
| ClogD | 4.57 | 0.00 |  |  |  |  |  |  |  |  |  |  |  |
| MW | 422.91 | 0.55 |  |  |  |  |  |  |  |  |  |  |  |
| PSA | 68.29 | 1.00 |  |  |  |  |  |  |  |  |  |  |  |
| HBD | 1.00 | 0.75 |  |  |  |  |  |  |  |  |  |  |  |
| pKa | 5.05 | 1.00 |  |  |  |  |  |  |  |  |  |  |  |
| MPO | 3.52 | |  |  |  |  |  |  |  |  |  |  |  |
| BBB | 4.11 | |  |  |  |  |  |  |  |  |  |  |  |
| LogBB | -0.18 | |  |  |  |  |  |  |  |  |  |  |  |

IP = Isoelectronic Point; RB = Number of rotatable bonds; HLB = hydrophilic-lipophilic balance; logS = water solubility; PL = polarizability(Å^3^); Minimal Projection Area (Å^2^); MSA = molecular surface area; HBA = hydrogen bond acceptors; MR = Molar refractivity; ClogD = distribution at pKa; PSA = polar surface area; HBD = hydrogen bond donors; pKa = estimated acid strength; MPO = score for CNS penetration; LogBB = blood-brain distribution (0.152ClogP – 0.0148PSA + 0.138); hERG = estimated pIC50 (pAct) value for hERG (the human ether-a-go-go (hERG) ion channel); hERG_AM_ = hERG activity model; hERG_CM_ = hERG classification model

| BBB Score Calculator | | |
| --- | --- | --- |
| Property | Value | T_0_ |
| ARO_R_SCORE | 3 | 0.69 |
| HA_SCORE | 30 | 0.75 |
| HWHBN_SCORE | 0.24 | 0.97 |
| TPSA_SCORE | 68.29 | 0.52 |
| PKA_SCORE | 5.05 | 0.35 |
| BBB_SCORE | | 4.11 |

ARO_R_SCORE = Number of aromatic rings; HA_SCORE = Number of heavy atoms. MWHNM_SCORE = [-0.5MW(HBA+HBD)] where MW = molecular weight, HBA = number of hydrogen bond acceptor sites, and HBD = number of hydrogen bond donors; TPSA = topological polar surface area; PKA_SCORE = Acidity strength. Gupta, M.; Lee, H. J.; Barden, C. J.; Weaver, D. “The Blood-Brain Barrier (BBB) Score” J. Med. Chem. 2019, 62 (21), 9824-9836.

| Calc. | MarvinSketch | | IP | RB | HLB | logS | PL | MPA | MSA | HBA | MR | hERG_AM_ | hERG_CM_ |
| --- | --- | --- | --- | --- | --- | --- | --- | --- | --- | --- | --- | --- | --- |
|  |  | T0 | NA | 9 | 4.76 | -6.41 | 54.16 | 77.69 | 707.76 | 8 | 138.68 | 5.24 | safe |
| ClogP | 5.18 | 0.00 |  |  |  |  |  |  |  |  |  |  |  |
| ClogD | 5.17 | 0.00 |  |  |  |  |  |  |  |  |  |  |  |
| MW | 500.00 | 0.00 |  |  |  |  |  |  |  |  |  |  |  |
| PSA | 72.39 | 1.00 |  |  |  |  |  |  |  |  |  |  |  |
| HBD | 0.00 | 1.00 |  |  |  |  |  |  |  |  |  |  |  |
| pKa | 4.49 | 1.00 |  |  |  |  |  |  |  |  |  |  |  |
| MPO | 3.00 | |  |  |  |  |  |  |  |  |  |  |  |
| BBB | 3.66 | |  |  |  |  |  |  |  |  |  |  |  |
| LogBB | -0.15 | |  |  |  |  |  |  |  |  |  |  |  |

IP = Isoelectronic Point; RB = Number of rotatable bonds; HLB = hydrophilic-lipophilic balance; logS = water solubility; PL = polarizability(Å^3^); Minimal Projection Area (Å^2^); MSA = molecular surface area; HBA = hydrogen bond acceptors; MR = Molar refractivity; ClogD = distribution at pKa; PSA = polar surface area; HBD = hydrogen bond donors; pKa = estimated acid strength; MPO = score for CNS penetration; LogBB = blood-brain distribution (0.152ClogP – 0.0148PSA + 0.138); hERG = estimated pIC50 (pAct) value for hERG (the human ether-a-go-go (hERG) ion channel); hERG_AM_ = hERG activity model; hERG_CM_ = hERG classification model

| BBB Score Calculator | | |
| --- | --- | --- |
| Property | Value | T_0_ |
| ARO_R_SCORE | 4 | 0.20 |
| HA_SCORE | 36 | 0.48 |
| HWHBN_SCORE | 0.22 | 0.99 |
| TPSA_SCORE | 72.39 | 0.49 |
| PKA_SCORE | 8.81 | 1.00 |
| BBB_SCORE | | 3.66 |

ARO_R_SCORE = Number of aromatic rings; HA_SCORE = Number of heavy atoms. MWHNM_SCORE = [-0.5MW(HBA+HBD)] where MW = molecular weight, HBA = number of hydrogen bond acceptor sites, and HBD = number of hydrogen bond donors; TPSA = topological polar surface area; PKA_SCORE = Acidity strength. Gupta, M.; Lee, H. J.; Barden, C. J.; Weaver, D. “The Blood-Brain Barrier (BBB) Score” J. Med. Chem. 2019, 62 (21), 9824-9836.

| Calc. | MarvinSketch | | IP | RB | HLB | logS | PL | MPA | MSA | HBA | MR | hERG_AM_ | hERG_CM_ |
| --- | --- | --- | --- | --- | --- | --- | --- | --- | --- | --- | --- | --- | --- |
|  |  | T0 | 6.65 | 6 | 4.59 | -7.05 | 49.90 | 50.89 | 615.89 | 9 | 127.37 | 5.27 | safe |
| ClogP | 5.65 | 0.00 |  |  |  |  |  |  |  |  |  |  |  |
| ClogD | 5.65 | 0.00 |  |  |  |  |  |  |  |  |  |  |  |
| MW | 460.91 | 0.28 |  |  |  |  |  |  |  |  |  |  |  |
| PSA | 88.52 | 1.00 |  |  |  |  |  |  |  |  |  |  |  |
| HBD | 2.00 | 0.50 |  |  |  |  |  |  |  |  |  |  |  |
| pKa | 4.53 | 1.00 |  |  |  |  |  |  |  |  |  |  |  |
| MPO | 2.78 | |  |  |  |  |  |  |  |  |  |  |  |
| BBB | 3.21 | |  |  |  |  |  |  |  |  |  |  |  |
| LogBB | -0.31 | |  |  |  |  |  |  |  |  |  |  |  |

IP = Isoelectronic Point; RB = Number of rotatable bonds; HLB = hydrophilic-lipophilic balance; logS = water solubility; PL = polarizability(Å^3^); Minimal Projection Area (Å^2^); MSA = molecular surface area; HBA = hydrogen bond acceptors; MR = Molar refractivity; ClogD = distribution at pKa; PSA = polar surface area; HBD = hydrogen bond donors; pKa = estimated acid strength; MPO = score for CNS penetration; LogBB = blood-brain distribution (0.152ClogP – 0.0148PSA + 0.138); hERG = estimated pIC50 (pAct) value for hERG (the human ether-a-go-go (hERG) ion channel); hERG_AM_ = hERG activity model; hERG_CM_ = hERG classification model

| BBB Score Calculator | | |
| --- | --- | --- |
| Property | Value | T_0_ |
| ARO_R_SCORE | 4 | 0.20 |
| HA_SCORE | 33 | 0.61 |
| HWHBN_SCORE | 0.33 | 0.76 |
| TPSA_SCORE | 88.52 | 0.38 |
| PKA_SCORE | 8.81 | 1.00 |
| BBB_SCORE | | 3.21 |

ARO_R_SCORE = Number of aromatic rings; HA_SCORE = Number of heavy atoms. MWHNM_SCORE = [-0.5MW(HBA+HBD)] where MW = molecular weight, HBA = number of hydrogen bond acceptor sites, and HBD = number of hydrogen bond donors; TPSA = topological polar surface area; PKA_SCORE = Acidity strength. Gupta, M.; Lee, H. J.; Barden, C. J.; Weaver, D. “The Blood-Brain Barrier (BBB) Score” J. Med. Chem. 2019, 62 (21), 9824-9836.

| Calc. | MarvinSketch | | IP | RB | HLB | logS | PL | MPA | MSA | HBA | MR | hERG_AM_ | hERG_CM_ |
| --- | --- | --- | --- | --- | --- | --- | --- | --- | --- | --- | --- | --- | --- |
|  |  | T0 | 9.01 | 6 | 3.40 | -9.08 | 56.85 | 56.88 | 666.84 | 7 | 141.47 | 5.62 | Safe |
| ClogP | 7.33 | 0.00 |  |  |  |  |  |  |  |  |  |  |  |
| ClogD | 7.32 | 0.00 |  |  |  |  |  |  |  |  |  |  |  |
| MW | 494.98 | 0.04 |  |  |  |  |  |  |  |  |  |  |  |
| PSA | 68.29 | 1.00 |  |  |  |  |  |  |  |  |  |  |  |
| HBD | 1.00 | 0.75 |  |  |  |  |  |  |  |  |  |  |  |
| pKa | 5.55 | 1.00 |  |  |  |  |  |  |  |  |  |  |  |
| MPO | 2.79 | |  |  |  |  |  |  |  |  |  |  |  |
| BBB | 3.25 | |  |  |  |  |  |  |  |  |  |  |  |
| LogBB | 0.24 | |  |  |  |  |  |  |  |  |  |  |  |

IP = Isoelectronic Point; RB = Number of rotatable bonds; HLB = hydrophilic-lipophilic balance; logS = water solubility; PL = polarizability(Å^3^); Minimal Projection Area (Å^2^); MSA = molecular surface area; HBA = hydrogen bond acceptors; MR = Molar refractivity; ClogD = distribution at pKa; PSA = polar surface area; HBD = hydrogen bond donors; pKa = estimated acid strength; MPO = score for CNS penetration; LogBB = blood-brain distribution (0.152ClogP – 0.0148PSA + 0.138); hERG = estimated pIC50 (pAct) value for hERG (the human ether-a-go-go (hERG) ion channel); hERG_AM_ = hERG activity model; hERG_CM_ = hERG classification model

| BBB Score Calculator | | |
| --- | --- | --- |
| Property | Value | T_0_ |
| ARO_R_SCORE | 5 | 0.00 |
| HA_SCORE | 36 | 0.48 |
| HWHBN_SCORE | 0.22 | 0.99 |
| TPSA_SCORE | 68.29 | 0.52 |
| PKA_SCORE | 5.55 | 0.48 |
| BBB_SCORE | | 3.25 |

ARO_R_SCORE = Number of aromatic rings; HA_SCORE = Number of heavy atoms. MWHNM_SCORE = [-0.5MW(HBA+HBD)] where MW = molecular weight, HBA = number of hydrogen bond acceptor sites, and HBD = number of hydrogen bond donors; TPSA = topological polar surface area; PKA_SCORE = Acidity strength. Gupta, M.; Lee, H. J.; Barden, C. J.; Weaver, D. “The Blood-Brain Barrier (BBB) Score” J. Med. Chem. 2019, 62 (21), 9824-9836.

| Calc. | MarvinSketch | | IP | RB | HLB | logS | PL | MPA | MSA | HBA | MR | hERG_AM_ | hERG_CM_ |
| --- | --- | --- | --- | --- | --- | --- | --- | --- | --- | --- | --- | --- | --- |
|  |  | T0 | 8.09 | 7 | 4.99 | -6.28 | 45.47 | 56.50 | 603.44 | 7 | 116.34 | 5.24 | Safe |
| ClogP | 4.93 | 0.03 |  |  |  |  |  |  |  |  |  |  |  |
| ClogD | 4.93 | 0.00 |  |  |  |  |  |  |  |  |  |  |  |
| MW | 422.91 | 0.55 |  |  |  |  |  |  |  |  |  |  |  |
| PSA | 68.29 | 1.00 |  |  |  |  |  |  |  |  |  |  |  |
| HBD | 1.00 | 0.75 |  |  |  |  |  |  |  |  |  |  |  |
| pKa | 4.09 | 1.00 |  |  |  |  |  |  |  |  |  |  |  |
| MPO | 3.33 | |  |  |  |  |  |  |  |  |  |  |  |
| BBB | 4.44 | |  |  |  |  |  |  |  |  |  |  |  |
| LogBB | -0.12 | |  |  |  |  |  |  |  |  |  |  |  |

IP = Isoelectronic Point; RB = Number of rotatable bonds; HLB = hydrophilic-lipophilic balance; logS = water solubility; PL = polarizability(Å^3^); Minimal Projection Area (Å^2^); MSA = molecular surface area; HBA = hydrogen bond acceptors; MR = Molar refractivity; ClogD = distribution at pKa; PSA = polar surface area; HBD = hydrogen bond donors; pKa = estimated acid strength; MPO = score for CNS penetration; LogBB = blood-brain distribution (0.152ClogP – 0.0148PSA + 0.138); hERG = estimated pIC50 (pAct) value for hERG (the human ether-a-go-go (hERG) ion channel); hERG_AM_ = hERG activity model; hERG_CM_ = hERG classification model

| BBB Score Calculator | | |
| --- | --- | --- |
| Property | Value | T_0_ |
| ARO_R_SCORE | 3 | 0.69 |
| HA_SCORE | 30 | 0.75 |
| HWHBN_SCORE | 0.24 | 0.97 |
| TPSA_SCORE | 68.29 | 0.52 |
| PKA_SCORE | 8.81 | 1.00 |
| BBB_SCORE | | 4.44 |

ARO_R_SCORE = Number of aromatic rings; HA_SCORE = Number of heavy atoms. MWHNM_SCORE = [-0.5MW(HBA+HBD)] where MW = molecular weight, HBA = number of hydrogen bond acceptor sites, and HBD = number of hydrogen bond donors; TPSA = topological polar surface area; PKA_SCORE = Acidity strength. Gupta, M.; Lee, H. J.; Barden, C. J.; Weaver, D. “The Blood-Brain Barrier (BBB) Score” J. Med. Chem. 2019, 62 (21), 9824-9836.

| Calc. | MarvinSketch | | IP | RB | HLB | logS | PL | MPA | MSA | HBA | MR | hERG_AM_ | hERG_CM_ |
| --- | --- | --- | --- | --- | --- | --- | --- | --- | --- | --- | --- | --- | --- |
|  |  | T0 | 8.09 | 7 | 4.99 | -6.28 | 45.47 | 56.50 | 603.44 | 7 | 116.34 | 5.26 | safe |
| ClogP | 4.93 | 0.03 |  |  |  |  |  |  |  |  |  |  |  |
| ClogD | 4.93 | 0.00 |  |  |  |  |  |  |  |  |  |  |  |
| MW | 422.91 | 0.55 |  |  |  |  |  |  |  |  |  |  |  |
| PSA | 68.29 | 1.00 |  |  |  |  |  |  |  |  |  |  |  |
| HBD | 1.00 | 0.75 |  |  |  |  |  |  |  |  |  |  |  |
| pKa | 4.09 | 1.00 |  |  |  |  |  |  |  |  |  |  |  |
| MPO | 3.33 | |  |  |  |  |  |  |  |  |  |  |  |
| BBB | 4.44 | |  |  |  |  |  |  |  |  |  |  |  |
| LogBB | -0.12 | |  |  |  |  |  |  |  |  |  |  |  |

IP = Isoelectronic Point; RB = Number of rotatable bonds; HLB = hydrophilic-lipophilic balance; logS = water solubility; PL = polarizability(Å^3^); Minimal Projection Area (Å^2^); MSA = molecular surface area; HBA = hydrogen bond acceptors; MR = Molar refractivity; ClogD = distribution at pKa; PSA = polar surface area; HBD = hydrogen bond donors; pKa = estimated acid strength; MPO = score for CNS penetration; LogBB = blood-brain distribution (0.152ClogP – 0.0148PSA + 0.138); hERG = estimated pIC50 (pAct) value for hERG (the human ether-a-go-go (hERG) ion channel); hERG_AM_ = hERG activity model; hERG_CM_ = hERG classification model

| BBB Score Calculator | | |
| --- | --- | --- |
| Property | Value | T_0_ |
| ARO_R_SCORE | 3 | 0.69 |
| HA_SCORE | 30 | 0.75 |
| HWHBN_SCORE | 0.24 | 0.97 |
| TPSA_SCORE | 68.29 | 0.52 |
| PKA_SCORE | 8.81 | 1.00 |
| BBB_SCORE | | 4.44 |

ARO_R_SCORE = Number of aromatic rings; HA_SCORE = Number of heavy atoms. MWHNM_SCORE = [-0.5MW(HBA+HBD)] where MW = molecular weight, HBA = number of hydrogen bond acceptor sites, and HBD = number of hydrogen bond donors; TPSA = topological polar surface area; PKA_SCORE = Acidity strength. Gupta, M.; Lee, H. J.; Barden, C. J.; Weaver, D. “The Blood-Brain Barrier (BBB) Score” J. Med. Chem. 2019, 62 (21), 9824-9836.

| Calc. | MarvinSketch | | IP | RB | HLB | logS | PL | MPA | MSA | HBA | MR | hERG_AM_ | hERG_CM_ |
| --- | --- | --- | --- | --- | --- | --- | --- | --- | --- | --- | --- | --- | --- |
|  |  | T0 | 8.67 | 7 | 5.01 | -6.05 | 45.67 | 57.16 | 604.58 | 7 | 116.79 | 5.39 | safe |
| ClogP | 4.70 | 0.15 |  |  |  |  |  |  |  |  |  |  |  |
| ClogD | 4.70 | 0.00 |  |  |  |  |  |  |  |  |  |  |  |
| MW | 422.91 | 0.55 |  |  |  |  |  |  |  |  |  |  |  |
| PSA | 68.29 | 1.00 |  |  |  |  |  |  |  |  |  |  |  |
| HBD | 1.00 | 0.75 |  |  |  |  |  |  |  |  |  |  |  |
| pKa | 4.80 | 1.00 |  |  |  |  |  |  |  |  |  |  |  |
| MPO | 3.45 | |  |  |  |  |  |  |  |  |  |  |  |
| BBB | 4.44 | |  |  |  |  |  |  |  |  |  |  |  |
| LogBB | -0.16 | |  |  |  |  |  |  |  |  |  |  |  |

IP = Isoelectronic Point; RB = Number of rotatable bonds; HLB = hydrophilic-lipophilic balance; logS = water solubility; PL = polarizability(Å^3^); Minimal Projection Area (Å^2^); MSA = molecular surface area; HBA = hydrogen bond acceptors; MR = Molar refractivity; ClogD = distribution at pKa; PSA = polar surface area; HBD = hydrogen bond donors; pKa = estimated acid strength; MPO = score for CNS penetration; LogBB = blood-brain distribution (0.152ClogP – 0.0148PSA + 0.138); hERG = estimated pIC50 (pAct) value for hERG (the human ether-a-go-go (hERG) ion channel); hERG_AM_ = hERG activity model; hERG_CM_ = hERG classification model

| BBB Score Calculator | | |
| --- | --- | --- |
| Property | Value | T_0_ |
| ARO_R_SCORE | 3 | 0.69 |
| HA_SCORE | 30 | 0.75 |
| HWHBN_SCORE | 0.24 | 0.97 |
| TPSA_SCORE | 68.29 | 0.52 |
| PKA_SCORE | 8.81 | 1.00 |
| BBB_SCORE | | 4.44 |

ARO_R_SCORE = Number of aromatic rings; HA_SCORE = Number of heavy atoms. MWHNM_SCORE = [-0.5MW(HBA+HBD)] where MW = molecular weight, HBA = number of hydrogen bond acceptor sites, and HBD = number of hydrogen bond donors; TPSA = topological polar surface area; PKA_SCORE = Acidity strength. Gupta, M.; Lee, H. J.; Barden, C. J.; Weaver, D. “The Blood-Brain Barrier (BBB) Score” J. Med. Chem. 2019, 62 (21), 9824-9836.

| Calc. | MarvinSketch | | IP | RB | HLB | logS | PL | MPA | MSA | HBA | MR | hERG_AM_ | hERG_CM_ |
| --- | --- | --- | --- | --- | --- | --- | --- | --- | --- | --- | --- | --- | --- |
|  |  | T0 | 8.67 | 7 | 5.01 | -6.05 | 45.67 | 57.16 | 604.58 | 7 | 116.79 | 5.36 | safe |
| ClogP | 4.70 | 0.15 |  |  |  |  |  |  |  |  |  |  |  |
| ClogD | 4.70 | 0.00 |  |  |  |  |  |  |  |  |  |  |  |
| MW | 422.91 | 0.55 |  |  |  |  |  |  |  |  |  |  |  |
| PSA | 68.29 | 1.00 |  |  |  |  |  |  |  |  |  |  |  |
| HBD | 1.00 | 0.75 |  |  |  |  |  |  |  |  |  |  |  |
| pKa | 4.79 | 1.00 |  |  |  |  |  |  |  |  |  |  |  |
| MPO | 3.45 | |  |  |  |  |  |  |  |  |  |  |  |
| BBB | 3.40 | |  |  |  |  |  |  |  |  |  |  |  |
| LogBB | -0.16 | |  |  |  |  |  |  |  |  |  |  |  |

IP = Isoelectronic Point; RB = Number of rotatable bonds; HLB = hydrophilic-lipophilic balance; logS = water solubility; PL = polarizability(Å^3^); Minimal Projection Area (Å^2^); MSA = molecular surface area; HBA = hydrogen bond acceptors; MR = Molar refractivity; ClogD = distribution at pKa; PSA = polar surface area; HBD = hydrogen bond donors; pKa = estimated acid strength; MPO = score for CNS penetration; LogBB = blood-brain distribution (0.152ClogP – 0.0148PSA + 0.138); hERG = estimated pIC50 (pAct) value for hERG (the human ether-a-go-go (hERG) ion channel); hERG_AM_ = hERG activity model; hERG_CM_ = hERG classification model

| BBB Score Calculator | | |
| --- | --- | --- |
| Property | Value | T_0_ |
| ARO_R_SCORE | 3 | 0.69 |
| HA_SCORE | 30 | 0.75 |
| HWHBN_SCORE | 0.24 | 0.97 |
| TPSA_SCORE | 68.29 | 0.52 |
| PKA_SCORE | 8.81 | 1.00 |
| BBB_SCORE | | 4.44 |

ARO_R_SCORE = Number of aromatic rings; HA_SCORE = Number of heavy atoms. MWHNM_SCORE = [-0.5MW(HBA+HBD)] where MW = molecular weight, HBA = number of hydrogen bond acceptor sites, and HBD = number of hydrogen bond donors; TPSA = topological polar surface area; PKA_SCORE = Acidity strength. Gupta, M.; Lee, H. J.; Barden, C. J.; Weaver, D. “The Blood-Brain Barrier (BBB) Score” J. Med. Chem. 2019, 62 (21), 9824-9836.

| Calc. | MarvinSketch | | IP | RB | HLB | logS | PL | MPA | MSA | HBA | MR | hERG_AM_ | hERG_CM_ |
| --- | --- | --- | --- | --- | --- | --- | --- | --- | --- | --- | --- | --- | --- |
|  |  | T0 | NA | 6 | 13.41 | -5.75 | 42.37 | 45.59 | 554.75 | 9 | 122.36 | 5.17 | safe |
| ClogP | 4.87 | 0.07 |  |  |  |  |  |  |  |  |  |  |  |
| ClogD | 4.63 | 0.00 |  |  |  |  |  |  |  |  |  |  |  |
| MW | 410.85 | 0.64 |  |  |  |  |  |  |  |  |  |  |  |
| PSA | 92.60 | 0.91 |  |  |  |  |  |  |  |  |  |  |  |
| HBD | 2.00 | 0.50 |  |  |  |  |  |  |  |  |  |  |  |
| pKa | 3.92 | 1.00 |  |  |  |  |  |  |  |  |  |  |  |
| MPO | 3.12 | |  |  |  |  |  |  |  |  |  |  |  |
| BBB | 3.92 | |  |  |  |  |  |  |  |  |  |  |  |
| LogBB | -0.49 | |  |  |  |  |  |  |  |  |  |  |  |

IP = Isoelectronic Point; RB = Number of rotatable bonds; HLB = hydrophilic-lipophilic balance; logS = water solubility; PL = polarizability(Å^3^); Minimal Projection Area (Å^2^); MSA = molecular surface area; HBA = hydrogen bond acceptors; MR = Molar refractivity; ClogD = distribution at pKa; PSA = polar surface area; HBD = hydrogen bond donors; pKa = estimated acid strength; MPO = score for CNS penetration; LogBB = blood-brain distribution (0.152ClogP – 0.0148PSA + 0.138); hERG = estimated pIC50 (pAct) value for hERG (the human ether-a-go-go (hERG) ion channel); hERG_AM_ = hERG activity model; hERG_CM_ = hERG classification model

| BBB Score Calculator | | |
| --- | --- | --- |
| Property | Value | T_0_ |
| ARO_R_SCORE | 3 | 0.69 |
| HA_SCORE | 29 | 0.79 |
| HWHBN_SCORE | 0.30 | 0.85 |
| TPSA_SCORE | 92.60 | 0.35 |
| PKA_SCORE | 7.52 | 0.90 |
| BBB_SCORE | | 3.92 |

ARO_R_SCORE = Number of aromatic rings; HA_SCORE = Number of heavy atoms. MWHNM_SCORE = [-0.5MW(HBA+HBD)] where MW = molecular weight, HBA = number of hydrogen bond acceptor sites, and HBD = number of hydrogen bond donors; TPSA = topological polar surface area; PKA_SCORE = Acidity strength. Gupta, M.; Lee, H. J.; Barden, C. J.; Weaver, D. “The Blood-Brain Barrier (BBB) Score” J. Med. Chem. 2019, 62 (21), 9824-9836.

| Calc. | MarvinSketch | | IP | RB | HLB | logS | PL | MPA | MSA | HBA | MR | hERG_AM_ | hERG_CM_ |
| --- | --- | --- | --- | --- | --- | --- | --- | --- | --- | --- | --- | --- | --- |
|  |  | T0 | 9.52 | 6 | 12.54 | -5.97 | 42.86 | 47.06 | 540.14 | 9 | 112.46 | 5.52 | toxic |
| ClogP | 3.72 | 0.64 |  |  |  |  |  |  |  |  |  |  |  |
| ClogD | 3.39 | 0.31 |  |  |  |  |  |  |  |  |  |  |  |
| MW | 412.87 | 0.62 |  |  |  |  |  |  |  |  |  |  |  |
| PSA | 87.66 | 1.00 |  |  |  |  |  |  |  |  |  |  |  |
| HBD | 3.00 | 0.25 |  |  |  |  |  |  |  |  |  |  |  |
| pKa | 4.84 | 1.00 |  |  |  |  |  |  |  |  |  |  |  |
| MPO | 3.82 | |  |  |  |  |  |  |  |  |  |  |  |
| BBB | 3.75 | |  |  |  |  |  |  |  |  |  |  |  |
| LogBB | -0.63 | |  |  |  |  |  |  |  |  |  |  |  |

IP = Isoelectronic Point; RB = Number of rotatable bonds; HLB = hydrophilic-lipophilic balance; logS = water solubility; PL = polarizability(Å^3^); Minimal Projection Area (Å^2^); MSA = molecular surface area; HBA = hydrogen bond acceptors; MR = Molar refractivity; ClogD = distribution at pKa; PSA = polar surface area; HBD = hydrogen bond donors; pKa = estimated acid strength; MPO = score for CNS penetration; LogBB = blood-brain distribution (0.152ClogP – 0.0148PSA + 0.138); hERG = estimated pIC50 (pAct) value for hERG (the human ether-a-go-go (hERG) ion channel); hERG_AM_ = hERG activity model; hERG_CM_ = hERG classification model

| BBB Score Calculator | | |
| --- | --- | --- |
| Property | Value | T_0_ |
| ARO_R_SCORE | 2 | 1.00 |
| HA_SCORE | 29 | 0.79 |
| HWHBN_SCORE | 0.39 | 0.50 |
| TPSA_SCORE | 87.66 | 0.39 |
| PKA_SCORE | 7.35 | 0.88 |
| BBB_SCORE | | 3.75 |

ARO_R_SCORE = Number of aromatic rings; HA_SCORE = Number of heavy atoms. MWHNM_SCORE = [-0.5MW(HBA+HBD)] where MW = molecular weight, HBA = number of hydrogen bond acceptor sites, and HBD = number of hydrogen bond donors; TPSA = topological polar surface area; PKA_SCORE = Acidity strength. Gupta, M.; Lee, H. J.; Barden, C. J.; Weaver, D. “The Blood-Brain Barrier (BBB) Score” J. Med. Chem. 2019, 62 (21), 9824-9836.

| Calc. | MarvinSketch | | IP | RB | HLB | logS | PL | MPA | MSA | HBA | MR | hERG_AM_ | hERG_CM_ |
| --- | --- | --- | --- | --- | --- | --- | --- | --- | --- | --- | --- | --- | --- |
|  |  | T0 | 6.47 | 6 | 5.97 | -7.20 | 42.15 | 54.68 | 535.28 | 9 | 110.48 | 5.33 | safe |
| ClogP | 4.07 | 0.46 |  |  |  |  |  |  |  |  |  |  |  |
| ClogD | 4.07 | 0.00 |  |  |  |  |  |  |  |  |  |  |  |
| MW | 410.85 | 0.64 |  |  |  |  |  |  |  |  |  |  |  |
| PSA | 84.83 | 1.00 |  |  |  |  |  |  |  |  |  |  |  |
| HBD | 1.00 | 0.75 |  |  |  |  |  |  |  |  |  |  |  |
| pKa | 2.62 | 1.00 |  |  |  |  |  |  |  |  |  |  |  |
| MPO | 3.85 | |  |  |  |  |  |  |  |  |  |  |  |
| BBB | 4.38 | |  |  |  |  |  |  |  |  |  |  |  |
| LogBB | -0.50 | |  |  |  |  |  |  |  |  |  |  |  |

IP = Isoelectronic Point; RB = Number of rotatable bonds; HLB = hydrophilic-lipophilic balance; logS = water solubility; PL = polarizability(Å^3^); Minimal Projection Area (Å^2^); MSA = molecular surface area; HBA = hydrogen bond acceptors; MR = Molar refractivity; ClogD = distribution at pKa; PSA = polar surface area; HBD = hydrogen bond donors; pKa = estimated acid strength; MPO = score for CNS penetration; LogBB = blood-brain distribution (0.152ClogP – 0.0148PSA + 0.138); hERG = estimated pIC50 (pAct) value for hERG (the human ether-a-go-go (hERG) ion channel); hERG_AM_ = hERG activity model; hERG_CM_ = hERG classification model

| BBB Score Calculator | | |
| --- | --- | --- |
| Property | Value | T_0_ |
| ARO_R_SCORE | 2 | 1.00 |
| HA_SCORE | 29 | 0.79 |
| HWHBN_SCORE | 0.30 | 0.85 |
| TPSA_SCORE | 84.83 | 0.41 |
| PKA_SCORE | 8.81 | 1.00 |
| BBB_SCORE | | 3.75 |

ARO_R_SCORE = Number of aromatic rings; HA_SCORE = Number of heavy atoms. MWHNM_SCORE = [-0.5MW(HBA+HBD)] where MW = molecular weight, HBA = number of hydrogen bond acceptor sites, and HBD = number of hydrogen bond donors; TPSA = topological polar surface area; PKA_SCORE = Acidity strength. Gupta, M.; Lee, H. J.; Barden, C. J.; Weaver, D. “The Blood-Brain Barrier (BBB) Score” J. Med. Chem. 2019, 62 (21), 9824-9836.

| Calc. | MarvinSketch | | IP | RB | HLB | logS | PL | MPA | MSA | HBA | MR | hERG_AM_ | hERG_CM_ |
| --- | --- | --- | --- | --- | --- | --- | --- | --- | --- | --- | --- | --- | --- |
|  |  | T0 | 6.16 | 6 | 5.62 | -5.90 | 42.40 | 45.14 | 553.00 | 9 | 111.61 | 5.22 | safe |
| ClogP | 4.87 | 0.07 |  |  |  |  |  |  |  |  |  |  |  |
| ClogD | 4.63 | 0.00 |  |  |  |  |  |  |  |  |  |  |  |
| MW | 410.85 | 0.64 |  |  |  |  |  |  |  |  |  |  |  |
| PSA | 88.52 | 1.00 |  |  |  |  |  |  |  |  |  |  |  |
| HBD | 2.00 | 0.50 |  |  |  |  |  |  |  |  |  |  |  |
| pKa | 3.92 | 1.00 |  |  |  |  |  |  |  |  |  |  |  |
| MPO | 3.20 | |  |  |  |  |  |  |  |  |  |  |  |
| BBB | 3.72 | |  |  |  |  |  |  |  |  |  |  |  |
| LogBB | -0.43 | |  |  |  |  |  |  |  |  |  |  |  |

IP = Isoelectronic Point; RB = Number of rotatable bonds; HLB = hydrophilic-lipophilic balance; logS = water solubility; PL = polarizability(Å^3^); Minimal Projection Area (Å^2^); MSA = molecular surface area; HBA = hydrogen bond acceptors; MR = Molar refractivity; ClogD = distribution at pKa; PSA = polar surface area; HBD = hydrogen bond donors; pKa = estimated acid strength; MPO = score for CNS penetration; LogBB = blood-brain distribution (0.152ClogP – 0.0148PSA + 0.138); hERG = estimated pIC50 (pAct) value for hERG (the human ether-a-go-go (hERG) ion channel); hERG_AM_ = hERG activity model; hERG_CM_ = hERG classification model

| BBB Score Calculator | | |
| --- | --- | --- |
| Property | Value | T_0_ |
| ARO_R_SCORE | 3 | 0.69 |
| HA_SCORE | 29 | 0.79 |
| HWHBN_SCORE | 0.35 | 0.69 |
| TPSA_SCORE | 84.52 | 0.38 |
| PKA_SCORE | 7.52 | 0.90 |
| BBB_SCORE | | 3.72 |

ARO_R_SCORE = Number of aromatic rings; HA_SCORE = Number of heavy atoms. MWHNM_SCORE = [-0.5MW(HBA+HBD)] where MW = molecular weight, HBA = number of hydrogen bond acceptor sites, and HBD = number of hydrogen bond donors; TPSA = topological polar surface area; PKA_SCORE = Acidity strength. Gupta, M.; Lee, H. J.; Barden, C. J.; Weaver, D. “The Blood-Brain Barrier (BBB) Score” J. Med. Chem. 2019, 62 (21), 9824-9836.

| Calc. | MarvinSketch | | IP | RB | HLB | logS | PL | MPA | MSA | HBA | MR | hERG_AM_ | hERG_CM_ |
| --- | --- | --- | --- | --- | --- | --- | --- | --- | --- | --- | --- | --- | --- |
|  |  | T0 | NA | 6 | 7.23 | -6.49 | 42.32 | 43.44 | 529.29 | 8 | 111.66 | 5.13 | safe |
| ClogP | 3.53 | 0.73 |  |  |  |  |  |  |  |  |  |  |  |
| ClogD | 3.53 | 0.24 |  |  |  |  |  |  |  |  |  |  |  |
| MW | 410.85 | 0.64 |  |  |  |  |  |  |  |  |  |  |  |
| PSA | 84.50 | 1.00 |  |  |  |  |  |  |  |  |  |  |  |
| HBD | 2.00 | 0.50 |  |  |  |  |  |  |  |  |  |  |  |
| pKa | 1.82 | 1.00 |  |  |  |  |  |  |  |  |  |  |  |
| MPO | 4.11 | |  |  |  |  |  |  |  |  |  |  |  |
| BBB | 4.08 | |  |  |  |  |  |  |  |  |  |  |  |
| LogBB | -0.57 | |  |  |  |  |  |  |  |  |  |  |  |

IP = Isoelectronic Point; RB = Number of rotatable bonds; HLB = hydrophilic-lipophilic balance; logS = water solubility; PL = polarizability(Å^3^); Minimal Projection Area (Å^2^); MSA = molecular surface area; HBA = hydrogen bond acceptors; MR = Molar refractivity; ClogD = distribution at pKa; PSA = polar surface area; HBD = hydrogen bond donors; pKa = estimated acid strength; MPO = score for CNS penetration; LogBB = blood-brain distribution (0.152ClogP – 0.0148PSA + 0.138); hERG = estimated pIC50 (pAct) value for hERG (the human ether-a-go-go (hERG) ion channel); hERG_AM_ = hERG activity model; hERG_CM_ = hERG classification model

| BBB Score Calculator | | |
| --- | --- | --- |
| Property | Value | T_0_ |
| ARO_R_SCORE | 3 | 0.69 |
| HA_SCORE | 29 | 0.79 |
| HWHBN_SCORE | 0.30 | 0.85 |
| TPSA_SCORE | 84.50 | 0.41 |
| PKA_SCORE | 8.81 | 1.00 |
| BBB_SCORE | | 4.08 |

ARO_R_SCORE = Number of aromatic rings; HA_SCORE = Number of heavy atoms. MWHNM_SCORE = [-0.5MW(HBA+HBD)] where MW = molecular weight, HBA = number of hydrogen bond acceptor sites, and HBD = number of hydrogen bond donors; TPSA = topological polar surface area; PKA_SCORE = Acidity strength. Gupta, M.; Lee, H. J.; Barden, C. J.; Weaver, D. “The Blood-Brain Barrier (BBB) Score” J. Med. Chem. 2019, 62 (21), 9824-9836.

| Calc. | MarvinSketch | | IP | RB | HLB | logS | PL | MPA | MSA | HBA | MR | hERG_AM_ | hERG_CM_ |
| --- | --- | --- | --- | --- | --- | --- | --- | --- | --- | --- | --- | --- | --- |
|  |  | T0 | 6.17 | 6 | 5.85 | -6.00 | 42.40 | 43.69 | 552.22 | 9 | 111.61 | 5.21 | Safe |
| ClogP | 4.87 | 0.07 |  |  |  |  |  |  |  |  |  |  |  |
| ClogD | 4.87 | 0.00 |  |  |  |  |  |  |  |  |  |  |  |
| MW | 410.85 | 0.64 |  |  |  |  |  |  |  |  |  |  |  |
| PSA | 88.52 | 1.00 |  |  |  |  |  |  |  |  |  |  |  |
| HBD | 2.00 | 0.50 |  |  |  |  |  |  |  |  |  |  |  |
| pKa | 1.82 | 1.00 |  |  |  |  |  |  |  |  |  |  |  |
| MPO | 3.20 | |  |  |  |  |  |  |  |  |  |  |  |
| BBB | 3.77 | |  |  |  |  |  |  |  |  |  |  |  |
| LogBB | -0.43 | |  |  |  |  |  |  |  |  |  |  |  |

IP = Isoelectronic Point; RB = Number of rotatable bonds; HLB = hydrophilic-lipophilic balance; logS = water solubility; PL = polarizability(Å^3^); Minimal Projection Area (Å^2^); MSA = molecular surface area; HBA = hydrogen bond acceptors; MR = Molar refractivity; ClogD = distribution at pKa; PSA = polar surface area; HBD = hydrogen bond donors; pKa = estimated acid strength; MPO = score for CNS penetration; LogBB = blood-brain distribution (0.152ClogP – 0.0148PSA + 0.138); hERG = estimated pIC50 (pAct) value for hERG (the human ether-a-go-go (hERG) ion channel); hERG_AM_ = hERG activity model; hERG_CM_ = hERG classification model

| BBB Score Calculator | | |
| --- | --- | --- |
| Property | Value | T_0_ |
| ARO_R_SCORE | 3 | 0.69 |
| HA_SCORE | 29 | 0.79 |
| HWHBN_SCORE | 0.35 | 0.69 |
| TPSA_SCORE | 88.52 | 0.38 |
| PKA_SCORE | 8.81 | 1.00 |
| BBB_SCORE | | 3.77 |

ARO_R_SCORE = Number of aromatic rings; HA_SCORE = Number of heavy atoms. MWHNM_SCORE = [-0.5MW(HBA+HBD)] where MW = molecular weight, HBA = number of hydrogen bond acceptor sites, and HBD = number of hydrogen bond donors; TPSA = topological polar surface area; PKA_SCORE = Acidity strength. Gupta, M.; Lee, H. J.; Barden, C. J.; Weaver, D. “The Blood-Brain Barrier (BBB) Score” J. Med. Chem. 2019, 62 (21), 9824-9836.

| Calc. | MarvinSketch | | IP | RB | HLB | logS | PL | MPA | MSA | HBA | MR | hERG_AM_ | hERG_CM_ |
| --- | --- | --- | --- | --- | --- | --- | --- | --- | --- | --- | --- | --- | --- |
|  |  | T0 | NA | 6 | 7.23 | -6.49 | 42.32 | 42.75 | 530.46 | 8 | 111.59 | 5.16 | safe |
| ClogP | 3.38 | 0.81 |  |  |  |  |  |  |  |  |  |  |  |
| ClogD | 3.38 | 0.31 |  |  |  |  |  |  |  |  |  |  |  |
| MW | 410.85 | 0.64 |  |  |  |  |  |  |  |  |  |  |  |
| PSA | 84.50 | 1.00 |  |  |  |  |  |  |  |  |  |  |  |
| HBD | 2.00 | 0.50 |  |  |  |  |  |  |  |  |  |  |  |
| pKa | 1.80 | 1.00 |  |  |  |  |  |  |  |  |  |  |  |
| MPO | 4.26 | |  |  |  |  |  |  |  |  |  |  |  |
| BBB | 4.08 | |  |  |  |  |  |  |  |  |  |  |  |
| LogBB | -0.60 | |  |  |  |  |  |  |  |  |  |  |  |

IP = Isoelectronic Point; RB = Number of rotatable bonds; HLB = hydrophilic-lipophilic balance; logS = water solubility; PL = polarizability(Å^3^); Minimal Projection Area (Å^2^); MSA = molecular surface area; HBA = hydrogen bond acceptors; MR = Molar refractivity; ClogD = distribution at pKa; PSA = polar surface area; HBD = hydrogen bond donors; pKa = estimated acid strength; MPO = score for CNS penetration; LogBB = blood-brain distribution (0.152ClogP – 0.0148PSA + 0.138); hERG = estimated pIC50 (pAct) value for hERG (the human ether-a-go-go (hERG) ion channel); hERG_AM_ = hERG activity model; hERG_CM_ = hERG classification model

| BBB Score Calculator | | |
| --- | --- | --- |
| Property | Value | T_0_ |
| ARO_R_SCORE | 3 | 0.69 |
| HA_SCORE | 29 | 0.79 |
| HWHBN_SCORE | 0.30 | 0.85 |
| TPSA_SCORE | 84.50 | 0.41 |
| PKA_SCORE | 8.81 | 1.00 |
| BBB_SCORE | | 4.08 |

ARO_R_SCORE = Number of aromatic rings; HA_SCORE = Number of heavy atoms. MWHNM_SCORE = [-0.5MW(HBA+HBD)] where MW = molecular weight, HBA = number of hydrogen bond acceptor sites, and HBD = number of hydrogen bond donors; TPSA = topological polar surface area; PKA_SCORE = Acidity strength. Gupta, M.; Lee, H. J.; Barden, C. J.; Weaver, D. “The Blood-Brain Barrier (BBB) Score” J. Med. Chem. 2019, 62 (21), 9824-9836.

| Calc. | MarvinSketch | | IP | RB | HLB | logS | PL | MPA | MSA | HBA | MR | hERG_AM_ | hERG_CM_ |
| --- | --- | --- | --- | --- | --- | --- | --- | --- | --- | --- | --- | --- | --- |
|  |  | T0 | 6.92 | 6 | 5.85 | -6.00 | 42.40 | 44.16 | 554.21 | 9 | 111.61 | 5.21 | safe |
| ClogP | 4.87 | 0.07 |  |  |  |  |  |  |  |  |  |  |  |
| ClogD | 4.87 | 0.00 |  |  |  |  |  |  |  |  |  |  |  |
| MW | 410.85 | 0.64 |  |  |  |  |  |  |  |  |  |  |  |
| PSA | 88.52 | 1.00 |  |  |  |  |  |  |  |  |  |  |  |
| HBD | 2.00 | 0.5 |  |  |  |  |  |  |  |  |  |  |  |
| pKa | 2.21 | 1.00 |  |  |  |  |  |  |  |  |  |  |  |
| MPO | 3.20 | |  |  |  |  |  |  |  |  |  |  |  |
| BBB | 3.77 | |  |  |  |  |  |  |  |  |  |  |  |
| LogBB | -0.43 | |  |  |  |  |  |  |  |  |  |  |  |

IP = Isoelectronic Point; RB = Number of rotatable bonds; HLB = hydrophilic-lipophilic balance; logS = water solubility; PL = polarizability(Å^3^); Minimal Projection Area (Å^2^); MSA = molecular surface area; HBA = hydr ogen bond acceptors; MR = Molar refractivity; ClogD = distribution at pKa; PSA = polar surface area; HBD = hydrogen bond donors; pKa = estimated acid strength; MPO = score for CNS penetration; LogBB = blood-brain distribution (0.152ClogP – 0.0148PSA + 0.138); hERG = estimated pIC50 (pAct) value for hERG (the human ether-a-go-go (hERG) ion channel); hERG_AM_ = hERG activity model; hERG_CM_ = hERG classification model

| BBB Score Calculator | | |
| --- | --- | --- |
| Property | Value | T_0_ |
| ARO_R_SCORE | 3 | 0.69 |
| HA_SCORE | 29 | 0.79 |
| HWHBN_SCORE | 0.35 | 0.69 |
| TPSA_SCORE | 88.52 | 0.38 |
| PKA_SCORE | 8.81 | 1.00 |
| BBB_SCORE | | 3.77 |

ARO_R_SCORE = Number of aromatic rings; HA_SCORE = Number of heavy atoms. MWHNM_SCORE = [-0.5MW(HBA+HBD)] where MW = molecular weight, HBA = number of hydrogen bond acceptor sites, and HBD = number of hydrogen bond donors; TPSA = topological polar surface area; PKA_SCORE = Acidity strength. Gupta, M.; Lee, H. J.; Barden, C. J.; Weaver, D. “The Blood-Brain Barrier (BBB) Score” J. Med. Chem. 2019, 62 (21), 9824-9836.

| Calc. | MarvinSketch | | IP | RB | HLB | logS | PL | MPA | MSA | HBA | MR | hERG_AM_ | hERG_CM_ |
| --- | --- | --- | --- | --- | --- | --- | --- | --- | --- | --- | --- | --- | --- |
|  |  | T0 | 6.13 | 6 | 11.51 | -6.13 | 42.26 | 43.33 | 558.19 | 9 | 112.63 | 5.36 | safe |
| ClogP | 3.31 | 0.84 |  |  |  |  |  |  |  |  |  |  |  |
| ClogD | 3.31 | 0.84 |  |  |  |  |  |  |  |  |  |  |  |
| MW | 410.85 | 0.64 |  |  |  |  |  |  |  |  |  |  |  |
| PSA | 82.34 | 1.00 |  |  |  |  |  |  |  |  |  |  |  |
| HBD | 1.00 | 0.75 |  |  |  |  |  |  |  |  |  |  |  |
| pKa | 0.81 | 1.00 |  |  |  |  |  |  |  |  |  |  |  |
| MPO | 4.57 | |  |  |  |  |  |  |  |  |  |  |  |
| BBB | 4.28 | |  |  |  |  |  |  |  |  |  |  |  |
| LogBB |  | |  |  |  |  |  |  |  |  |  |  |  |

IP = Isoelectronic Point; RB = Number of rotatable bonds; HLB = hydrophilic-lipophilic balance; logS = water solubility; PL = polarizability(Å^3^); Minimal Projection Area (Å^2^); MSA = molecular surface area; HBA = hydr ogen bond acceptors; MR = Molar refractivity; ClogD = distribution at pKa; PSA = polar surface area; HBD = hydrogen bond donors; pKa = estimated acid strength; MPO = score for CNS penetration; LogBB = blood-brain distribution (0.152ClogP – 0.0148PSA + 0.138); hERG = estimated pIC50 (pAct) value for hERG (the human ether-a-go-go (hERG) ion channel); hERG_AM_ = hERG activity model; hERG_CM_ = hERG classification model

| BBB Score Calculator | | |
| --- | --- | --- |
| Property | Value | T_0_ |
| ARO_R_SCORE | 3 | 0.69 |
| HA_SCORE | 29 | 0.79 |
| HWHBN_SCORE | 0.25 | 0.97 |
| TPSA_SCORE | 82.34 | 0.43 |
| PKA_SCORE | 8.81 | 1.00 |
| BBB_SCORE | | 4.28 |

ARO_R_SCORE = Number of aromatic rings; HA_SCORE = Number of heavy atoms. MWHNM_SCORE = [-0.5MW(HBA+HBD)] where MW = molecular weight, HBA = number of hydrogen bond acceptor sites, and HBD = number of hydrogen bond donors; TPSA = topological polar surface area; PKA_SCORE = Acidity strength. Gupta, M.; Lee, H. J.; Barden, C. J.; Weaver, D. “The Blood-Brain Barrier (BBB) Score” J. Med. Chem. 2019, 62 (21), 9824-9836.

| Calc. | MarvinSketch | | IP | RB | HLB | logS | PL | MPA | MSA | HBA | MR | hERG_AM_ | hERG_CM_ |
| --- | --- | --- | --- | --- | --- | --- | --- | --- | --- | --- | --- | --- | --- |
|  |  | T0 | 5.82 | 6 | 11.77 | -5.06 | 42.41 | 51.70 | 556.21 | 9 | 109.30 | 5.63 | safe |
| ClogP | 4.21 | 0.39 |  |  |  |  |  |  |  |  |  |  |  |
| ClogD | 4.06 | 0.00 |  |  |  |  |  |  |  |  |  |  |  |
| MW | 410.85 | 0.64 |  |  |  |  |  |  |  |  |  |  |  |
| PSA | 79.73 | 1.00 |  |  |  |  |  |  |  |  |  |  |  |
| HBD | 1.00 | 0.75 |  |  |  |  |  |  |  |  |  |  |  |
| pKa | 3.84 | 1.00 |  |  |  |  |  |  |  |  |  |  |  |
| MPO | 3.78 | |  |  |  |  |  |  |  |  |  |  |  |
| BBB | 4.11 | |  |  |  |  |  |  |  |  |  |  |  |
| LogBB |  | |  |  |  |  |  |  |  |  |  |  |  |

IP = Isoelectronic Point; RB = Number of rotatable bonds; HLB = hydrophilic-lipophilic balance; logS = water solubility; PL = polarizability(Å^3^); Minimal Projection Area (Å^2^); MSA = molecular surface area; HBA = hydr ogen bond acceptors; MR = Molar refractivity; ClogD = distribution at pKa; PSA = polar surface area; HBD = hydrogen bond donors; pKa = estimated acid strength; MPO = score for CNS penetration; LogBB = blood-brain distribution (0.152ClogP – 0.0148PSA + 0.138); hERG = estimated pIC50 (pAct) value for hERG (the human ether-a-go-go (hERG) ion channel); hERG_AM_ = hERG activity model; hERG_CM_ = hERG classification model

| BBB Score Calculator | | |
| --- | --- | --- |
| Property | Value | T_0_ |
| ARO_R_SCORE | 3.00 | 0.69 |
| HA_SCORE | 29.00 | 0.79 |
| HWHBN_SCORE | 0.30 | 0.85 |
| TPSA_SCORE | 79.73 | 0.44 |
| PKA_SCORE | 7.79 | 0.94 |
| BBB_SCORE | | 4.11 |

ARO_R_SCORE = Number of aromatic rings; HA_SCORE = Number of heavy atoms. MWHNM_SCORE = [-0.5MW(HBA+HBD)] where MW = molecular weight, HBA = number of hydrogen bond acceptor sites, and HBD = number of hydrogen bond donors; TPSA = topological polar surface area; PKA_SCORE = Acidity strength. Gupta, M.; Lee, H. J.; Barden, C. J.; Weaver, D. “The Blood-Brain Barrier (BBB) Score” J. Med. Chem. 2019, 62 (21), 9824-9836.

| Calc. | MarvinSketch | | IP | RB | HLB | logS | PL | MPA | MSA | HBA | MR | hERG_AM_ | hERG_CM_ |
| --- | --- | --- | --- | --- | --- | --- | --- | --- | --- | --- | --- | --- | --- |
|  |  | T0 | 8.35 | 7 | 12.06 | -5.44 | 42.41 | 40.63 | 553.24 | 9 | 110.86 | 5.40 | safe |
| ClogP | 3.53 | 0.74 |  |  |  |  |  |  |  |  |  |  |  |
| ClogD | 3.53 | 0.24 |  |  |  |  |  |  |  |  |  |  |  |
| MW | 410.85 | 0.64 |  |  |  |  |  |  |  |  |  |  |  |
| PSA | 88.52 | 1.00 |  |  |  |  |  |  |  |  |  |  |  |
| HBD | 2.00 | 0.50 |  |  |  |  |  |  |  |  |  |  |  |
| pKa | 4.37 | 1.00 |  |  |  |  |  |  |  |  |  |  |  |
| MPO | 4.11 | |  |  |  |  |  |  |  |  |  |  |  |
| BBB | 3.77 | |  |  |  |  |  |  |  |  |  |  |  |
| LogBB |  | |  |  |  |  |  |  |  |  |  |  |  |

IP = Isoelectronic Point; RB = Number of rotatable bonds; HLB = hydrophilic-lipophilic balance; logS = water solubility; PL = polarizability(Å^3^); Minimal Projection Area (Å^2^); MSA = molecular surface area; HBA = hydr ogen bond acceptors; MR = Molar refractivity; ClogD = distribution at pKa; PSA = polar surface area; HBD = hydrogen bond donors; pKa = estimated acid strength; MPO = score for CNS penetration; LogBB = blood-brain distribution (0.152ClogP – 0.0148PSA + 0.138); hERG = estimated pIC50 (pAct) value for hERG (the human ether-a-go-go (hERG) ion channel); hERG_AM_ = hERG activity model; hERG_CM_ = hERG classification model

| BBB Score Calculator | | |
| --- | --- | --- |
| Property | Value | T_0_ |
| ARO_R_SCORE | 3 | 0.69 |
| HA_SCORE | 29.00 | 0.79 |
| HWHBN_SCORE | 0.35 | 0.69 |
| TPSA_SCORE | 88.52 | 0.38 |
| PKA_SCORE | 8.81 | 1.00 |
| BBB_SCORE | | 3.77 |

ARO_R_SCORE = Number of aromatic rings; HA_SCORE = Number of heavy atoms. MWHNM_SCORE = [-0.5MW(HBA+HBD)] where MW = molecular weight, HBA = number of hydrogen bond acceptor sites, and HBD = number of hydrogen bond donors; TPSA = topological polar surface area; PKA_SCORE = Acidity strength. Gupta, M.; Lee, H. J.; Barden, C. J.; Weaver, D. “The Blood-Brain Barrier (BBB) Score” J. Med. Chem. 2019, 62 (21), 9824-9836.

| Calc. | MarvinSketch | | IP | RB | HLB | logS | PL | MPA | MSA | HBA | MR | hERG_AM_ | hERG_CM_ |
| --- | --- | --- | --- | --- | --- | --- | --- | --- | --- | --- | --- | --- | --- |
|  |  | T0 | 6.10 | 6 | 12.02 | -5.44 | 42.42 | 40.55 | 552.87 | 9 | 11.29 | 5.76 | safe |
| ClogP | 4.27 | 0.36 |  |  |  |  |  |  |  |  |  |  |  |
| ClogD | 4.14 | 0.00 |  |  |  |  |  |  |  |  |  |  |  |
| MW | 410.85 | 0.64 |  |  |  |  |  |  |  |  |  |  |  |
| PSA | 88.52 | 1.00 |  |  |  |  |  |  |  |  |  |  |  |
| HBD | 2.00 | 0.50 |  |  |  |  |  |  |  |  |  |  |  |
| pKa | 4.37 | 1.00 |  |  |  |  |  |  |  |  |  |  |  |
| MPO | 3.50 | |  |  |  |  |  |  |  |  |  |  |  |
| BBB | 3.75 | |  |  |  |  |  |  |  |  |  |  |  |
| LogBB |  | |  |  |  |  |  |  |  |  |  |  |  |

IP = Isoelectronic Point; RB = Number of rotatable bonds; HLB = hydrophilic-lipophilic balance; logS = water solubility; PL = polarizability(Å^3^); Minimal Projection Area (Å^2^); MSA = molecular surface area; HBA = hydr ogen bond acceptors; MR = Molar refractivity; ClogD = distribution at pKa; PSA = polar surface area; HBD = hydrogen bond donors; pKa = estimated acid strength; MPO = score for CNS penetration; LogBB = blood-brain distribution (0.152ClogP – 0.0148PSA + 0.138); hERG = estimated pIC50 (pAct) value for hERG (the human ether-a-go-go (hERG) ion channel); hERG_AM_ = hERG activity model; hERG_CM_ = hERG classification model

| BBB Score Calculator | | |
| --- | --- | --- |
| Property | Value | T_0_ |
| ARO_R_SCORE | 3.00 | 0.69 |
| HA_SCORE | 29.00 | 0.79 |
| HWHBN_SCORE | 0.35 | 0.69 |
| TPSA_SCORE | 88.52 | 0.38 |
| PKA_SCORE | 7.85 | 0.95 |
| BBB_SCORE | | 3.75 |

ARO_R_SCORE = Number of aromatic rings; HA_SCORE = Number of heavy atoms. MWHNM_SCORE = [-0.5MW(HBA+HBD)] where MW = molecular weight, HBA = number of hydrogen bond acceptor sites, and HBD = number of hydrogen bond donors; TPSA = topological polar surface area; PKA_SCORE = Acidity strength. Gupta, M.; Lee, H. J.; Barden, C. J.; Weaver, D. “The Blood-Brain Barrier (BBB) Score” J. Med. Chem. 2019, 62 (21), 9824-9836.

| Calc. | MarvinSketch | | IP | RB | HLB | logS | PL | MPA | MSA | HBA | MR | hERG_AM_ | hERG_CM_ |
| --- | --- | --- | --- | --- | --- | --- | --- | --- | --- | --- | --- | --- | --- |
|  |  | T0 | 8.37 | 6 | 11.34 | -4.95 | 42.17 | 53.36 | 554.15 | 7 | 109.84 | 5.49 | safe |
| ClogP | 4.13 | 0.43 |  |  |  |  |  |  |  |  |  |  |  |
| ClogD | 4.13 | 0.00 |  |  |  |  |  |  |  |  |  |  |  |
| MW | 396.87 | 0.74 |  |  |  |  |  |  |  |  |  |  |  |
| PSA | 71.45 | 1.00 |  |  |  |  |  |  |  |  |  |  |  |
| HBD | 2.00 | 0.50 |  |  |  |  |  |  |  |  |  |  |  |
| pKa | 4.37 | 1.00 |  |  |  |  |  |  |  |  |  |  |  |
| MPO | 3.67 | |  |  |  |  |  |  |  |  |  |  |  |
| BBB | 4.27 | |  |  |  |  |  |  |  |  |  |  |  |
| LogBB |  | |  |  |  |  |  |  |  |  |  |  |  |

IP = Isoelectronic Point; RB = Number of rotatable bonds; HLB = hydrophilic-lipophilic balance; logS = water solubility; PL = polarizability(Å^3^); Minimal Projection Area (Å^2^); MSA = molecular surface area; HBA = hydr ogen bond acceptors; MR = Molar refractivity; ClogD = distribution at pKa; PSA = polar surface area; HBD = hydrogen bond donors; pKa = estimated acid strength; MPO = score for CNS penetration; LogBB = blood-brain distribution (0.152ClogP – 0.0148PSA + 0.138); hERG = estimated pIC50 (pAct) value for hERG (the human ether-a-go-go (hERG) ion channel); hERG_AM_ = hERG activity model; hERG_CM_ = hERG classification model

| BBB Score Calculator | | |
| --- | --- | --- |
| Property | Value | T_0_ |
| ARO_R_SCORE | 3.00 | 0.69 |
| HA_SCORE | 28.00 | 0.82 |
| HWHBN_SCORE | 0.30 | 0.84 |
| TPSA_SCORE | 71.45 | 0.50 |
| PKA_SCORE | 8.81 | 1.00 |
| BBB_SCORE | | 4.27 |

ARO_R_SCORE = Number of aromatic rings; HA_SCORE = Number of heavy atoms. MWHNM_SCORE = [-0.5MW(HBA+HBD)] where MW = molecular weight, HBA = number of hydrogen bond acceptor sites, and HBD = number of hydrogen bond donors; TPSA = topological polar surface area; PKA_SCORE = Acidity strength. Gupta, M.; Lee, H. J.; Barden, C. J.; Weaver, D. “The Blood-Brain Barrier (BBB) Score” J. Med. Chem. 2019, 62 (21), 9824-9836.

***3. Density Functional Theory (DFT) with ωB97X-D/6-31G* computed molecular properties.***

DFT computed properties of aminopyridine derivatives **HR48**, **HR66**-**HR90**

| Structure | EPM (solid) | LUMO | E_LUMO_ | HOMO | E_LUMO_ | ΔE_FMO_ | DM |
| --- | --- | --- | --- | --- | --- | --- | --- |
|  | 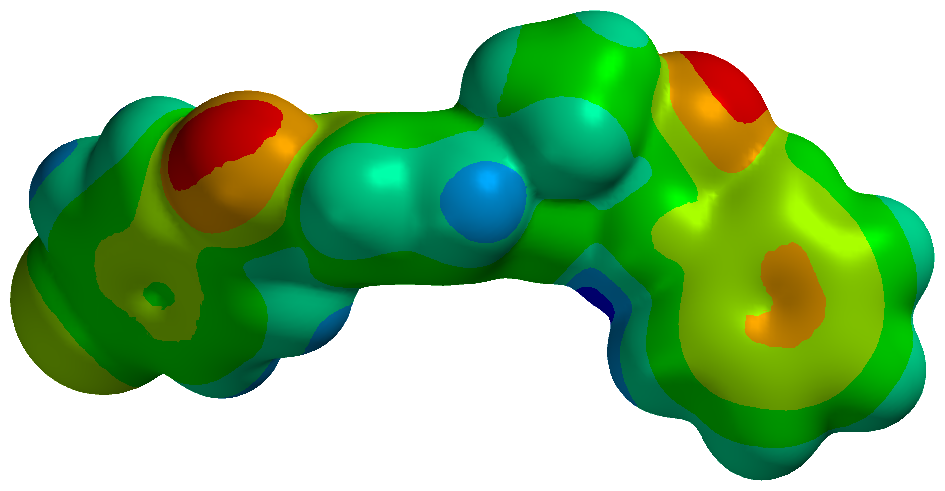 | 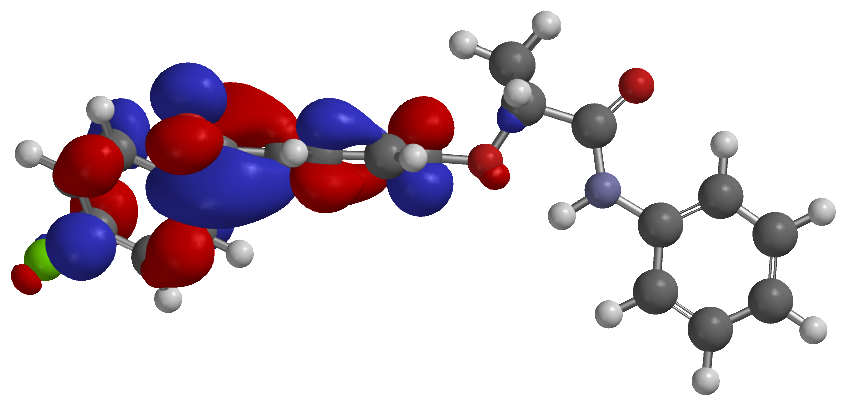 | -0.20 | 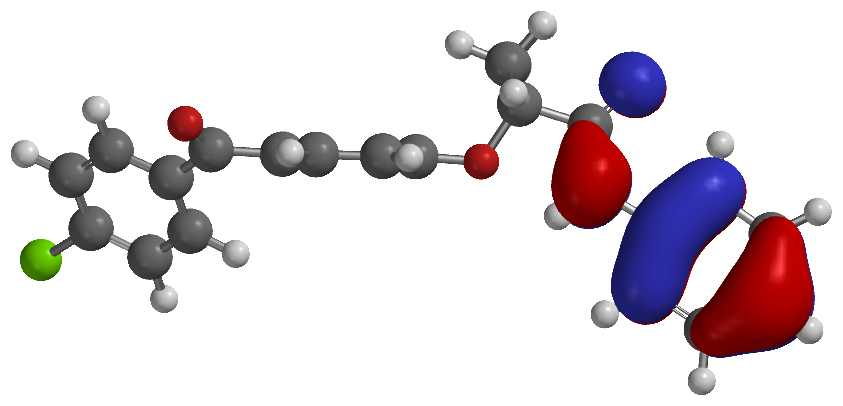 | -7.98 | 7.68 | 1.81 |
|  | 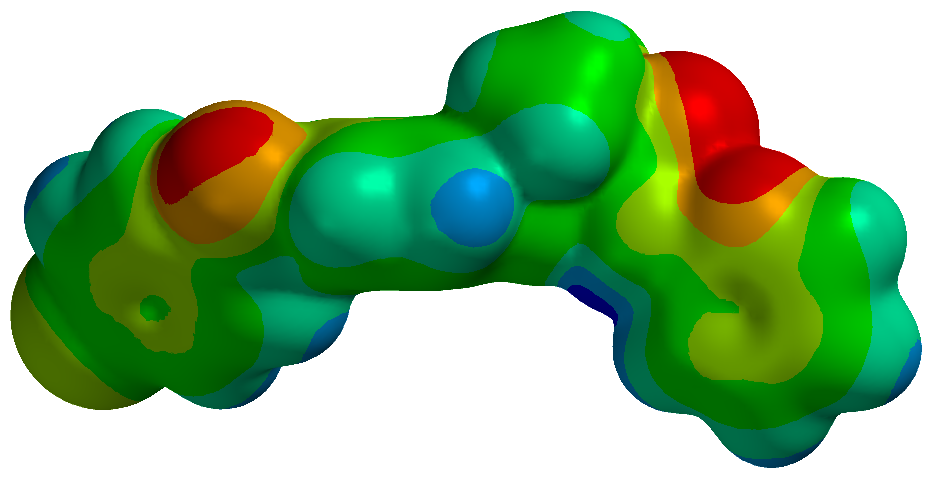 | 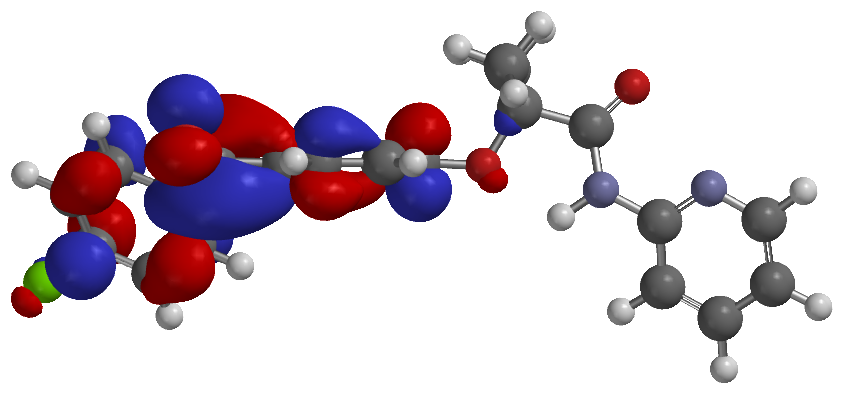 | -0.18 | 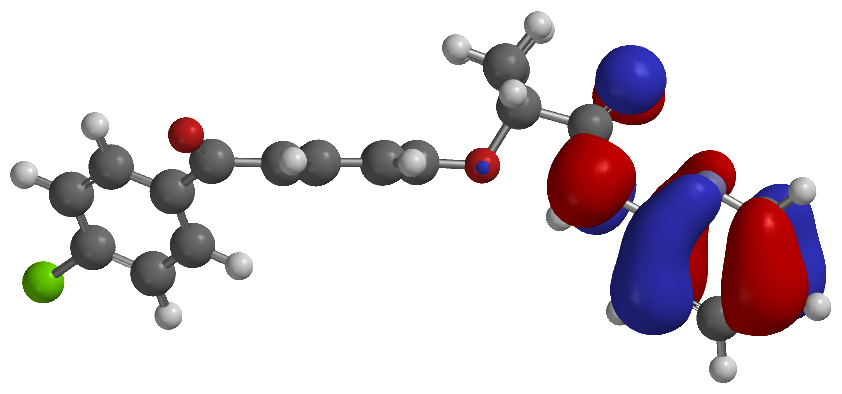 | -8.37 | 8.19 | 3.49 |
|  | 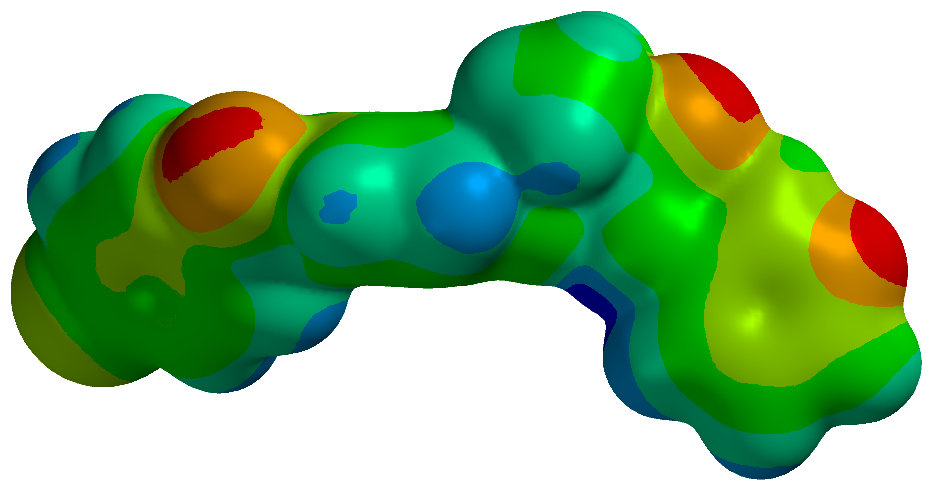 | 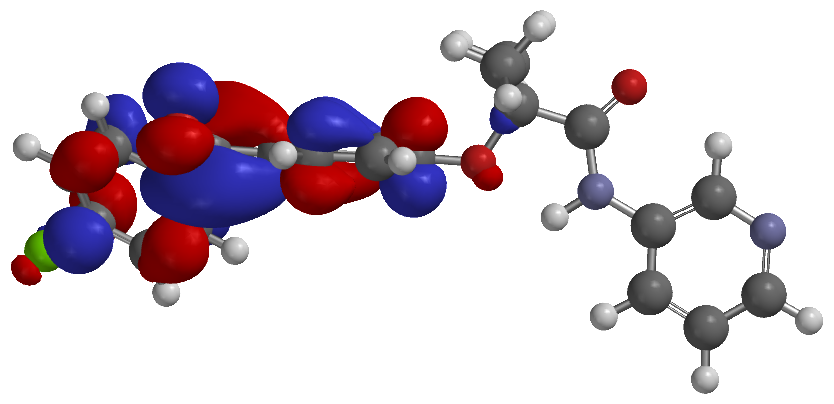 | -0.26 | 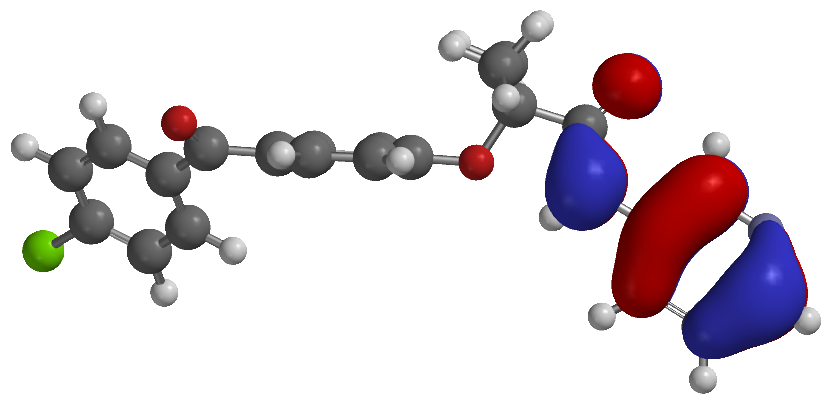 | -8.33 | 8.07 | 3.59 |
|  | 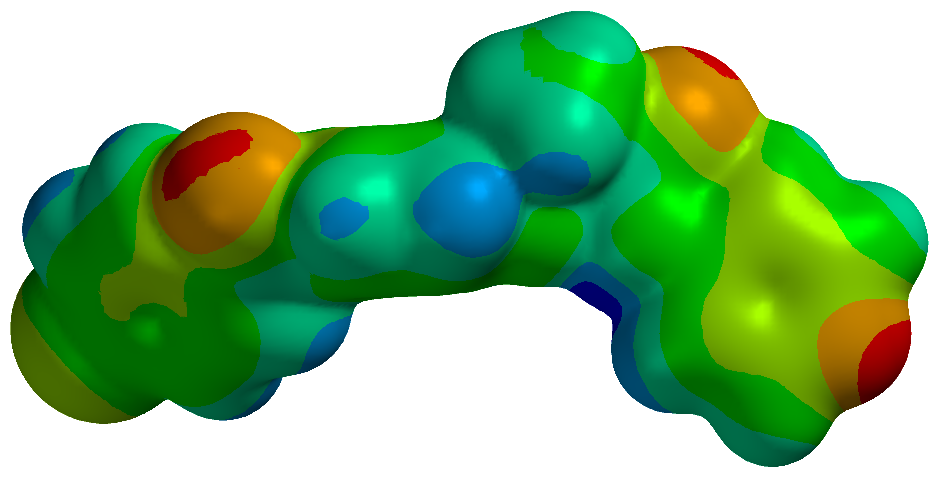 | 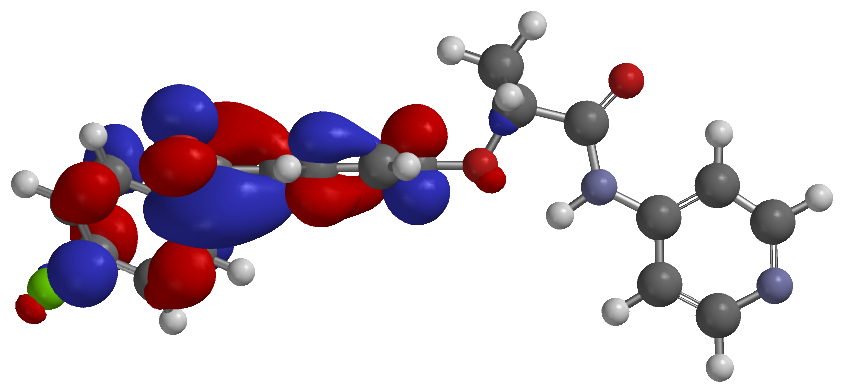 | -0.29 | 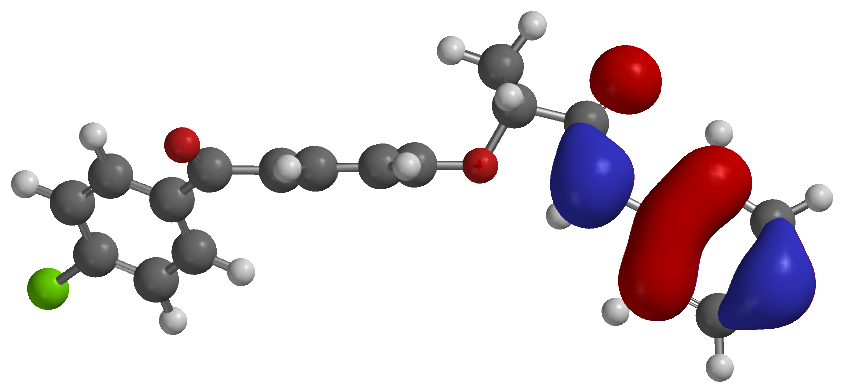 | -8.73 | 8.44 | 2.89 |
|  | 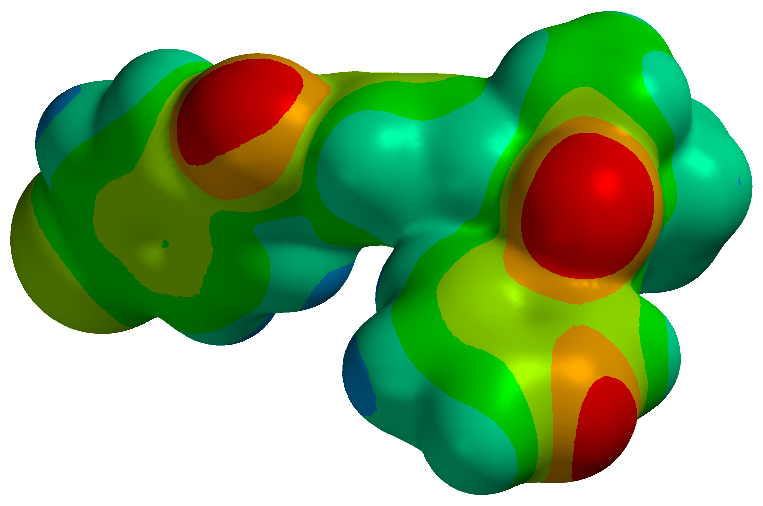 | 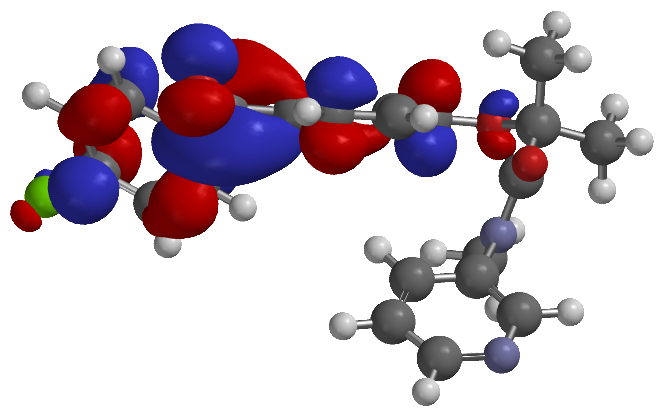 | -0.07 | 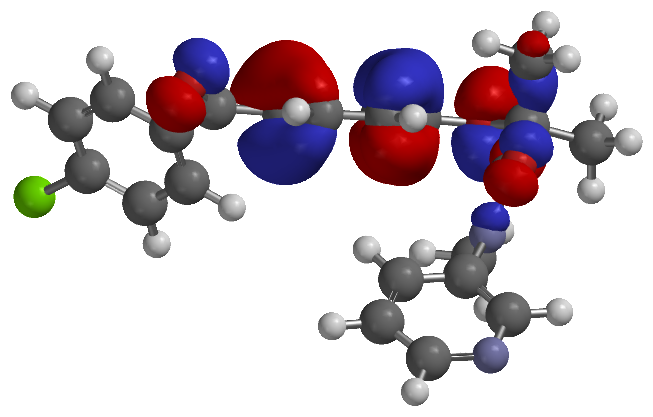 | -8.44 | 8.37 | 4.14 |
|  | 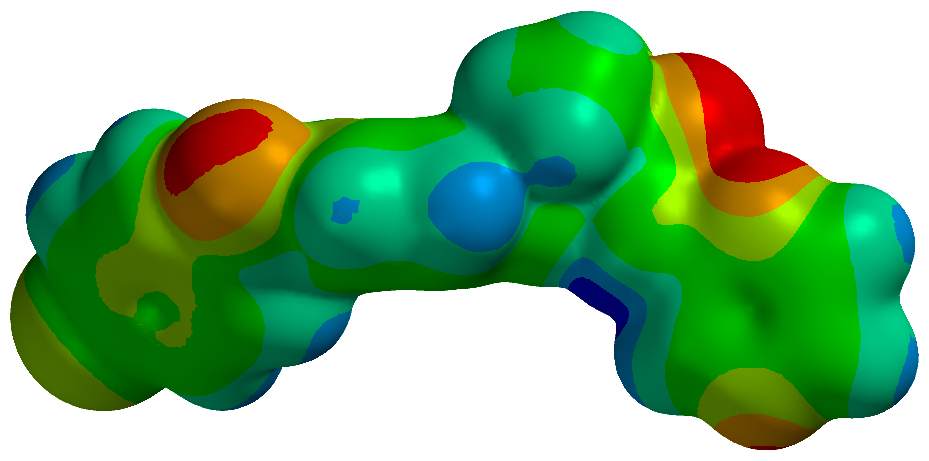 | 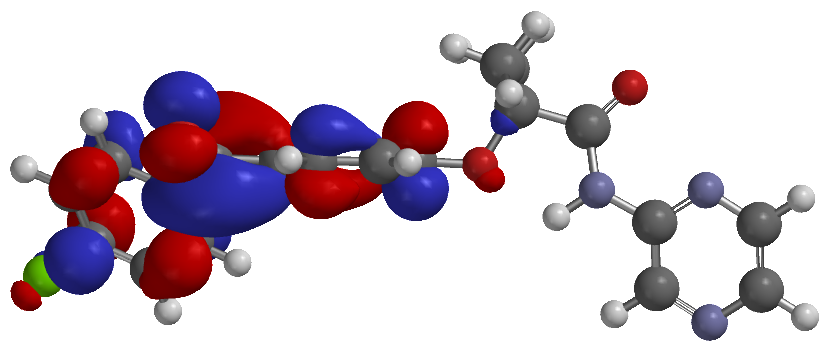 | -0.23 | 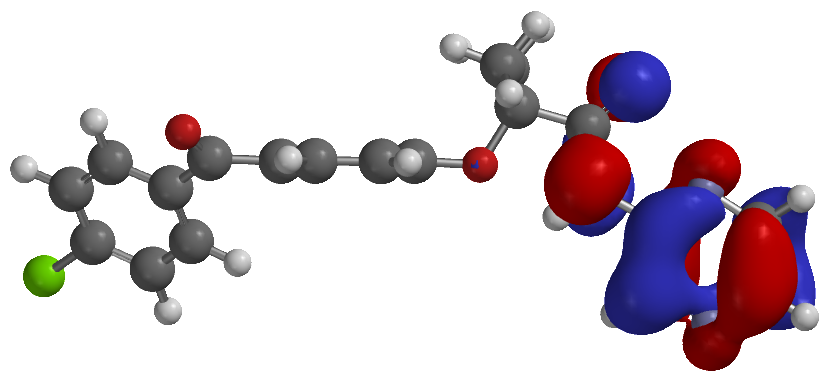 | -8.66 | 8.43 | 1.27 |
|  | 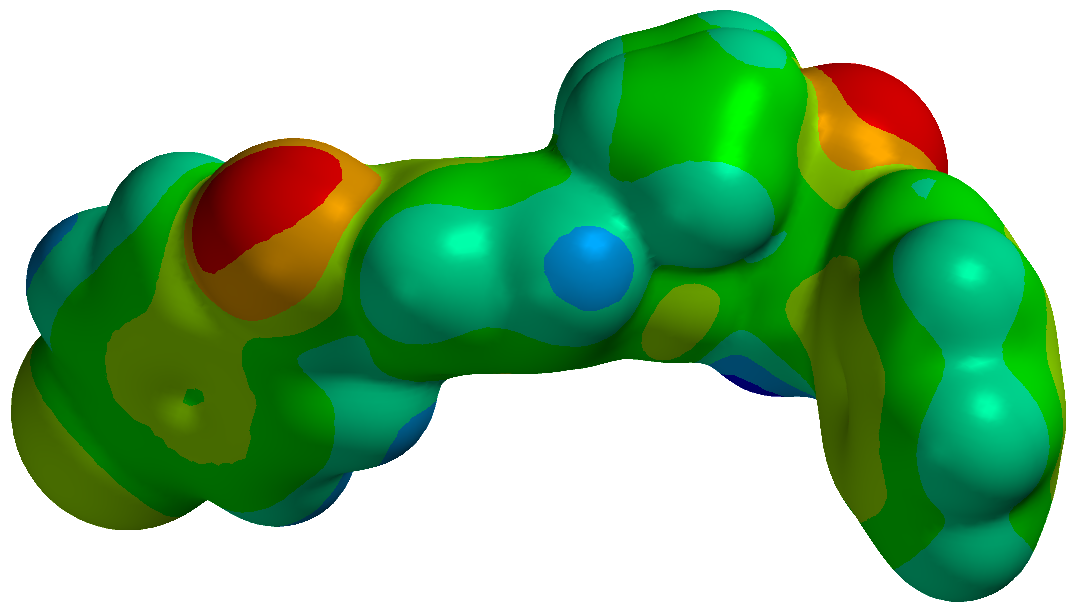 | 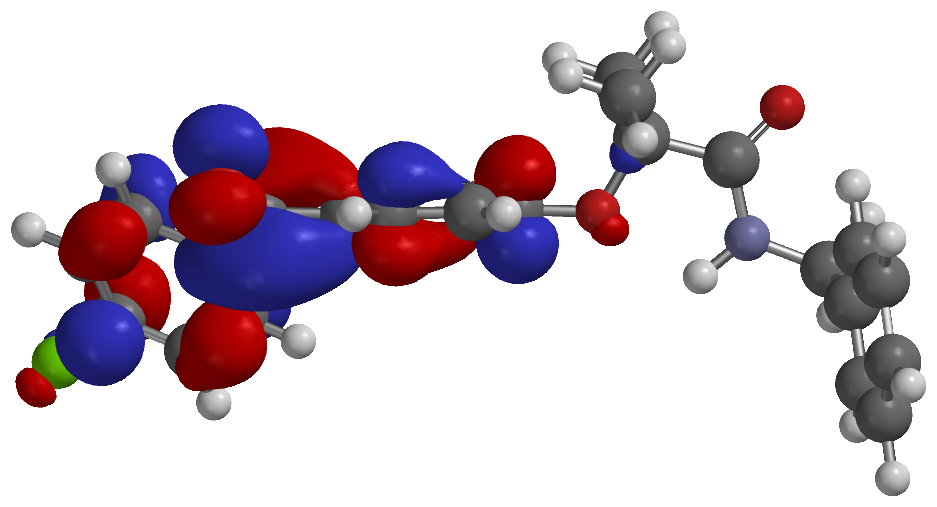 | -0.14 | 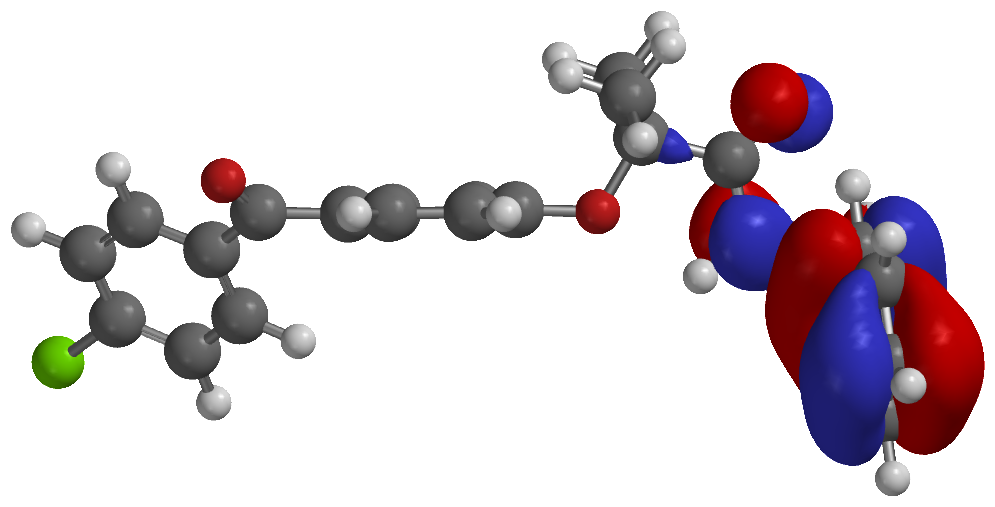 | -8.60 | 8.46 | 2.23 |
|  | 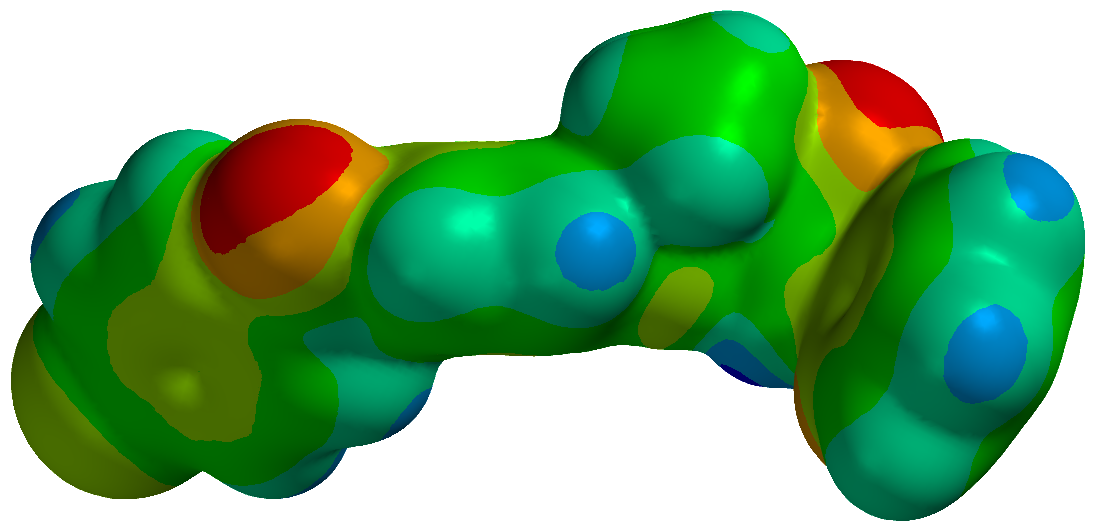 | 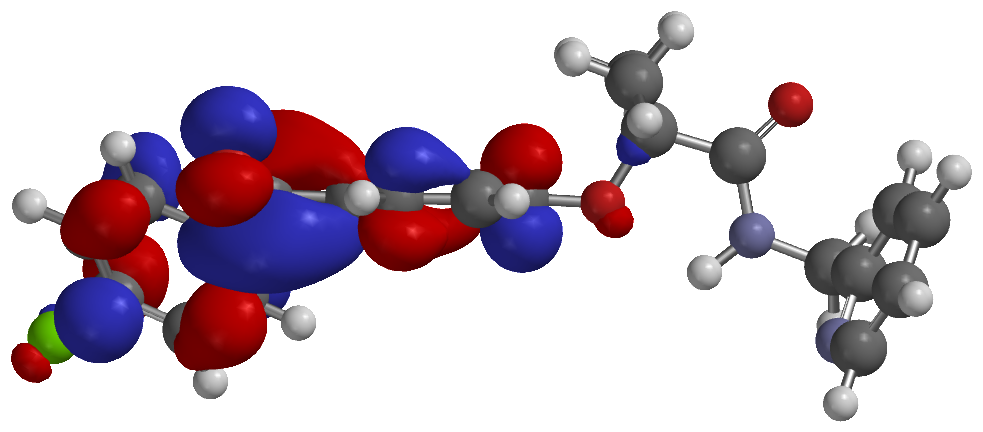 | -0.12 | 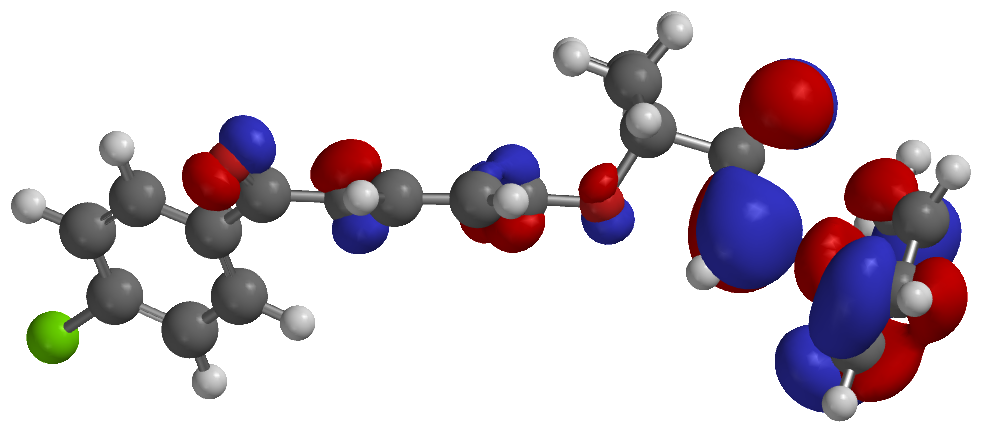 | -8.86 | 8.74 | 1.61 |
|  | 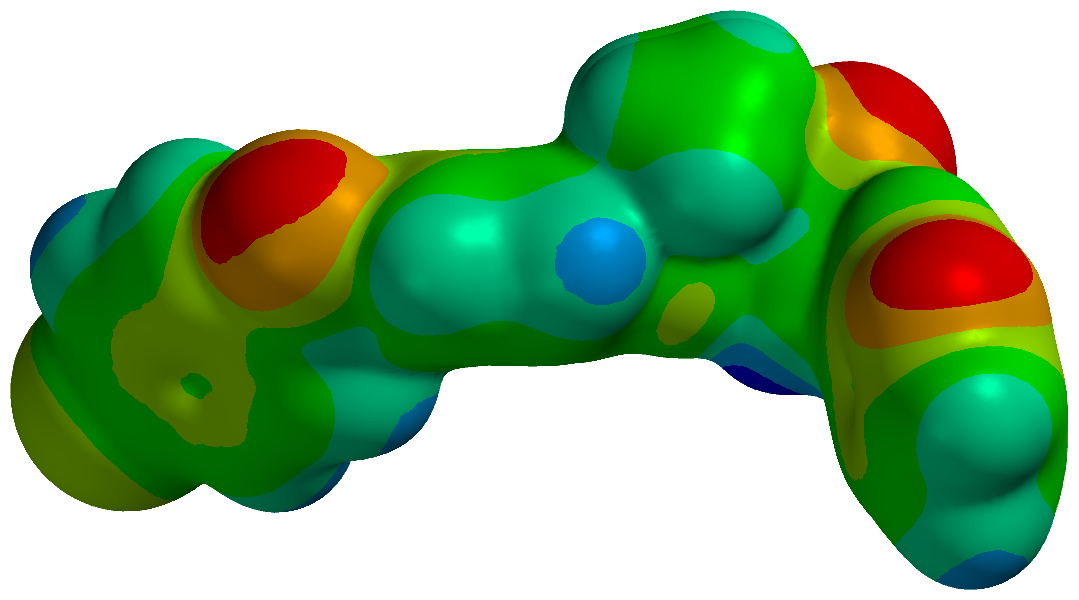 | 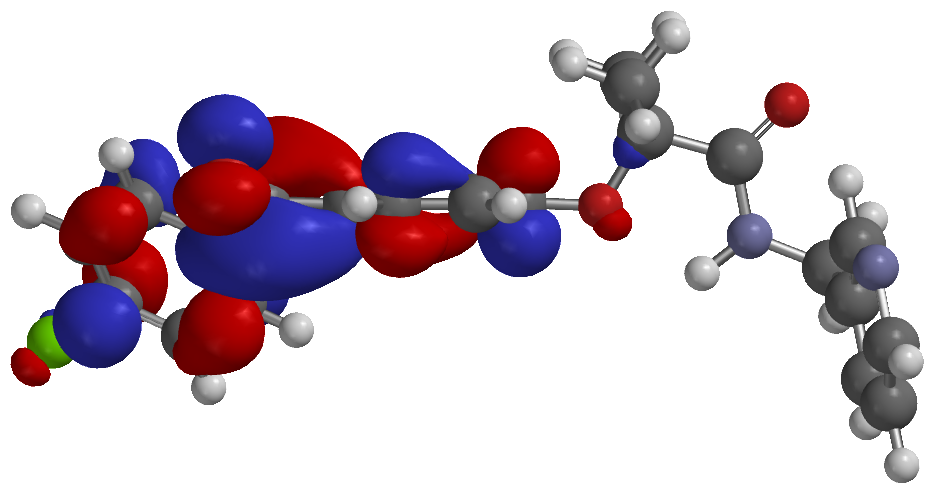 | -0.17 | 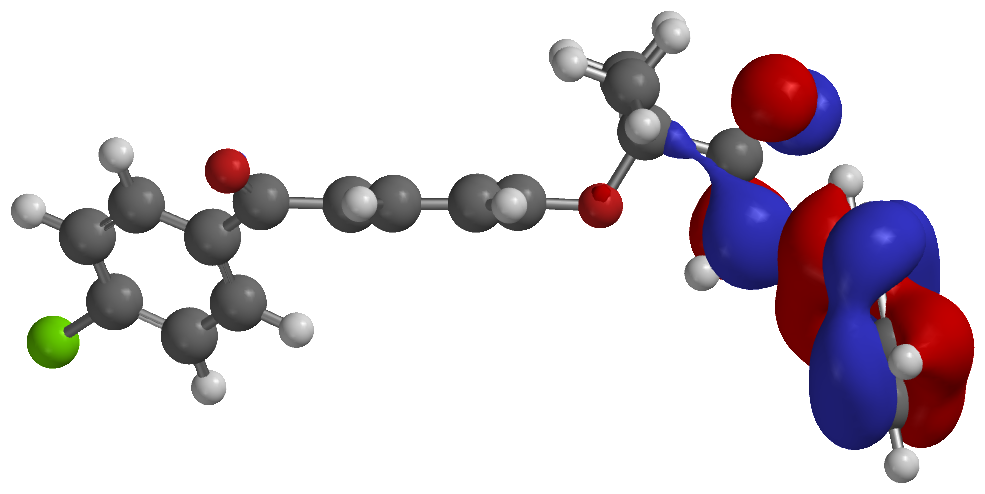 | -8.87 | 8.70 | 4.17 |
|  | 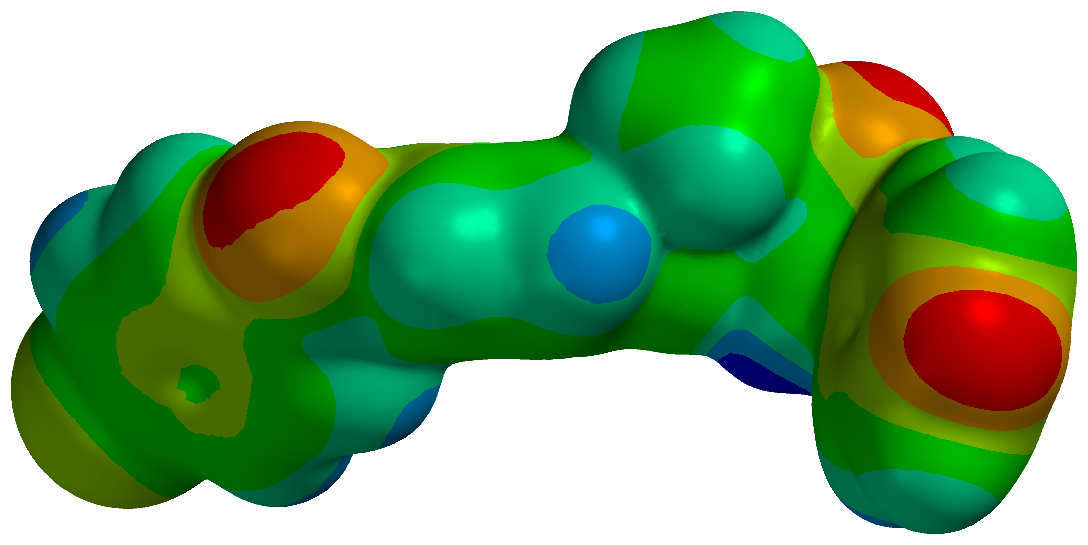 | 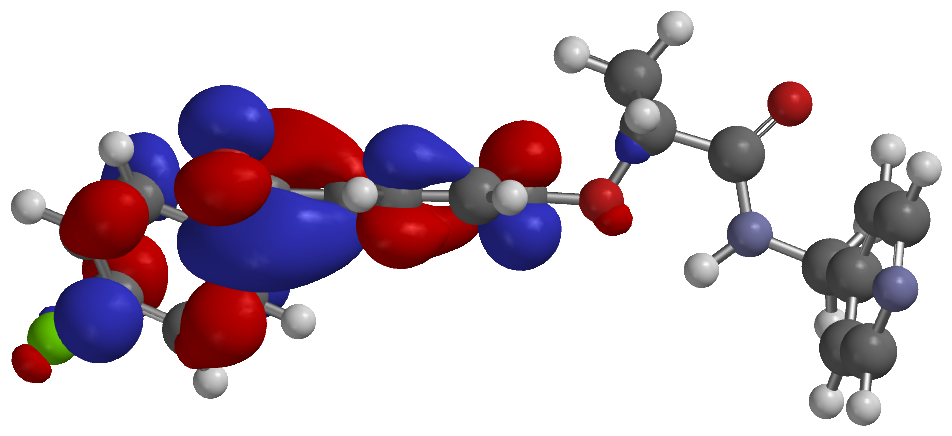 | -0.20 | 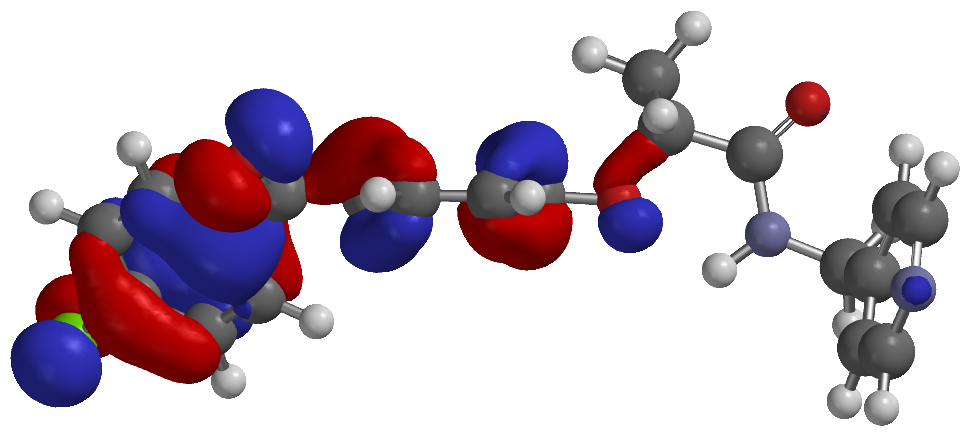 | -8.96 | 8.76 | 3.40 |
|  | 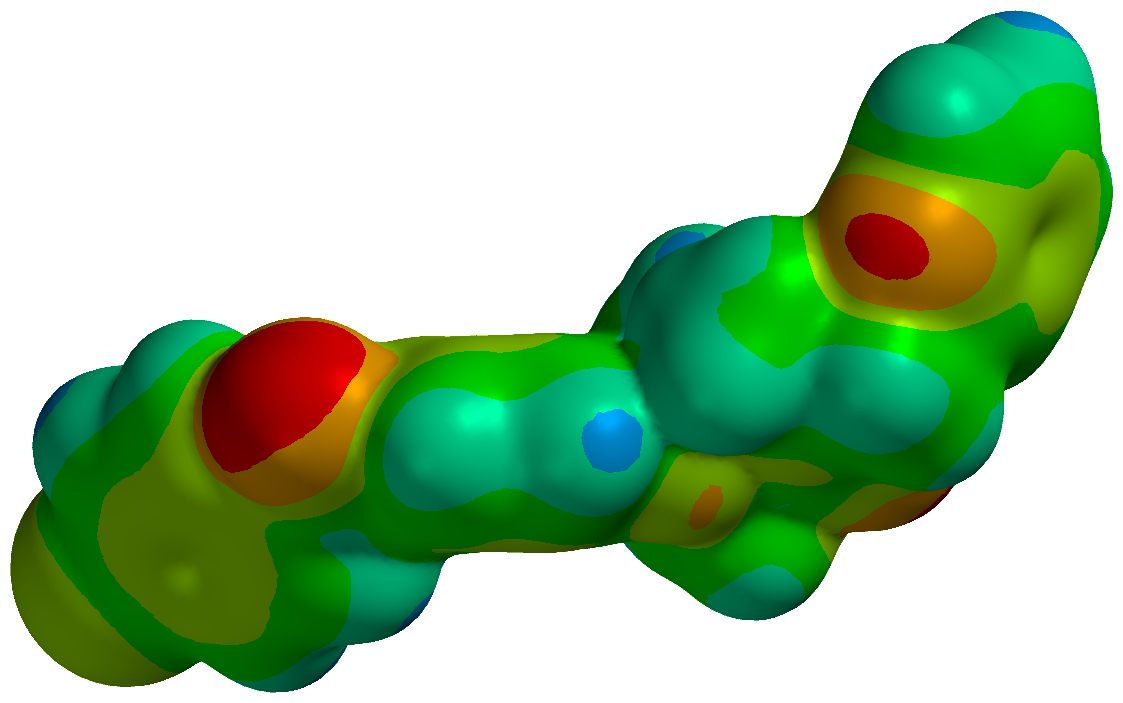 | 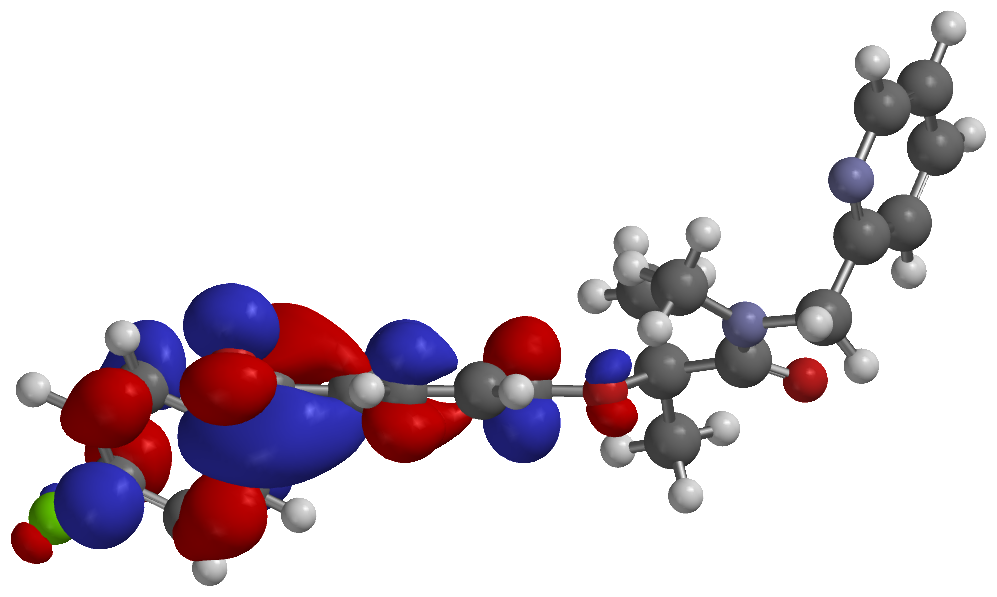 | 0.04 | 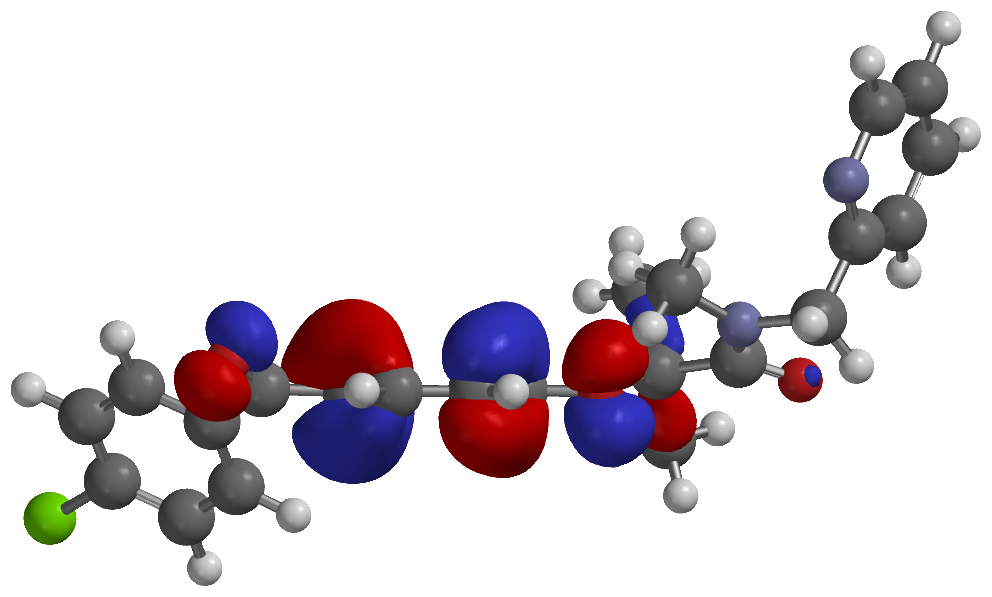 | -8.37 | 8.41 | 3.57 |
|  | 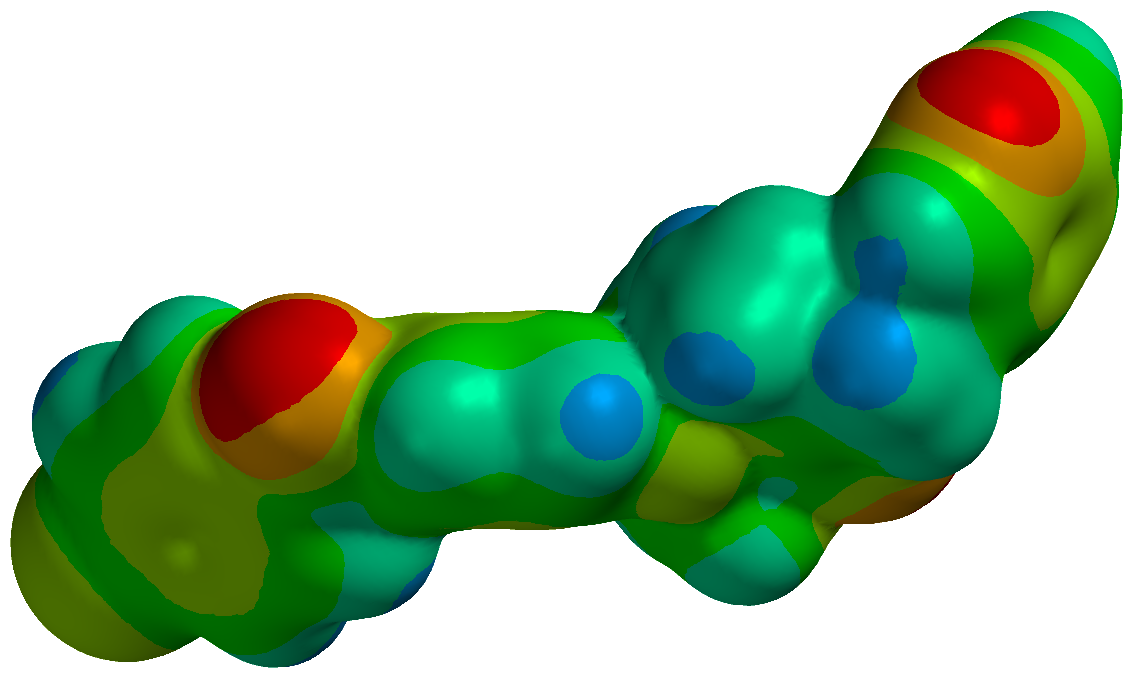 | 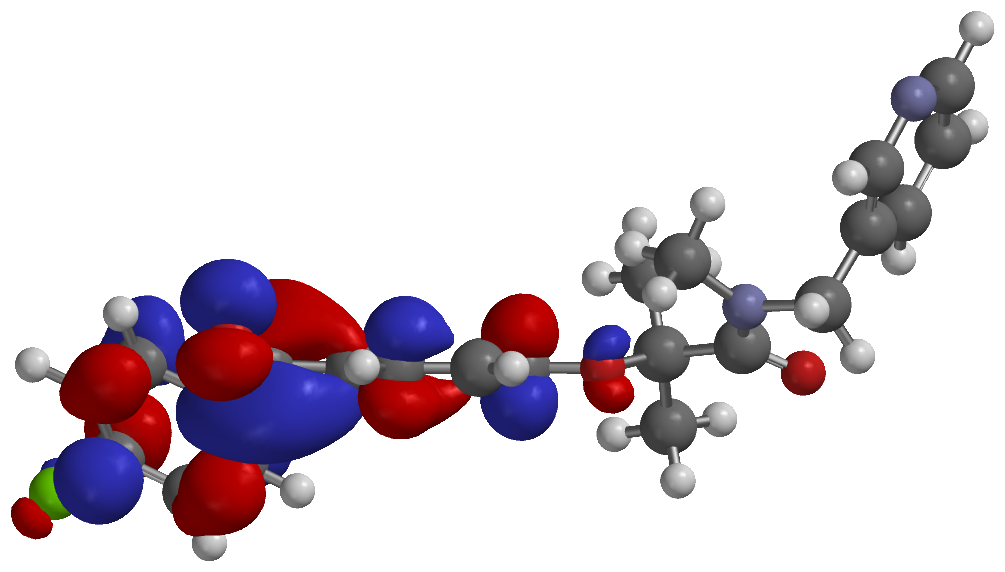 | -0.02 | 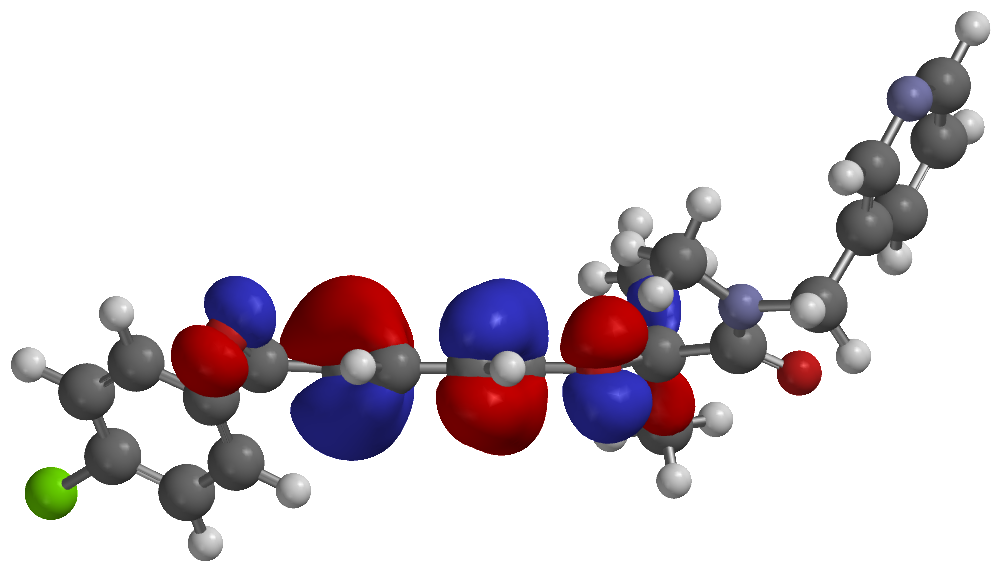 | -8.44 | 8.42 | 2.22 |
|  | 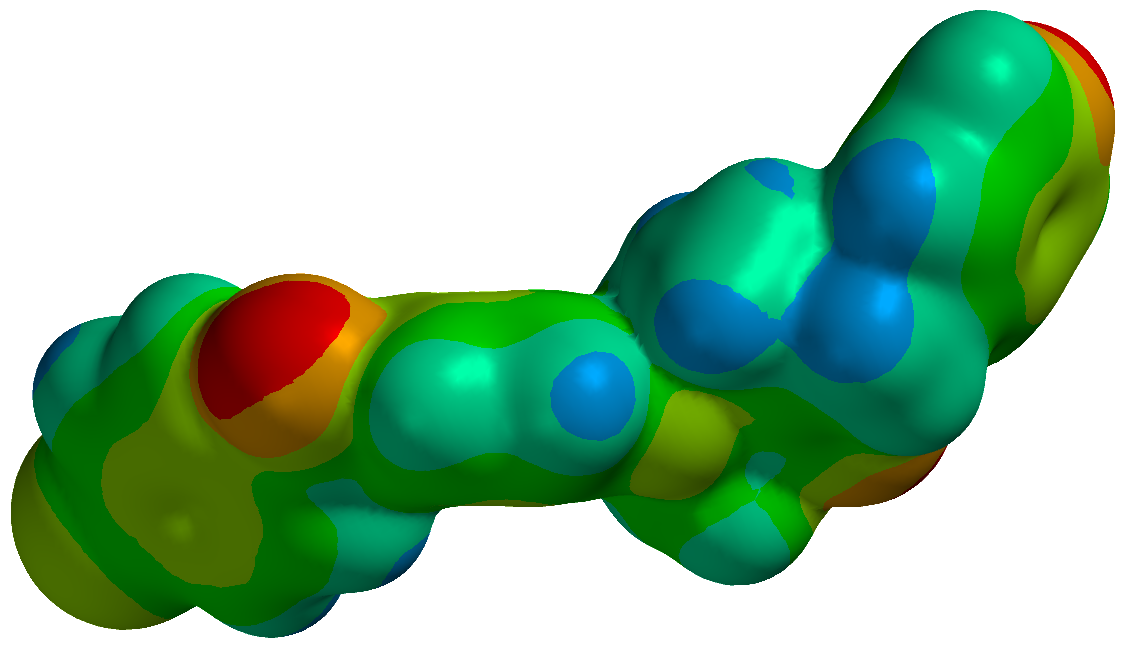 | 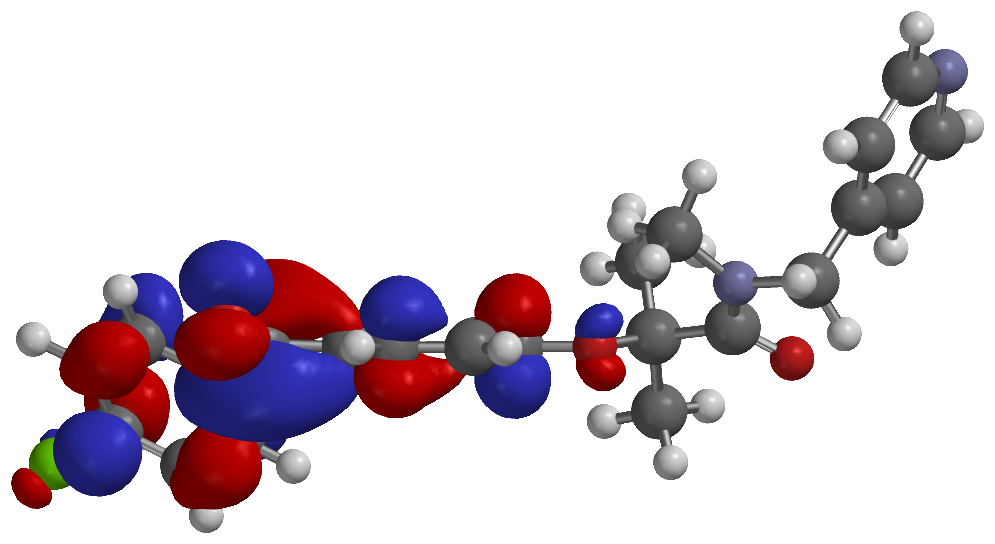 | -0.03 | 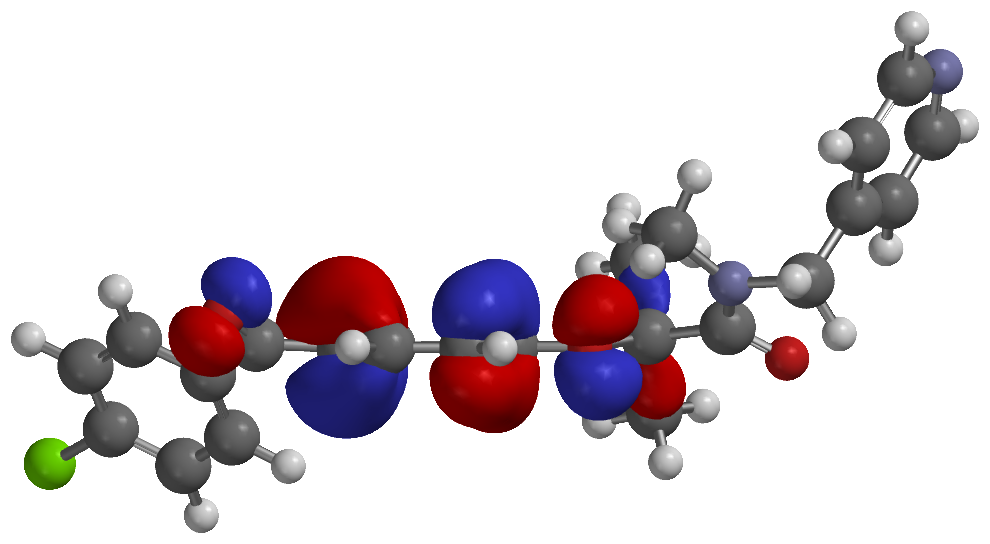 | -8.45 | 8.42 | 0.39 |
|  | 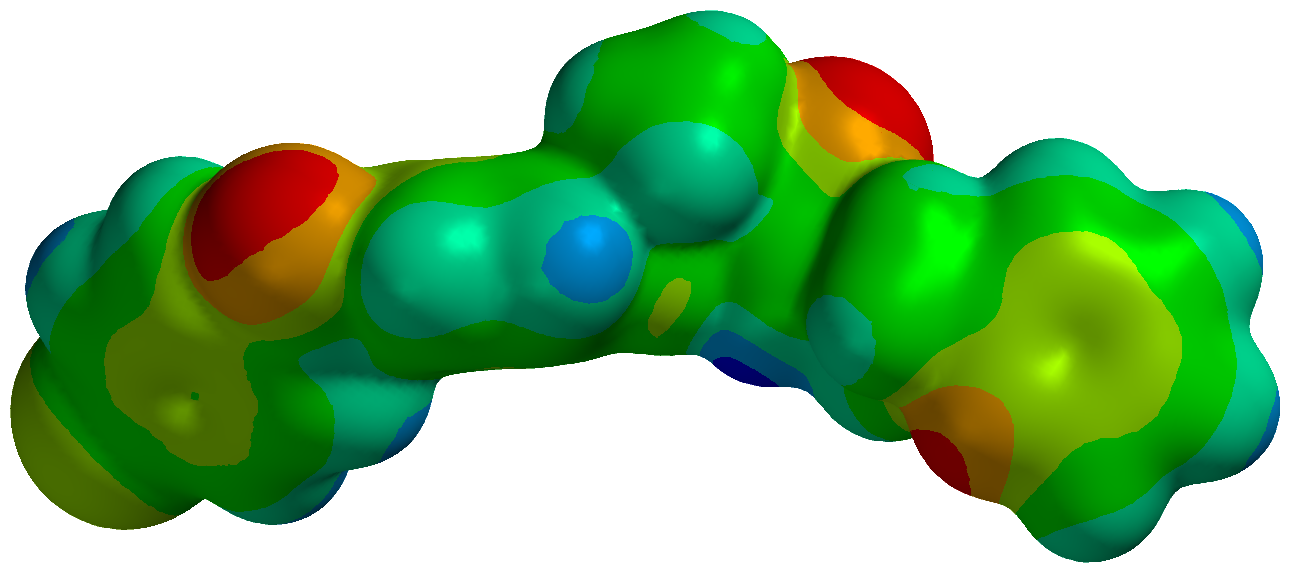 | 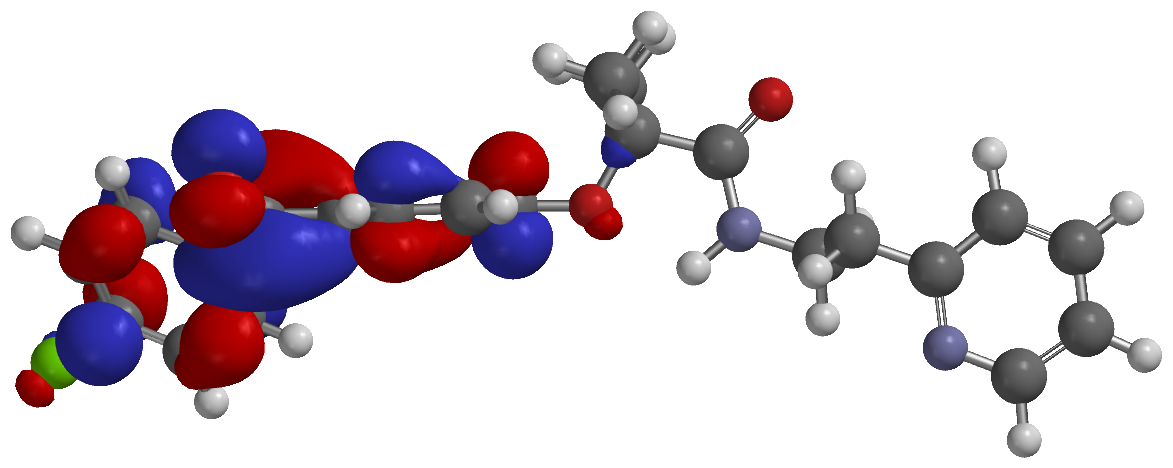 | -0.13 | 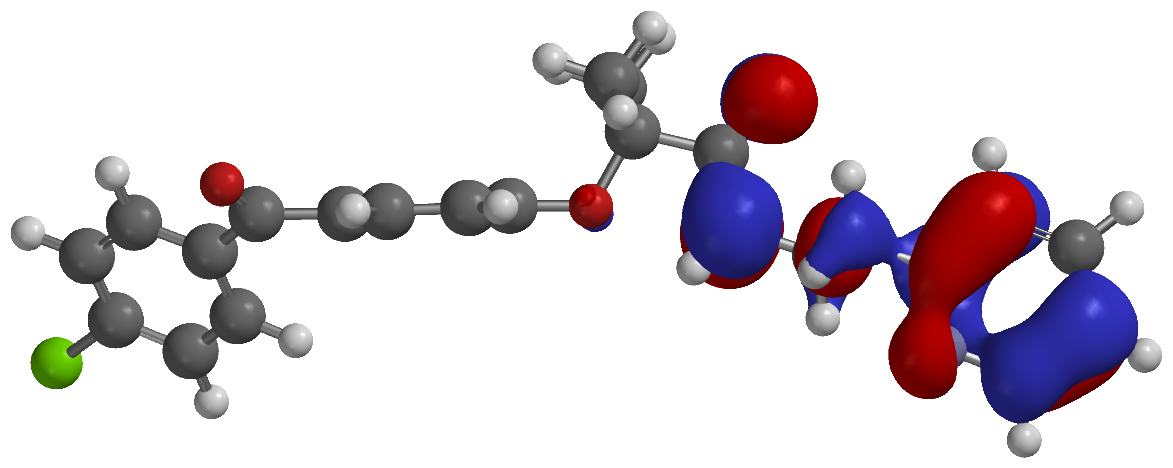 | -8.73 | 8.60 | 2.39 |
|  | 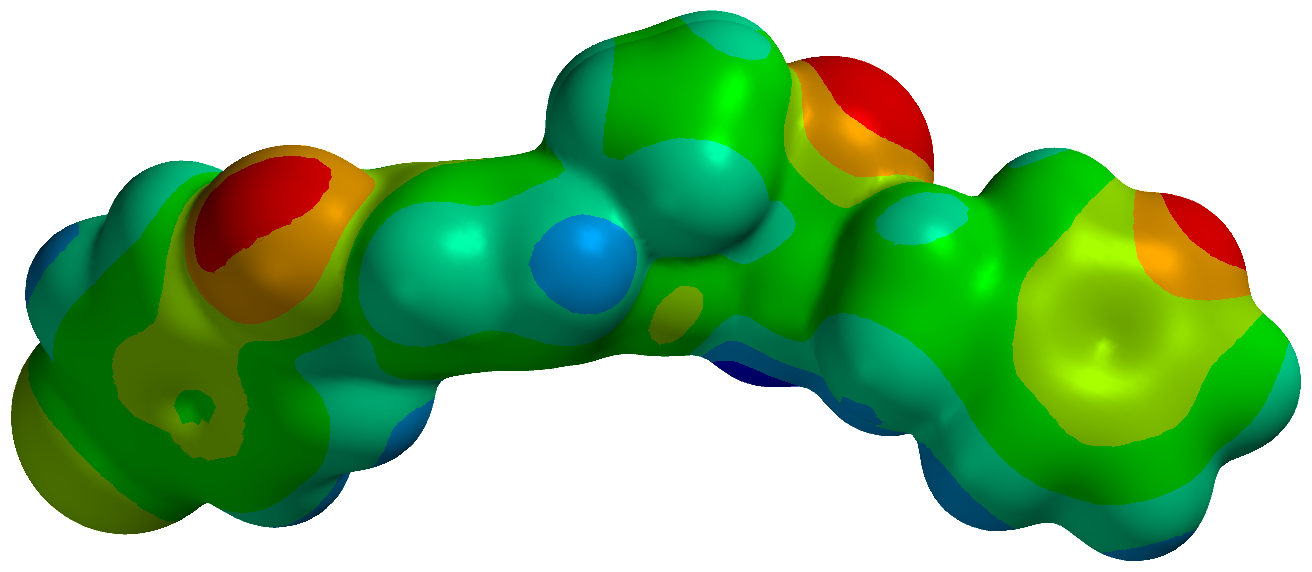 | 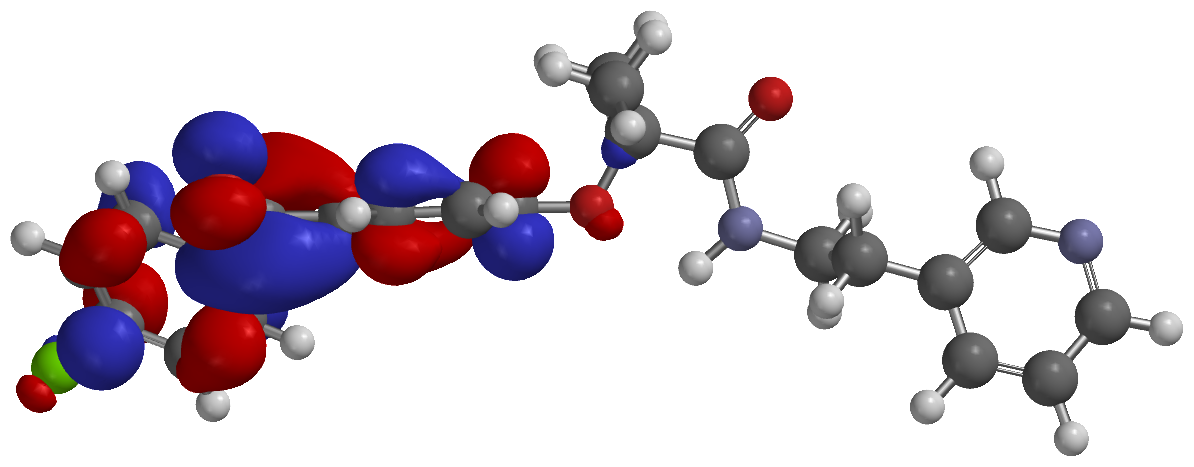 | -0.19 | 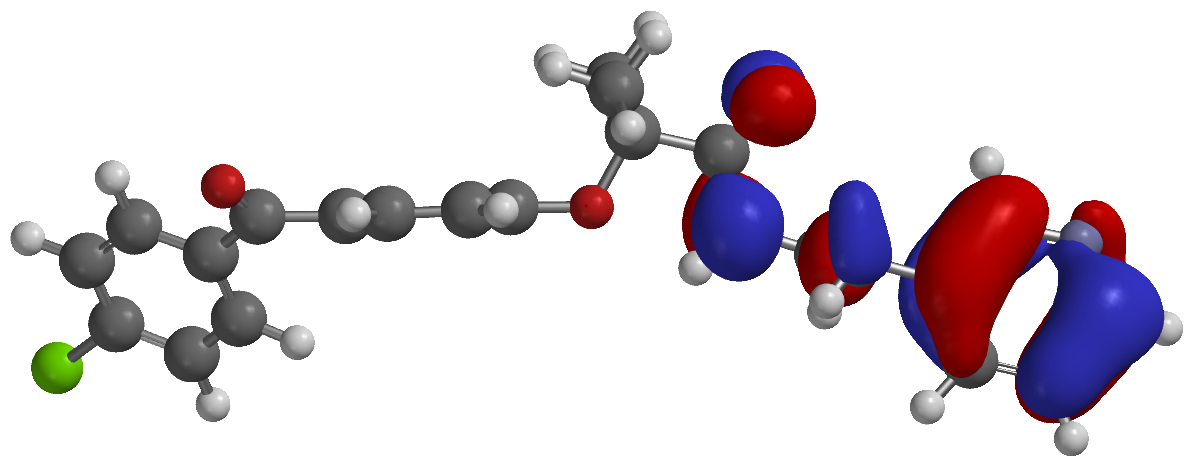 | -8.79 | 8.60 | 3.78 |
|  | 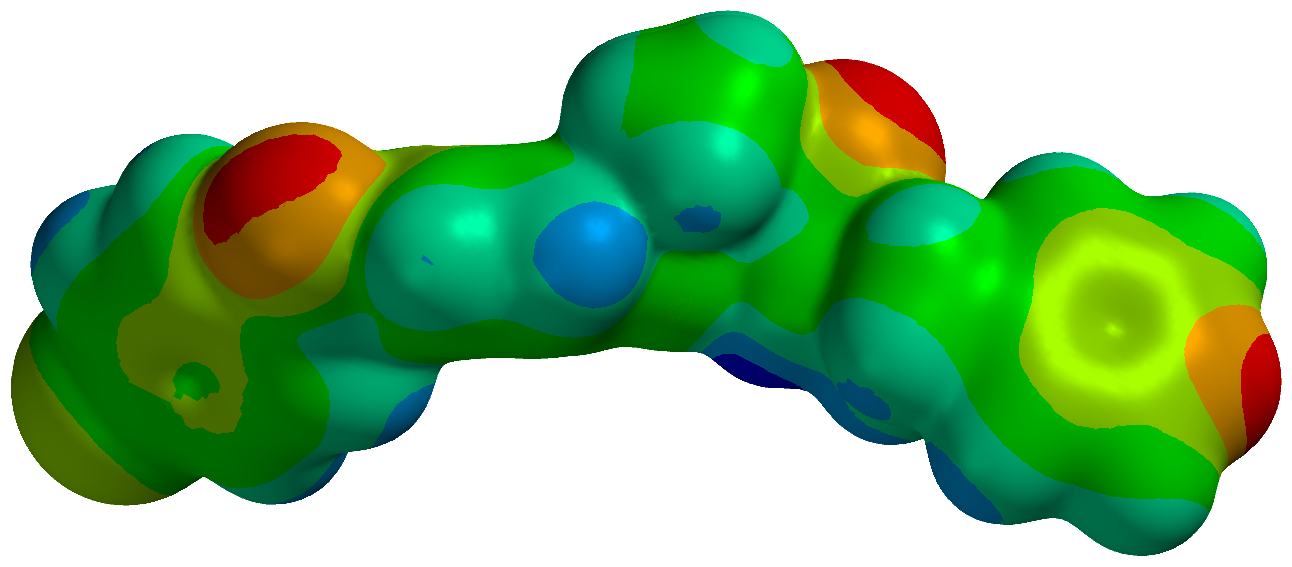 |  | -0.21 |  | -8.97 | 8.76 | 2.73 |
|  |  |  | 0.25 |  | -8.01 | 8.26 | 7.50 |
|  |  |  | -0.24 |  | -7.35 | 7.11 | 2.14 |
|  |  |  | -0.39 |  | -7.50 | 7.11 | 2.48 |
|  |  |  | -0.05 |  | -8.72 | 8.67 | 3.56 |
|  |  |  | -0.04 |  | -8.72 | 8.68 | 3.87 |
|  |  |  | -0.18 |  | -8.85 | 8.67 | 2.17 |
|  |  |  | -0.04 |  | -7.93 | 7.89 | 3.75 |
|  |  |  | -0.01 |  | -7.69 | 7.68 | 3.95 |
|  |  |  | -0.11 |  | -7.88 | 7.77 | 7.08 |

EPM = Electrostatic Potential Map; HOMO = Highest Occupied Molecular Orbital; LUMO = Lowest Unoccupied Molecular Orbital; E_HOMO_ = Energy of HOMO in eV; E_HOMO_ = Energy of LUMO in eV; ΔE_FMO_ = E_LUMO_-E_HOMO_ in eV; DP = dipole moment (Debye)

DFT (ωB97X-D/6-31G*) computed properties (nucleophilicity) of few selected anilines and aminopyridines.

| Nucleophyle | Structure | Electrostatic  Potential | HOMO | LUMO | HOMO (eV) | LUMO (eV) | Dipole Moment  (debye) |
| --- | --- | --- | --- | --- | --- | --- | --- |
| aniline |  |  |  |  | -7.12 | 2.29 | 1.89 |
| 4-aminophenol |  |  |  |  | -6.96 | 2.03 | 2.14 |
| 2-aminopyridine |  |  |  |  | -7.75 | 1.67 | 2.01 |
| 3-aminopydridine |  |  |  |  | -7.78 | 1.56 | 3.26 |
| 4-aminopyridine |  |  |  |  | -8.14 | 1.95 | 3.98 |

HOMO = Highest Occupied Molecular Orbital; LUMO = Lowest Unoccupied Molecular Orbital

1. ***Detection of our prototype drug candidate, PP1, in mouse tissues following intraperitoneal drug delivery.***

*Tissue distribution of our BPA-based prototype glioblastoma drug candidate, PP1. Mice* *were treated intraperitoneally (ip) with PP1 dissolved in 20% cyclodextrin at 50 mg/kg and the levels of the compound in blood, heart, liver, kidney, spleen, lung and brain were evaluated by HPLC (see methods). Data represent average values with standard deviation (n=3).*

1. ***Computed properties of BioTransformer 3.0 predicted phase I metabolites of HR67 (Panel A) and HR68 (Panel B).***

*A*

| Reaction type | Predicted Metabolite | hERG_AM_ | CNS-MPO | BBB_Score | -logS |
| --- | --- | --- | --- | --- | --- |
| unmetabolized | **HR67** | 5.28 | 3.71 | 4.50 | 5.89 |
| Amide Hydrolysis |  | 4.95  (3.91) | 5.07  (5.45) | 4.44  (4.11) | 5.22  (-0.04) |
| Pyridine *N*-Oxidation |  | 5.36  (safe) | 4.57 | 4.28 | 6.13 |
| N-Hydroxylation of secondary arylamide |  | 5.63  (safe) | 3.78 | 4.11 | 5.06 |
| Hydroxylation of terminal methyl |  | 5.40  (safe) | 4.11 | 3.77 | 5.44 |
| Hydroxylation of aromatic carbon ortho to halide group |  | 5.76  (safe) | 3.50 | 3.75 | 5.44 |
| Reduction of ketone to alcohol |  | 5.49  (safe) | 3.67 | 4.27 | 4.95 |

*B*

| Reaction type | Predicted Metabolite | hERG_AM_ | CNS-MPO | BBB_Score | logS |
| --- | --- | --- | --- | --- | --- |
| unmetabolized | **HR68** | 5.28 | 3.71 | 4.50 | -5.89 |
| Amide Hydrolysis |  | 4.95  (4.25) | 5.07  (4.97) | 4.44  (4.35) | -0.92  (1.60) |
| Pyridine *N*-Oxidation |  | 5.05  (safe) | 4.57 | 4.28 | -6.15 |
| N-Hydroxylation of secondary arylamide |  | 5.08  (safe) | 3.78 | 4.12 | -4.93 |
| Hydroxylation of terminal methyl |  | 5.05  (safe) | 4.11 | 3.77 | -5.44 |
| Hydroxylation of aromatic carbon ortho to halide group |  | 5.32  (safe) | 3.50 | 3.75 | -5.31 |
| Hydroxylation of aromatic carbon meta to halide group |  | 5.23  (safe) | 3.18 | 3.72 | -5.15 |
| Reduction of ketone to alcohol |  | 5.15  (safe) | 3.67 | 4.27 | -4.95 |
